# Effectiveness of Pelvic Floor Muscle and Education-Based Therapies on Bladder, Bowel, Vaginal, Sexual, Psychological Function, Quality of Life, and Pelvic Floor Muscle Function in Females Treated for Gynecological Cancer: A Systematic Review

**DOI:** 10.1007/s11912-024-01586-7

**Published:** 2024-08-23

**Authors:** Marie-Pierre Cyr, Tamara Jones, Robyn Brennen, Udari Colombage, Helena C. Frawley

**Affiliations:** 1 School of Health and Rehabilitation Sciences, The University of Queensland, Therapies Annex, 84a Services Rd, St Lucia, Brisbane, QLD 4067 Australia; 2 Melbourne School of Psychological Sciences, The University of Melbourne, Melbourne, VIC Australia; 3 Department of Physiotherapy, The University of Melbourne, Melbourne, VIC Australia; 4 Department of Physiotherapy, University of South Australia, Adelaide, SA Australia; 5 Department of Physiotherapy, Monash University, Melbourne, VIC Australia; 6 School of Health Sciences, The University of Melbourne, Alan Gilbert Building, 161 Barry St, Carlton, Melbourne, VIC 3010 Australia; 7 The Royal Women’s Hospital, Melbourne; Mercy Hospital for Women, Melbourne, VIC Australia

**Keywords:** Conservative treatment, Education, Gynecological cancer, Pelvic floor, Rehabilitation, Women’s health

## Abstract

**Purpose of Review:**

Gynecological malignancies are prevalent in females, and this population is likely to experience symptoms of pelvic floor disorders and sexual dysfunction. Non-surgical, non-pharmaceutical conservative therapies, namely pelvic floor muscle (PFM) therapies and education-based interventions, could be beneficial for this population. The purpose of this systematic review was to examine the evidence regarding their effectiveness on bladder, bowel, vaginal, sexual, psychological function, quality of life, and PFM function in gynecological cancer populations.

**Recent Findings:**

Six databases were searched to identify studies employing any interventional study design, except case studies, to investigate the effect of PFM therapies, education-based interventions, or combined therapies on any outcome of interest. The search yielded 4467 results, from which 20 studies were included. Of these, 11 (55%) were RCTs, two (10%) were non-RCTs with two groups, and seven (35%) were non-RCTs with a single group. Findings suggest that combined (multimodal) therapies, specifically PFM (active > passive) + education therapies, appear more effective for vaginal, overall pelvic floor, sexual, and PFM function. PFM therapies (active and/or electrostimulation) may improve bladder outcomes. Limited evidence suggests PFM (active) + education therapies may improve bowel function. Conservative therapies may improve psychological function, although available data do not appear to favor a particular therapy. Given the conflicting findings regarding quality of life, no clear conclusions can be made. Interpretation of findings highlighted the importance of intervention dosage, adherence, and supervision for optimal effectiveness. Despite the limitations of the included studies, this review provides new and valuable insights for future research and clinical practice.

**Supplementary Information:**

The online version contains supplementary material available at 10.1007/s11912-024-01586-7.

## Introduction

Gynecological malignancies account for 15–16% of cancer cases in females, totaling an estimation of 1,473,427 new cases globally every year [1]. Females diagnosed with gynecological cancer often develop symptoms of pelvic floor disorders and sexual dysfunction because of cancer and its treatment [2, 3]. Surgery, which may involve the removal of the uterus or one or two ovaries, and radiotherapy, including brachytherapy and external-beam radiation therapy, are commonly administered as part of individual treatment plans, and can cause direct and indirect changes in pelvic floor tissues [4]. These changes potentially contribute to the development, maintenance, or worsening of symptoms of pelvic floor disorders and sexual dysfunction [5]. Among gynecological cancer survivors, up to 76% of females suffer from urinary incontinence, 34% from fecal incontinence, 58% from dyspareunia, 17% from pelvic organ prolapse, and 75% from sexual dysfunction [2], which can prompt psychological distress and affect quality of life [6, 7].

Given the high prevalence and repercussions of symptoms of pelvic floor disorders and sexual dysfunction in gynecological cancer populations, many studies have investigated different interventions to alleviate their burden. There is an advantage in considering non-pharmaceutical conservative therapies for this population as hormone replacement therapy is controversial in females with hormone-receptor positive cancer and there are potential undesired effects with pharmaceutical products [8]. A recent systematic review examined the effectiveness of pelvic floor muscle (PFM) therapies in gynecological cancer survivors [9]. At the time of the review, only seven studies, with varying methodological quality, all of which involved delivery of therapies after cancer treatment (i.e., rehabilitation), were identified [9]. Among included outcomes, only sexual function and quality of life had sufficient data to form conclusions: low-to-moderate evidence was found to support the effectiveness of PFM training or dilator therapy in improving sexual function and quality of life in cervical cancer survivors [9]. It is crucial to review recent evidence to assess whether new findings add to or modify existing knowledge. This should include the examination of a range of outcomes, including bladder, bowel, vaginal, sexual, psychological function, quality of life, and PFM function [9] to document the proposed multifaceted effectiveness of PFM therapies [10]. In addition, given the interest in implementing interventions for preventive purposes, recent studies may have delivered PFM therapies before or during cancer treatment, and deserve examination to determine if these can be beneficial.

In addition to PFM therapies, education-based therapies that can indirectly influence the pelvic floor may improve specific outcomes and are often delivered in combination with PFM therapies. The objective of this systematic review was to examine the evidence regarding the effectiveness of non-surgical, non-pharmaceutical conservative therapies, namely PFM therapies and education-based therapies, on bladder, bowel, vaginal, sexual, psychological function, quality of life, and PFM function in gynecological cancer populations.

## Materials and Methods

### General Methodology and Search Strategy

This systematic review was undertaken and reported in accordance with Preferred Reporting Items for Systematic Reviews and Meta-Analyses (PRISMA) [11] and the Synthesis Without Meta-analysis (SWiM) Reporting Guideline [12]. Informed by a prior review [9] and in collaboration with a medical librarian, the search strategy was created based on PICO eligibility criteria. Studies were searched in six databases (Medline, Embase, CINAHL, Cochrane Library, PsycINFO, and Emcare) from their respective inception to December 2023. Supplementary Information 1 provides the details of the search strategy.

### Study Selection

Any original, quantitative, prospective study, including randomized controlled trials (RCTs), non-randomized controlled trials (non-RCTs), interventional cohort or case series (pre-post) studies, and pilot studies, except case studies, were included. Papers written in English or French, and published in a peer-reviewed journal were eligible. Eligibility criteria as per the PICOT approach (P: population, I: intervention, C: comparison, O: outcome, T: time frame; timing of delivery) are presented in Table 1. One reviewer (MPC, RB, or UC) independently selected the studies by screening titles and abstracts, followed by full texts. Two reviewers (HF and TJ) verified the eligibility of included studies. Any disagreement between the reviewers for selection of studies was solved through team discussion.

**Table 1:** Eligibility Criteria for Study Selection

Populations	• Gynecological cancer: Studies were included if at least 75% of the participants were adult females (aged ≥ 18 years) who had either been diagnosed with or undergone any treatment for any type of gynecological cancer, i.e., uterine, cervical, endometrial, ovarian, fallopian tube, peritoneal, vaginal, or vulval cancer
Interventions	• Pelvic floor muscle (PFM) therapies: Studies were included if they involved non-surgical, non-pharmaceutical interventions that targeted the pelvic floor soft tissues, e.g., performance of voluntary activation and relaxation of PFM, electrical or magnetic stimulation, manual therapy, dilator therapy, or desensitization techniques. PFM therapies taught or supervised by any health professionals, delivered in either individual or group setting, and in person or remotely via telecommunication were eligible • Education-based therapies: Studies were included if they involved non-surgical, non-pharmaceutical interventions that targeted cognitive aspects related to the pelvic floor. Therapies had to have a plausible explanation that they could indirectly influence the function of the pelvic floor, i.e., bladder, bowel, vaginal, or muscle function. Education-based therapies which provided information about the pelvic floor structure, function, or exercise; addressed emotional, psychological, behavioral, or sexual aspects related to the cancer or cancer treatment repercussions, e.g., advice for vaginal lubricant, moisturizer, or body scanning, were considered for inclusion. Education-based therapies delivered by any health professionals and in any form, such as counselling, psychoeducation, cognitive-behavioral therapy (CBT), or mindfulness-based therapy, and administered in either individual or group setting, and in person or remotely via telecommunication were eligible • Combined therapies: Studies were included if they involved combinations of PFM therapies and education • Other interventions: Studies were considered for inclusion if other interventions were used (e.g., general exercise), but they were excluded if a pharmaceutical ingredient or laser therapy was used
Comparison	• Studies with or without any type of comparator group were included
Outcomes	• Studies were included if they had any outcome related to bladder, bowel, vaginal, overall pelvic floor (i.e., combination of bladder, bowel, and vaginal), sexual, psychological function, quality of life, or PFM function. Eligibility included outcomes assessed with patient-reported outcome measures, e.g., self-reported questionnaires for symptoms related to bladder, bowel, vaginal, overall pelvic floor, sexual, psychological function, or quality of life; clinician-reported outcome measures, e.g., digital palpation; and instrument-measured outcomes, e.g., ultrasound imaging, magnetic resonance imaging, shear-wave elastography, electromyography, or manometry for PFM function
Time frame; timing of delivery	• Time frame: Studies were included if outcomes were assessed before and after the intervention, at any follow-up timepoint. Included follow-up time frames were defined as follows: short-term follow-up as either immediately after or within 2 weeks post-intervention, medium-term follow-up as beyond 2 weeks but less than 12 months post-intervention, and long-term follow-up as 12 months or more post-intervention • Timing of delivery: Studies that provided intervention at any timepoint in relation to cancer treatment were included: prehabilitation (before cancer treatment), during cancer treatment, rehabilitation (after cancer treatment), or mixed (intervention delivery over more than one cancer treatment time phase)

*CBT* cognitive-behavioral therapy, *PFM* pelvic floor muscle

### Data Extraction

Two reviewers (reviewer 1: MPC, RB, or UC, and reviewer 2: TJ) independently extracted data using a customized data extraction sheet which was created, and pilot tested by the team (MPC, HF, RB, UC, and TJ). Extracted data included study details, population characteristics, intervention arms, including the treatment group (TG) intervention and the comparator group (CG), outcomes as outlined in the eligibility criteria of this systematic review and their associated outcome measures, adverse events, assessment timepoints, and results. If data were not interpretable, attempts were made to contact the authors for clarification. The Template for Intervention Description and Replication (TIDieR) checklist [13] was used to retrieve information about PFM therapies and education-based therapies. Any disagreement between reviewers for data extraction was solved through team discussion.

### Risk of *Bias* and Quality Assessment

The risk of bias of included studies was assessed independently by two reviewers (reviewer 1: MPC, RB, or UC, and reviewer 2: TJ) using the Cochrane risk-of-bias (RoB) tool for RCTs [14], the Newcastle–Ottawa Scale (NOS) for non-RCTs with two groups [15], or Joanna Briggs Institute (JBI) Critical Appraisal Checklist for Quasi-Experimental Studies for non-RCTs with a single group [16]. The percentage of agreement between reviewers was calculated. Any disagreement between the reviewers for risk of bias or quality assessment were solved through team discussion.

### Data Interpretation and Synthesis

PFM therapies were categorized according to their description in the included studies and grouped for interpretation in order to reduce heterogeneity. PFM therapies were considered ‘active’ if they involved voluntary control of PFM activation by the individual; ‘passive’ if they involved no voluntary control of PFM activation by the individual but rather techniques from external sources aimed at changing pelvic floor tissues, such as manual therapy, dilator therapy, desensitization techniques, direct application of vaginal lubricant, moisturizer; or ‘electrostimulation’ if they involved electrical current to stimulate PFM activity. Assessment timepoints were categorized as follows: ‘baseline’ for the initial assessment before intervention, ‘follow-up timepoint’ for subsequent assessments, and ‘end timepoint’ when there was only one subsequent assessment, or this was explicitly identified as such by the authors when more than one follow-up assessment was performed. To aid in interpretating the results of individual studies, data values and their significance, or narrative descriptions, were used to indicate the ‘direction of findings’. Considerations for interpretation were summarized to provide further interpretation of the findings. For the TIDieR checklist, each described item was assigned a score of’1’, with a maximum achievable score of 12 for each study. A score of ‘12’ indicated that the description of the TG intervention satisfied all items on the checklist.

For this systematic review, a summary narrative synthesis approach was adopted, and thematic categorization was employed to allow a comprehensive review of the available evidence [12]. Findings are presented according to each outcome of interest, namely bladder, bowel, vaginal, overall pelvic floor, sexual, psychological function, quality of life, and PFM function. Additionally, the level of evidence for each outcome was rated according to the study design using the Oxford Centre for Evidence-Based Medicine (OCEBM) 2011 Levels of Evidence framework [17], and grouped according to the type of TG intervention, which informed the overall interpretation and recommendations.

## Results

### Selected Studies

**Fig. 1:**
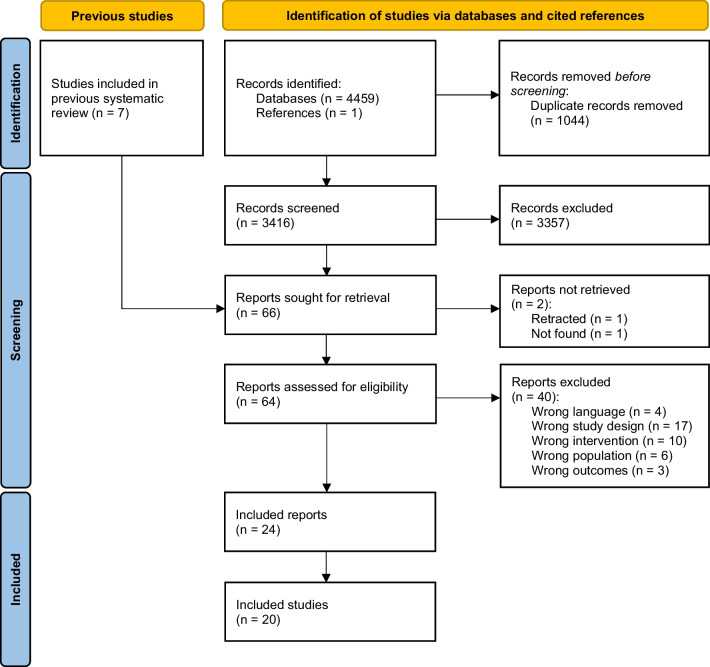
PRISMA Flow Chart

Figure 1 presents the flow chart of this systematic review. A total of 4467 records were identified and screened. After screening titles and abstracts, 64 full texts were assessed for eligibility. Twenty-four full texts were included, representing 20 original, individual studies conducted in gynecological cancer populations, of which 11 (55%) studies were RCTs (*n* = 1288 females), two (10%) were non-RCTs with two groups (*n* = 183 females), and seven (35%) were non-RCTs with single group (*n* = 230 females).

Table 2 shows the characteristics of included studies. Studies are presented according to the study design (RCTs followed by non-RCTs), type of TG intervention (combined therapies followed by PFM and education-based therapies), arranged from the most recent to the oldest studies. The age range of females spanned from 32 to 66 years. The most represented gynecological cancer was cervical cancer, with 1326 females, representing 78% of gynecological cancer populations. One study (5%) examined a prehabilitation intervention (before cancer treatment), 10 (50%) rehabilitation interventions (after cancer treatment), and nine (45%) mixed interventions (delivery over more than one cancer treatment time phase). Full description of TG interventions as reported by the study authors and according to the TIDieR checklist can be found in Supplementary Information 2. The number of satisfied items from the TIDieR checklist, which was used to assess the quality of reporting of TG interventions, ranged between 5–11 (out of 12), representing 42%-92% of the total checklist items. Based on the available assessment timepoints in included studies, the majority used follow-up time frame related to short- and medium-term effectiveness, and only one study (5%) provided results for long-term effectiveness [10]. Studies reported intervention effect on bladder (*n* = 11, 55%), bowel (*n* = 6, 30%), vaginal (*n* = 6, 30%), overall pelvic floor (*n* = 2, 10%), sexual (*n* = 10, 50%), psychological (*n* = 8, 40%) function, quality of life (*n* = 8, 40%), and PFM function (*n* = 7, 35%).

**Table 2:** Characteristics of Included Studies

Authors; year; country	Study design; sample size	Type of cancer, n (%)	Age, years Mean (SD)	Category of intervention for timing of delivery; time elapsed since cancer diagnosis or last cancer treatment to pre-intervention (baseline) assessment	Treatment Group (TG) details: type; name; short description	Comparator Group (CG) name (short description)	Assessment timepoints; follow-up time frame	Reported outcomes
Sun et al. [18]; 2023; China	RCT; *n* = 130	Cervical, 130 (100%)	40 (8)	Rehabilitation; Time since cancer treatment: NR	**PFM therapy (active) + education**; Health education based on the integrated theory of health behavior change in pelvic floor rehabilitation; Education manuals, health lectures, videos, and WeChat application. Home program, unweighted PFM exercises, 5-s contraction 5–10-s rest, 15–20 min once per day, and intra-vaginal weights 3–5-s contraction and 8–10-s rest, 20 reps, 3 sets per day. Duration of therapy: NR (likely 3 months)	Routine nursing care (admission assessment, preoperative intestinal and vaginal preparation, psychological intervention, health education, postoperative catheter nursing, life nursing, complication prevention, discharge, and follow-up guidance)	Baseline: post-surgery (pre-intervention); End timepoint: post-surgery, 3 months after discharge (immediately post-intervention); Short-term follow-up	Bladder function; Bowel function; Vaginal function; Overall pelvic floor function; Psychological function
Zong et al. [19]; 2022; China	RCT; *n* = 166	Cervical, 166 (100%)	42–43 (4)	Mixed; Time since cancer diagnosis: NR; Time since cancer treatment: 3 days pre-surgery, 3 and 10 days post-surgery	**PFM therapy (active) + clean intermittent self-catheterization**; PFM training combined with clean intermittent self-catheterization; PFM exercises 3 days pre-surgery, and diastolic and contractile exercises of the vagina, urethra and anal sphincter, 10-s contraction with inhalation followed by 10-s relaxation with exhalation, 20 min, 3 times per day, performed on day 4 post-surgery. Duration of therapy: 3 days pre-surgery + 7 days post-surgery (10 days)	Clean intermittent self-catheterization (+ education on urinary retention and urinary incontinence)	Baseline: 3 days pre-surgery (pre-intervention); Follow-up timepoint 1: 3 days post-surgery (during intervention); Follow-up timepoint 2: 10 days post-surgery (immediately post-intervention); Short-term follow-up	Bladder function; Quality of life
Li et al. [20]; 2019; China	RCT; *n* = 91	Cervical, 91 (100%)	NR	Rehabilitation; Time since cancer treatment: 11 days	**PFM therapy (electrostimulation) + intermittent catheterization + bladder function training**; Traditional bladder function training and low frequency electrical stimulation; 2 sets of electrical stimulation parameters were chosen for 2 treatment principles: electrical stimulation frequency of 35Hz and pulse width of 200μs (neuromuscular repair program, group A) or frequency of 1Hz and pulse width of 270μs (endorphins analgesia program, group B). Electrical stimulation was performed 2 times per day for 15–30 min from day 11 post-surgery Duration of therapy: 3 days	Intermittent catheterization + bladder function training	Baseline: 11 days post-surgery (pre-intervention); End timepoint: 14 days post-surgery (immediately post-intervention); Short-term follow-up	Bladder function; PFM function
Li et al. [21]; 2016; China	RCT; *n* = 226	Cervical, 226 (100%)	46 (9)	Rehabilitation; Time since cancer treatment: unclear (before discharge)	**PFM therapy (active) + nursing education + Yoga**; Home-based, nurse-led health program; PFM training, 3–5 times per day, 10 repetitions of 10-s contraction and 10-s relaxation. Yoga, 30-min, 2 times per day. Duration of therapy: 6 months	Conventional nursing care (including education on medications, nutrition, and how to care for and strengthen the reproductive tract)	Baseline: within 7 days post-surgery (pre-intervention); End timepoint: immediately post-intervention; Short-term follow-up	Sexual function; Quality of life
Rutledge et al. [22]; 2014; United States	RCT; *n* = 40	Uterine, 24 (60%) Ovarian, 9 (23%) Cervical, 5 (13%) Other, 2 (5%)	57 (7)	Rehabilitation; Time since cancer treatment (surgery): median 2.5 (range 1–5) in years	**PFM therapy (active) + education**; PFM exercise training; Home-based program, 5-s PFM contraction × 10 repetitions, 3 times per day, with handout and instruction describing behavioral management tips for urinary incontinence. Duration of therapy: 12 weeks	Usual care (no intervention)	Baseline: pre-intervention; End timepoint: 1 week post-intervention; Short-term follow-up	Bladder function; PFM function
Yang et al. [23]; 2012; Korea	RCT; *n* = 28	Cervical, 26 (93%) Endometrial, 2 (7%)	52 (8)	Rehabilitation; Time since cancer treatment (surgery): median 1.2 (range 1–5) in years	**PFM therapy (active) + education + core-strengthening program + hip muscle stretching exercises**; Pelvic floor rehabilitation program; 4 weekly sessions, including 45-min exercise session (including biofeedback, core exercises targeting PFM and transverse abdominus, and hip muscle stretching exercises) and 30-min counselling session (PFM evaluation, lifestyle advice, encouragement etc.). Biofeedback: 40 cycles with 10-s of activity followed by 20-s of relaxation. Core exercises: Strengthening for the PFM and transverse abdominis muscles and stretching exercises for muscles attached to the pelvic girdle. Home program: 10-s PFM contraction followed by 4-s rest × 10 repetitions, followed by 1-min pause then 10 or more fast contractions for 20–30 s, 6 times per day. Duration of therapy: 4 weeks	Leaflet with home-based PFM exercise, lifestyle advice and a telephone number for further explanations	Baseline: pre-intervention; End timepoint: 1 week post-intervention; Short-term follow-up	Bladder function; Bowel function; Sexual function; Quality of life; PFM function
Cerentini et al. [24]; 2019; Brazil	RCT; *n* = 88	Cervical, 88 (100%)	44 (12)	Mixed; Time since cancer diagnosis/treatment: NR	**PFM therapy (passive)**; Vaginal dilators during and after brachytherapy; Size given according to patients’ vaginal size, and patients were oriented to use the device 10–15 min, 4 times a week 2 starting moments: (1) concomitant with brachytherapy, (2) 4 weeks after the end of brachytherapy. Duration of therapy: 2–3 months	Standard guidance from nursing team care (including re advice to use dilators)	Baseline 1: post-external-beam- radiotherapy, pre-brachytherapy (pre-intervention); Baseline 2 / Follow-up timepoint 1: immediately post-brachytherapy (pre-intervention for some participants and post-intervention for some participants); Follow-up timepoint 2: 3 months post-brachytherapy (post-intervention); Short-term follow-up	Bladder function; Bowel function; Vaginal function; Quality of life; PFM function
Jiang et al. [25]; 2023; China	RCT; *n* = 100	Cervical, 100 (100%)	NR	Unclear (likely mixed); Time since cancer diagnosis/ treatment (surgery): unclear	**Education**; Routine nursing care + continuing nursing; Multidisciplinary intervention (gynecologic surgeon, nurses, psychiatrist, psychological consultant, rehabilitation therapists) with WeChat online education videos, 20-min seminars (including information about PFM exercise), and guidance for couples only. Duration of therapy: 3–6 months	Routine nursing care (psychological counselling, PFM exercise, vulval skin care, dietary advice, pre- and post-op exercise, nursing monitoring)	Baseline: unclear (likely post-surgery) (pre-intervention); End timepoint: 4 months after discharge (3–6 months post-intervention); Unclear (likely short-term follow-up)	Quality of life
Schofield et al. [26]; 2020; Australia	RCT; *n* = 318	Endometrial/uterine, 162 (52%) Cervical, 130 (41%) Vulval, 26 (8%) Ovarian, 16 (5%) Vaginal, 12 (4%) Fallopian tube, 6 (2%) Other, 2 (1%)	56 (14)	Mixed; Time since cancer diagnosis/treatment: NR	**Education**; Psycho-education nurse- and peer-led psycho-educational intervention; 3–4 nurse-led (30–60 min) and 3–4 peer-led sessions, including ‘demonstration’ of dilator use and PFM exercises. Duration of therapy: unclear (likely 2–4 weeks)	Usual care (cancer council booklet on specific cancer type, treatment, and its side-effects)	Baseline: pre-radiotherapy (pre-intervention); End timepoint 1: immediately before first radiotherapy (first nurse-led session); End timepoint 2: 2–4 weeks post-radiotherapy (last nurse-led session); Follow-up timepoint 3: 3 months post-radiotherapy (post-intervention); Follow-up timepoint 4: 6 months post-radiotherapy (post-intervention); Follow-up timepoint 5: 12 months post-radiotherapy (post-intervention; Short- and medium-term follow-up	Vaginal function; Sexual function; Psychological function; Quality of life
Du et al. [27]; 2020; China	RCT; *n* = 69	Cervical, 69 (100%)	34–37 (11)	Unclear (likely mixed); Time since cancer diagnosis/treatment: NR	**Education**; Empowerment education-based nursing interventions for sexual function; Multidisciplinary intervention (doctor, psychological counselor, charge nurse, primary nurses) with 5 modules (clarify problems, expression, set goals, planning, outcome evaluation), including ‘demonstration’ for anal contraction exercises. Duration of therapy: NR	Conventional nursing interventions for sexual function (e.g., health education, psychological counselling, diet intervention, guidance on postoperative recovery, and sexual life, etc.)	Baseline: pre-surgery (unclear if pre-intervention or during intervention); Follow-up timepoint 1: 1 month post-surgery (likely during intervention); Follow-up timepoint 2: 2 months post-surgery (likely during intervention); Follow-up timepoint 3: 3 months post-surgery (likely during intervention); Follow-up timepoint 4: 4 months post-surgery (likely during intervention); Follow-up timepoint 5: 5 months post-surgery (likely during intervention); Follow-up timepoint 6: 6 months post-surgery (likely during or after intervention); Unclear (likely short-term follow-up)	Sexual function; Psychological function; Quality of life
Robinson et al. [28]; 1999; Canada	RCT; *n* = 32	Cervical, 24 (75%) Endometrial, 8 (25%)	47 (range 28–73)	Mixed; Time since cancer diagnosis/ treatment: NR	**Education**; Psychoeducation group sessions; 2 × 1.5 h psychoeducation group sessions and booklet with information on lubricants, dilators, PFM exercises. Duration of therapy: 3 h	Same booklet as in TG	Baseline: during radiotherapy (unclear for intervention timeline); Follow-up timepoint 1: 3 months after diagnosis (unclear for intervention timeline); Follow-up timepoint 2: 6 months after diagnosis (unclear for intervention timeline); Follow-up timepoint 3: 9 months after diagnosis (unclear for intervention timeline); Follow-up timepoint 4: 12 months after diagnosis (unclear for intervention timeline); Unclear	Sexual function; Psychological function
Li et al. [29]; 2023; China	Non-RCT; *n* = 120	Cervical, 120 (100%)	32–34 (6)	Mixed; Time since cancer diagnosis/treatment: NR	**PFM therapy (active + electrostimulation) + routine care**; Pelvic floor rehabilitation exercise + routine care; Bioelectrical stimulation: 30 min per day for 7 days (from day 3 post-op) at a frequency of 20Hz and a current 40–75 mA. Pre-surgery PFM training: 3–5 sets of 20–30 repetitions (approx. 15–30 min per day)—performed the day before surgery. Post-surgery PFM training: 1–5 sets per day (gradually increased post-surgery) with WeChat online education videos and guidance. Duration of therapy: 8 weeks	Routine care (perioperative psychological counselling to relieve psychological pressure + instructions for preoperative rehabilitation exercise training + postoperative bladder function rehabilitation training—catheterization)	Baseline: pre-intervention (unclear if pre-surgery); Follow-up timepoint 1: 2 weeks post-intervention for some outcome measures; Follow-up timepoint 2: 3 months post-surgery for some other outcomes (unclear for intervention timeline); Short- and medium-term follow-up	Bladder function; Overall pelvic floor function
Tung et al. [30]; 2024; China	Non-RCT; *n* = 63	Cervical, 63 (100%)	49 (11)	Prehabilitation; Time since cancer diagnosis: NR	**Education**; Transtheoretical model-based sexual health education program; Interactive self-help pamphlet with 4 modules (sexual communication and intimacy, physical sexual health adjustment (use of lubricant or vaginal moisturizer), elimination of sexual myths and gender blindness, reconstruction of the sexual self) and one session with nurse educator. Duration of therapy: 10–15 min	Traditional sexual health education (basic health education information pamphlet followed by 10–15-min education talk)	Baseline: pre-intervention; Follow-up timepoint 1: 1 week post-intervention; Follow-up timepoint 2: 6 weeks post-intervention; Short- and medium-term follow-up	Sexual function
Cyr et al. [10, 31–33]; 2020; 2021; 2022a; 2022b; Canada	Non-RCT; *n* = 31	Endometrial, 20 (64.5%) Cervical, 11 (35.5%)	56 (11)	Rehabilitation; Time since cancer treatment: median 38 (Q1 9; Q3 70) in months	**PFM therapy (active + passive) + education**; Multimodal pelvic floor physiotherapy; 12 weekly individual 60-min sessions involving education (e.g., pathophysiology and management of dyspareunia), manual therapy (20–25 min per session involving stretching, myofascial release, trigger/tender point pressure and massage), pelvic floor muscle exercises using biofeedback (20 min per session, included maximal contractions, podium contractions or reversed podium contractions, rapid contractions and one minute sustained maximal contraction) and home exercises (5 times per week, similar exercise to those performed under biofeedback), which included the use of a dilator (3 times per week). Duration of therapy: 12 weeks	NA	Baseline: pre-intervention; Follow-up timepoint 1: 2 weeks post-intervention; Follow-up timepoint 2: 12-months post-intervention; Short- and long-term follow-up	Bladder function; Bowel function; Vaginal function; Sexual function; Psychological function; PFM muscle
Brennen et al. [34]; 2023; Australia	Non-RCT; *n* = 36	Endometrial/uterine, 25 (69%) Cervical, 7 (19%) Ovarian, 4 (11%)	58 (IQR 17)	Rehabilitation; Time since cancer treatment: median 17.5 (IQR 27) in months	**PFM therapy (active) + education**; Telehealth-delivered pelvic floor muscle training; Fortnightly telehealth sessions (7 total; 30–60 min) and daily PFM training program using femfit intravaginal biofeedback device (3–6 sets of 6–10 maximal contractions, 6–10 fast contractions, 3 endurance contractions and 3 contractions with cough). Education-based therapies were also provided alongside PFM training depending on the participant’s symptoms. Duration of therapy: 12 weeks	NA	Baseline: pre-intervention; Follow-up timepoint 1: 1–2 weeks post-intervention; Follow-up timepoint 2: 3 months post-intervention; Short- and medium-term follow-up	Bladder function; Bowel function; PFM function
Bernard et al. [35]; 2021; Canada	Non-RCT; *n* = 8	Endometrial, 8 (100%)	66 (range 39–76)	Rehabilitation; Time since cancer treatment: mean 43 (range 19–55) in months	**PFM therapy (active) + education**; In-home rehabilitation program; PFM training program (completed with the Elvie Trainer, an intravaginal dynamometer, and its mobile application), bladder training regime, and counselling on lifestyle habits. Participants received their daily exercise program through the mobile app and weekly telephone follow-ups by a physiotherapist to offer personalized advice. Duration of therapy: 12 weeks	NA	Baseline: pre-intervention; End timepoint: 2 weeks post-intervention; Short-term follow-up	Bladder function; PFM function
Sacomori et al. [36]; 2020; Chile	Non-RCT; *n* = 49	Cervical, 49 (100%)	44 (11)	Mixed; Time since cancer diagnosis: NR; Time since cancer treatment: up to 1 month post-radiotherapy	**PFM therapy (active)**; Non-supervised home-based PFM exercises; A single 30-min physical therapy session plus exercises 2 times per day at home (recommended: 8 × 6-s with 10-s rest maximal voluntary contractions, 8 × 1-s maximal voluntary contractions followed by relaxation, and voluntary precontraction of the pelvic floor before activities that increased intra-abdominal pressure). An educational flyer and an audio recording with the instructions for the exercises were provided. Duration of therapy: NR (likely 1 month)	NA	Baseline: pre-radiotherapy (pre-intervention); End timepoint: 1 month post-radiotherapy (unclear for intervention timeline); Unclear (likely short-term follow-up)	Bladder function; Bowel function; PFM function
Bober et al. [37]; 2018; Hungr et al. [38]; 2020; United States	Non-RCT; *n* = 53	Ovarian, 53 (100%)	56 (10)	Mixed; Time since cancer diagnosis: mean 6 (SD 5) in years; Time since cancer treatment: NR	**Education**; Sexual therapy and rehabilitation after treatment for ovarian cancer (psychoeducational intervention); A single 3.5-h group session (including sexual health information, education about treatment-related sexual problems, communication strategies, relaxation training and body awareness, mindfulness-based cognitive therapy and individual-based activity planning) with 15-min booster telephone call after 1 month. Duration of therapy: 3.5 h	NA	Baseline 1: 6–8 weeks before the intervention (pre-intervention); Baseline 2: immediately before the education session (pre-intervention); Follow-up timepoint 1: 2 months post-intervention; Follow-up timepoint 2: 6 months post-intervention; Medium-term follow-up	Sexual function; Psychological function
Brotto et al. [39]; 2012; Canada	Non-RCT; *n* = 31	Endometrial, 20 (65%) Cervical, 8 (26%) Both, 3 (10%)	54 (8)	Rehabilitation; Time since cancer treatment: NR	**Education**; Psychoeducational intervention; 3 × 90-min sessions (one per month) including education on causes of sexual difficulties, cognitive challenging of maladaptive sexual beliefs, prevalence of sexual dysfunction after cancer, body image and mindfulness exercises, arousal-enhancing techniques, and pelvic floor health. Duration of therapy: 3 months	NA	Baseline: pre-intervention; Follow-up timepoint 1: immediately post-intervention; Follow-up timepoint 2: 6 months post-intervention; Short- and medium-term follow-up	Vaginal function; Sexual function; Psychological function
Brotto et al. [40]; 2008; Canada	Non-RCT; *n* = 22	Cervical, 13 (59%) Endometrial, 9 (41%)	49 (range 26–68)	Rehabilitation; Time since cancer treatment: mean 54 (range 6–115) in months	**Education**; Brief mindfulness-based cognitive behavioral intervention; 3 × 60-min audio-recorded (one per month) including factors of sexuality, cognitive challenging of maladaptive sexual beliefs, prevalence rates of sexual difficulty following cancer and its treatment, connection between sexual relationship and sexuality, and body image and sexuality, techniques to augment sexual arousal, loosening exercises designed to strengthen the larger muscles of the body; using self-sensate focus to tune in to sexual arousal; potential role of erotica, fantasy, and vibrators in augmenting natural sexual arousal response. As well as 5–7 h of personal time per month. Duration of therapy: 3 months	NA	Baseline: pre-intervention; End timepoint: immediately post-intervention; Short-term follow-up	Vaginal function; Sexual function; Psychological function; Quality of life

*CG* comparator group, *IQR* interquartile range, *NA* not applicable, *NR* not reported, *PFM* pelvic floor muscle, *Q1* first quartile, *Q3* third quartile, *RCT* randomized controlled trial, *SD* standard deviation, *TG* treatment group

### Risk of *Bias* and Quality Assessment

Tables 3, 4 and 5 show the item and overall rating on the risk of bias or quality assessment tool of all included studies according to study design. For all RCTs, the overall risk of bias was assessed as ‘some concerns’ using the RoB tool. For non-RCTs with two groups, one study was of ‘poor quality’ and one of ‘fair quality’ according to the NOS. For non-RCTs with single group, the percentage of satisfied criteria ranged from 4 to 6 out of 7 on the JBI checklist. The agreement between reviewers for all scored items was 89%.

**Table 3:**
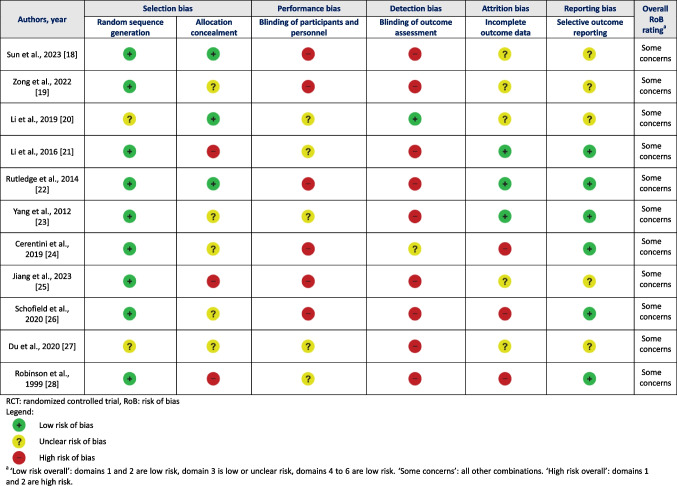
RoB Domains of Reviewed RCTs

**Table 4:**
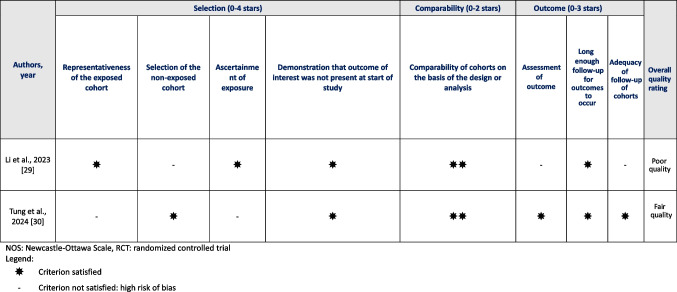
NOS Items of Reviewed Non-RCTs with Two Groups

**Table 5:** JBI Checklist for Quasi-Experimental Study Items of Reviewed Non-RCTs with Single Group

Authors, year	Is it clear what is the ‘cause’ and what is the ‘effect’ (i.e., there is no confusion about which variable comes first)?	Were the participants included in any comparisons similar?	Were the participants in the comparison receiving similar treatment/care other than the exposure or intervention of interest?	Was there a control group?	Were there multiple measurements of the outcome both pre and post the intervention/exposure?	Were follow- up complete and, if not, were differences between groups in terms of their follow-up adequately described?	Were the outcomes of participants included in any comparisons measured in the same way?	Were outcomes measured in a reliable way?	Was appropriate statistical analysis used?	Number of satisfied criteria among applicable criteria (n satisfied/n applicable)
Cyr et al., 2020, 2021, 2022a, 2022b [10, 31–33]	Yes	NA	NA	No	Yes	Yes	Yes	Yes	Yes	6/7
Brennen et al., 2023 [34]	Yes	NA	NA	No	Yes	Yes	Yes	Yes	Yes	6/7
Bernard et al., 2021 [35]	Yes	NA	NA	No	Yes	Yes	Yes	Yes	Yes	6/7
Sacomori et al., 2020 [36]	Yes	NA	NA	No	Yes	No	Yes	Yes	Yes	5/7
Bober et al., 2018 [37]; Hungr et al., 2020 [38]	Yes	NA	NA	No	Yes	Yes	Yes	Yes	Unclear	5/7
Brotto et al., 2012 [39]	Yes	NA	NA	No	Yes	Yes	Yes	Yes	Unclear	5/7
Brotto et al., 2008 [40]	Yes	NA	NA	No	No	Yes	Yes	Yes	Unclear	4/7

*JBI* Joanna Briggs Institute, *NA* not applicable, *RCT* randomized controlled trial

### Outcomes

Supplementary Information 3 presents the findings of individual studies on bladder, bowel, vaginal, overall pelvic floor, sexual, psychological function, quality of life, and PFM function, along with considerations for interpretation. Only five studies out of the 20 included studies (25%) reported data on adverse events related to the TG intervention. Among these, one study reported no adverse events related to a PFM training program [36] and four studies reported temporary minor adverse effects experienced by a small number of participants [19, 20, 31, 34]. These included small amount of vaginal bleeding [34], irritation related to a vaginal product, and shoulder pain [31] for PFM therapies involving exercises with insertion of a device or dilator into the vaginal cavity, as well as urinary retention and dysuria for therapies involving catheterization [19, 20].

#### Bladder Function

Six RCTs provided data on bladder function [18–20, 22–24], one of which presented results that were non-interpretable [24]. Based on two RCTs with reasonable sample sizes, PFM therapies (active or electrostimulation) reduced post-void residual (PVR) [19, 20]. An improvement in both urogenital distress (UDI-6) and urinary incontinence impact (UIQ-7) following a combination of PFM therapy (active) + education was found in another recent RCT (*n* = 130) [18], although a sample size calculation was not reported and some aspects of intervention dosage were unclear. Only one RCT identified a primary outcome which informed a sample size calculation (*n* = 40) [22], however the outcome selected was a post-intervention measure only, and no difference between groups was found following a combination of PFM therapy (active) + education with low supervision. An older RCT showed no difference between groups in bladder function (APFQ) following a combination of PFM therapy (active) + education + other exercises [23], however sample size was very small (*n* = 28) with no sample size calculation provided and the intervention appeared to be low intensity. Intervention descriptions were limited for the majority of RCTs.

One non-RCT reported findings from a two-group comparison indicating improvement in favor of TG for bladder emptying and bladder compliance outcomes (however the two groups were quite imbalanced in number: *n* = 76 for TG vs *n* = 44 for CG); but not for urethral closure pressure following PFM therapy (active + electrostimulation) [29]. Four studies reported results of single-group pre-post comparisons [10, 31, 34–36], ranging from very small (*n* = 8) to medium (*n* = 49) sample size. Among these, two studies stated a priori they were feasibility or pilot studies, and they achieved their pre-defined outcome indicating that the study or intervention was feasible [31, 34]. Three studies provided PFM (active ± passive) + education therapies, of which two demonstrated a significant improvement in bladder function (ICIQ-UI SF, pad test, 3-day bladder diary) from baseline to post-intervention [31, 35] and 12-month post-intervention [10], and one a non-significant improvement in bladder function (prevalence of urinary incontinence, ICIQ-UI SF) from baseline to post-intervention [34], although no studies were powered for these outcomes. One study did not show improvement in bladder function (ICIQ-UI SF) after PFM therapy (active) [36]; details of intervention were unclear, and the study had high attrition, whereas the other three studies [10, 31, 34, 35] reported their interventions clearly and attrition rates were lower.

#### Bowel Function

Three RCTs provided data on bowel function [18, 23, 24]; results from one RCT were not interpretable [24] and the other two presented inconsistent findings [18, 23]. An improvement in both colorectal-anal distress (CRADI-8) and colorectal-anal impact (CRAIQ-7) following a combination of PFM therapy (active) + education was found in one recent RCT [18]. Even though this study had a reasonable sample size (*n* = 130) [18], no sample size calculation was reported and intervention reporting was limited in detail, as previously reported in the bladder function outcome results for this study. An older study that provided PFM therapy (active) + education + other exercises did not show a difference in bowel symptoms between groups [23], however, the very small sample size (*n* = 28) and more recent study may be factors in this negative finding.

Three studies reported results of single-group pre-post comparisons [31, 34, 36]. One study provided PFM (active ± passive) + education therapies and observed significant improvement in bowel function (ICIQ-B) [31], whereas another study showed non-significant improvement in bowel function (ICIQ-B and prevalence of fecal incontinence) post-intervention [34]; both followed the same direction of findings as for bladder function outcome. No improvement in prevalence of anal incontinence was found following PFM therapy (active) in the other study [36]; as mentioned in bladder outcome results, details of intervention were unclear, and the study had high attrition.

#### Vaginal Function

Three RCTs provided data on vaginal function [18, 24, 26]. The most recent study identified an improvement in pelvic organ prolapse (POP) symptoms and impact (POPDI-6 and POPIQ-7) following PFM therapy (active) + education, although limitations of this study are as reported in bladder and bowel function outcome results above [18]. The other two RCTs, with *n* = 88 [24] and *n* = 318 [26], the latter corresponding to the largest study included in this review, investigated the effect of therapies including dilator therapy on vaginal stenosis and dimensions (instrument-measure outcome, LENT SOMA Scale); this was the primary outcome in Cerentini et al. [24]. Neither study detected a significant difference between groups. These results could have been affected by low adherence to the therapy [24], variation in vaginal dilator management among participants or lack of supervision, or timing of the intervention – during radiotherapy – in an heterogenous population [26], which may have made detection of a response to therapy difficult.

Three studies reported results of single-group pre-post comparisons [10, 31, 39, 40]. The study that provided a combination of PFM therapy (active + passive) + education [10, 31] demonstrated improvement in a range of vaginal signs and symptoms which were maintained 12-month post-intervention, with intervention details well reported. Two studies provided education interventions alone: psycho-education [39] and mindfulness-based cognitive behavioral intervention [40], neither of which showed improvement, and intervention descriptions were scant.

#### Overall Pelvic Floor Function

One recent RCT provided data on overall pelvic floor function [18]. This study showed a reduction in pelvic floor distress (PFDI-20) and impact on quality of life (PFIQ-7), which were listed as the primary outcomes for the study, following a combination of PFM therapy (active) + education [18]. Results followed the same direction of findings as for bladder, bowel, and vaginal function of the same study [18].

One two-group non-RCT provided PFM therapy (active + electrostimulation) and also showed a reduction in symptoms (pelvic floor distress [PFDI-20]) [29], supporting improvement observed in instrument-measured outcomes such as PVR, bladder compliance, and bladder detrusor systolic pressure [29]; but no primary outcome was stated. Intervention descriptions in both studies were scant.

#### Sexual Function

Five RCTs provided data on sexual function [21, 23, 26–28]. Among these, two provided a combination of PFM therapy (active) + education + other, and both showed an improvement in sexual function (FSFI, APFQ), despite quite different sample sizes (*n* = 226 vs n = 28) [21, 23]; neither of these studies were powered for this outcome. Three RCTs provided education and findings were inconsistent [26–28]: one observed an improvement in sexual function (FSFI) (*n* = 69) [27], and the other two found no differences between groups (SVQ, Sexual History Form) (*n* = 318 and *n* = 32) [26, 28], of which one listed sexual function as the primary outcome [28]. The intervention dosage was either unclear [27] or low, and with high baseline outcome measure variance [26, 28]; follow-up timepoints were unclear [27, 28]; and one study included participants with different gynecological cancers who underwent radiotherapy during the study [26].

The only study that provided a sample size calculation based on a sexual function outcome was a non-RCT with two groups which provided an education-based intervention [30]. Although sample size was not achieved, the study found an improvement in sexual attitudes up to six weeks and sexual self-efficacy up to one week following the intervention [30]. Four studies reported results of single-group pre-post comparisons [10, 31, 33, 37–40]. One study (*n* = 31) involved PFM therapy (active + passive) + education, and reported improvements in all domains (dyspareunia – pain intensity and quality: NRS and MPQ; sexual function: FSFI; sexual matters: ICIQ-VS; sexual distress: FSDS-R; painful intercourse self-efficacy: PISES; frequency of sexual activities with vaginal penetration) following the intervention, with maintenance of effect 12-month post-intervention [10, 31, 33]; although the study was not powered for these outcomes. The other three studies provided an education-based intervention and presented mixed findings (FSFI, FSDS, DASA, etc.) [37–40], even though all studies had sexual function as a primary outcome but had no sample size calculation. However, all studies consistently found that education-based interventions alone did not improve sexual-related pain [37–40]. The study presenting the most intervention detail provided PFM therapy (active + passive) + education [10, 31, 33].

#### Psychological Function

Four RCTs provided data on psychological function [18, 26–28]. Only one RCT identified psychological distress (HADS) as the primary outcome which informed a sample size calculation (*n* = 318) [26], though no differences between groups were found in psychological (HADS) or symptom distress (MSAS-SF-R) following a predominantly education-based intervention involving mixed gynecological cancer types during radiotherapy [26]. Despite the lack of power calculation, with relatively small sample size and potentially low [28] or unclear [27] intervention dosage with unclear follow-up timepoints, two RCTs showed improvements in depression (*n* = 69) [27] and fears about cancer and sexuality (*n* = 32) [28] following education-based interventions. One recent RCT (*n* = 130) [18] showed improvements in mental states (HAMA, HAMD) following PFM therapy (active) + education, although no sample size calculation was provided and intervention reporting was limited in detail.

Four studies reported results of single-group pre-post comparisons [10, 33, 37–40]. One study (*n* = 31) involved PFM therapy (active + passive) + education, and showed improvements in all domains (body image concerns: BIS; pain anxiety: PASS; pain catastrophizing: PCS; depressive symptoms: BDI-II) following the intervention, which were maintained at 12-month post-intervention [10, 33], however the study was not powered for these outcomes. Three studies provided education-based interventions with mixed results [37–40]. Improvement in some domains of BSI-18 were seen in one study (*n* = 53), in which this was identified as a primary outcome measure [37, 38]. Improvement in depression (BDI) was seen in another study (*n* = 22), even though the primary outcome was sexual arousal [40], but no improvement in depression (BDI) was found following the education-based intervention in a study conducted by the same authors (*n* = 31) [39].

#### Quality of Life

Seven RCTs assessed quality of life as an outcome [19, 21, 23–27]. All except one study used a cancer-specific quality of life tool [21, 23–27], none were powered for quality of life outcomes, and the only study that listed quality of life as a primary outcome did not present between-group comparison results [23]. Overall, findings were mixed or equivocal, poorly reported, with no clear signal. One RCT (*n* = 226) investigated a combination of PFM therapy (active) + nursing education + yoga and showed an impact in favor of TG in all domains except physical function [21]. One RCT (*n* = 166) demonstrated the additional benefit of PFM therapy (active) to self-catheterization and detected a difference in burden (SPB) and comfort (GCB) in favor of PFM therapy (active) [19]. Two RCTs, with *n* = 88 [24] and *n* = 318 [26], investigated dilator therapy interventions and neither showed an impact on quality of life (EORTC QLQ-C30, FACT-G) [24, 26]. Two RCTs, with *n* = 100 [25] and *n* = 69 [27], provided education-based interventions and indicated improvements in some domains of quality of life (FACT-CX [FACT-G + CX]), QLICP-CE, EORTC QLQ-C30) [25, 27].

One single-group study assessed the effect of an education-based intervention (brief mindfulness-based CBT) on quality of life (SF-36) [40]. Results were mixed with an improvement in mental health but no improvement in physical health. This was based on a small sample (*n* = 22), no sample size calculation was conducted, and the primary outcome was sexual arousal [40].

#### PFM function

Four RCTs provided data on PFM function [20, 22–24], but only three studies reported between-group comparison results [20, 23, 24]. Two RCTs demonstrated an increase in PFM strength (digital palpation, vaginal squeeze pressure) following PFM therapy (active or electrostimulation) ± other (*n* = 28 and *n* = 91) [20, 23], although one RCT had significant differences on two out of four PFM function outcomes between groups at baseline which were not accounted for in analysis [20]. One RCT found no differences between groups (digital palpation with classification of participants into categories representing contractility/relaxation capacity levels) after PFM therapy (passive) (*n* = 88) [24]. One RCT (*n* = 88) found a reduced motor evoked potential threshold following sacral stimulation after PFM therapy (active) + education + other, which could be a marker for improved PFM function via peripheral neuromuscular function, among other measures which were not found different between groups [23]. No RCTs were powered for PFM function outcomes, and only one listed PFM function outcomes as the study primary outcome [23].

Four studies reported results of single-group pre-post comparisons [32, 34–36]. All studies provided PFM therapy (active) [32, 34–36], three had an additional education components [32, 34, 35], and one also provided PFM therapy (passive) [32]. Findings were mixed, and the direction of pre-post differences were inconsistent depending on the outcome measure or tool used; however, better results have been reported in the study on PFM therapy (active + passive) + education [32], and results suggest improvement in all PFM morphometry outcomes as assessed by ultrasound imaging, corresponding to improved PFM function at rest and during voluntary contraction, although dynamometric outcomes indicated improved PFM contractile properties other than for PFM strength [32]. Quality reporting of TG interventions was higher in single-group studies than in RCTs.

#### Evidence Summary

Table 6 presents the summary of the findings with level of evidence [17] and recommendations for each outcome.

**Table 6:** Summary of Findings with Level of Evidence and Recommendations

Outcome	Differences with previous review	Summary; level of evidence; recommendations
Bladder function	This review presents additional studies to add to the evidence, compared with the previous review [9]	**• PFM (active) + education therapies** ◦ They may or may not improve bladder function (inconsistent findings), however the largest of these studies that found a significant improvement between groups [18] (**level of evidence: 2**) is the best guide of a cautious recommendation for these therapies above usual care ◦ They may be beneficial for bladder function (**level of evidence: 4**) **• PFM (active and/or electrostimulation) therapies** ◦ They may improve PVR (**level of evidence: 2 and 3**), and they may be recommended above usual care. Caution is required regarding the use of electrostimulation on pelvic floor tissues soon after cancer treatment
Bowel function	This review presents additional studies to add to the evidence, compared with the previous review [9]	**• PFM (active) + education therapies** ◦ They may or may not improve bowel function (inconsistent findings), however the largest of these studies, which had a more intensive intervention and contrast with the comparator group, that found a significant improvement between groups [18] (**level of evidence: 2**) is the best guide of a cautious recommendation for these therapies ◦ They may be beneficial for bowel function (**level of evidence: 4**) **• PFM (active ± passive) + education therapies** ◦ They may be beneficial for bowel function (**level of evidence: 4**)
Vaginal function	This review presents evidence on vaginal function via several domains which were not included in the previous review [9]	**• PFM (active) + education therapies** ◦ They may improve pelvic organ prolapse (**level of evidence: 2**) **• PFM (passive) therapies** ◦ They may not be effective to improve vaginal dimensions or stenosis (**level of evidence: 2**) **• PFM (active + passive) + education therapies** ◦ They may be beneficial for vaginal function (**level of evidence: 4**) **• Education-based interventions** ◦ Thay may not provide benefits for vaginal function (**level of evidence: 4**)
Overall pelvic floor function	This review identified an overall pelvic floor function outcome from two recent studies which were not part of the previous review [9]	**• PFM (active) + education therapies** ◦ They may improve overall pelvic floor function (**level of evidence: 2**) **• PFM (active + electrostimulation) therapies** ◦ They may be beneficial for overall pelvic floor function (**level of evidence:** **3**)
Sexual function	This review presents additional studies to add to the evidence compared with the previous review [9]	**• PFM (active ± passive) + education therapies ± other** ◦ They may improve overall sexual function (**level of evidence: 2**) **• Education-based interventions** ◦ They may improve some aspects of sexual function (**level of evidence: 2 and 4**) ◦ They are unlikely to improve sexual-related pain (**level of evidence: 4**)
Psychological function	The previous review did not include psychological function outcomes [9]	**• PFM (active ± passive) + education therapies** ◦ They may improve psychological function (**level of evidence: 2 and 4**) **• Education-based interventions** ◦ They may or may not improve psychological function (inconsistent findings likely due to population heterogeneity) (**level of evidence: 2 and 4**)
Quality of life	This review presents additional studies to add to the evidence compared with the previous review [9]	**• PFM (active) + education therapies ± other** ◦ They may improve various domains of quality of life (**level of evidence: 2**) **• PFM (active) therapies** ◦ They may improve burden and comfort (**level of evidence: 2**) **• PFM (passive) therapies** ◦ They may not improve quality of life (**level of evidence: 2**) **• Education-based interventions** ◦ They may or may not improve quality of life (inconsistent findings) (**level of evidence: 2**), but they may improve some domains of quality of life (**level of evidence: 4**)
PFM function	This review included eight studies that reported outcomes of PFM function across a range of muscle properties, whereas the previous review included two studies which focused on PFM strength [9]	**• PFM (active or electrostimulation) therapies** ◦ They may improve PFM strength (**level of evidence: 2**) **• PFM (passive) therapies** ◦ They may not improve PFM strength (**level of evidence: 2**) **• PFM (active ± passive) + education therapies** ◦ They may improve overall PFM function (**level of evidence: 2 and 4**)

PFM: pelvic floor muscle, PVR: post-void residual

Oxford Centre for Evidence-Based Medicine (OCEBM) 2011 Levels of Evidence framework [17]

Level 1: ‘Systematic review of randomized trials or n-of-1 trials’, Level 2: ‘Randomized trial or observational study with dramatic effect’, Level 3: ‘Non-randomized controlled cohort/follow-up study’, Level 4: ‘Case-series, case–control studies, or historically controlled studies’

## Discussion

### Summary

This systematic review has summarized the evidence for the effectiveness of conservative therapies, namely PFM and education-based therapies, on bladder, bowel, vaginal, sexual, psychological function, quality of life, and PFM function in gynecological cancer populations. Overall, findings from RCTs and non-RCTs suggest that combined (multimodal) therapies – combinations of PFM (active > passive) + education – appear more effective than any other conservative therapies (i.e., PFM therapies or education alone) for vaginal, overall pelvic floor, sexual, and PFM function. For bladder function, most investigated interventions were PFM therapies (active and/or electrostimulation) only, and findings suggest that these therapies can improve bladder function; however, it is unclear if an education component may provide additional benefits. Evidence was limited but findings were encouraging for PFM (active) + education therapies to improve bowel function. The lack of improvement seen in RCTs that investigated the effect of PFM (passive: dilators) or education-based therapies to improve vaginal stenosis or vaginal dimensions, contrasted with the improvement seen in a single-group study that provided both PFM therapy (active + passive) + education, suggesting that a well-supervised multimodal therapies may be more effective for vaginal outcomes. Studies that investigated sexual function also suggested a better effect with combined therapies. The lack of improvement in sexual pain following education alone – if the pain experienced by the participants in these studies was chronic – is in line with the literature indicating that education alone is not as effective as pain science education and exercises [41], aligning with the findings of Cyr et al. [10, 31, 33]. Conservative therapies may improve psychological function, although available data do not appear to favor a particular therapy. A common observation that emerged from interpretation of findings for all outcomes, except for quality of life, is that intervention dosage, adherence, and supervision appear critical for optimal effectiveness. For quality of life, findings were mixed, and no clear conclusions can be drawn on the effectiveness of conservative therapies for this particular outcome. Even though adverse effects were not systematically reported in studies, very few were noted.

Interpretation of these findings must consider the limited number of included studies for each outcome (bladder function > sexual function > psychological function = quality of life > PFM function > bowel function = vaginal function > overall pelvic floor function). Additionally, the majority of studies either were non-powered and did not provide sample size calculations, had small sample sizes, did not specify a primary outcome or end timepoint, or provided poor description of TG interventions. Despite these methodological limitations, there are positive signals for certain outcomes and for specific therapies as summarized above. Findings from several studies in this review may be used to build our understanding of feasibility of recruitment and delivery of conservative therapies, to help inform power calculations for future trials, and to provide some evidence for clinical practice. The breadth of this systematic review also allowed the identification of areas for research which remain underexplored.

### General Observations and Recommendations for Future Research

Several studies in this review found no between- or within-group differences for some specific outcomes related to bladder [22, 23, 29, 34, 36], bowel [23, 34, 36], vaginal [24, 26, 39, 40], sexual [26, 28, 30, 37–40], psychological function [26, 37–39], quality of life [21, 24–26, 40], and PFM function [23, 24, 32, 34–36]. This is likely explained by one or a combination of factors which are presented in Box 1.

## Box 1: Factors That May Explain Non-Significant Between- or Within-Group Differences.

**Table:** 

Factors
(a) The study lacks sufficient power to detect a statistically significant difference for this particular outcome (type II error)
(b) There are multiple comparisons without adjustment
(c) There are close similarities between TG intervention and CG intervention which could contribute to similar rather than different effect between groups
(d) The dosage of the TG intervention is insufficient to change the outcome (e.g., insufficient intensity, short duration, or low supervision)
(e) The TG intervention is unlikely to change outcome because of a mismatch between the intervention and the outcome (e.g., weak rationale for a short-duration physical-based intervention intended to improve quality of life outcome)
(f) There is high outcome measure variance at baseline (i.e., heterogeneity in either between- or within-groups) which could contribute to variation in participant treatment response
(g) Baseline measures suggest risk of floor/ceiling effect, making it challenging to detect a statistically significant difference, particularly when the baseline outcome indicates low severity or impact
(h) Properties (e.g., reliability, validity, and sensitivity to change) of the outcome measure or tool used to detect a statistically significant difference in the outcome are unknown or not robust

CG comparator group, TG treatment group

In this review, factors (a) and (b) emerged as the most problematic issue across studies showing no significant differences, emphasizing the necessity for future research to ensure adequate sample size to enhance confidence in the obtained results. A low proportion of studies identified a primary outcome, while a high proportion included more than three types of outcomes. This may contribute to a misunderstanding of the ultimate goal of the therapy. Although the inclusion of multiple outcomes may offer valuable clinical insights into potential mechanisms of treatment, additional benefits, or interactions among outcomes, each additional outcome must be sufficiently powered if it is to provide evidence of effectiveness, and bias associated with multiple comparisons should be considered. Future studies should choose a primary outcome based on the intent of the study and intervention: e.g., to establish clinical effectiveness based on an outcome important to the patient and likely to change in response to the intervention; or if a mechanistic study, an outcome that can assess the mechanism(s) of action of the intervention, and the cause(s) of differential responses. It is worth noting that studies that assessed a PFM function outcome used a range of outcome measures and tools, such as digital palpation, manometry, assessment using magnetic stimulation, ultrasound imaging, dynamometry, and EMG. These do not all provide the same information about PFM function, and findings should be interpreted accordingly.

Factors (c), (d), and (e) highlight pitfalls related to the TG intervention. Disappointingly, the quality reporting of TG interventions does not appear to have improved after the publication of the TIDieR checklist in 2014 [13], and slightly more non-RCTs (*n* = 4/9, 44%) compared with RCTs (*n* = 3/11, 27%) described their TG intervention in more detail, satisfying at least 10 items out of 12. Given that this low-quality reporting makes replication difficult in research and implementation for clinical practice, this underscores the importance of future studies to adequately define TG and CG interventions, with rationale for the targeted outcome and population, with supporting evidence. Regarding factors (d) and (e), effects in some outcomes (e.g., psychological function, quality of life, and PFM function) could have been limited because some interventions were not designed to directly target these outcomes and involved potentially inadequate dosage to observe improvements. Further studies that are powered and specifically targeted for these outcomes with adequate dosage and information on participant characteristics are required to inform intervention effectiveness.

Factors (f) and (g) can be linked to selection bias, where a more homogeneous group of participants with the outcome of interest may increase the likelihood of detecting a statistically significant difference following intervention; however, the homogeneity may increase the difficulty with recruitment. As for factor (h), it is critical that future studies employ reliable, valid, and sensitive-to-change outcome measures or tools to assess the effectiveness of conservative therapies.

In addition to the above recommendations for more robust study designs, there is a clear need for more investigation into conservative therapies across various gynecological cancer populations, particularly beyond cervical cancer populations. In addition, the potential benefits of interventions administered before and during cancer treatment are under-researched, with few studies including interventions delivered at this time. The long-term effectiveness of conservative therapies also remains insufficiently explored.

### General Recommendations for Clinical Practice

High-quality evidence forms the foundation of clinical practice, which has traditionally been obtained through RCTs [42]. However, RCTs are expensive and require substantial resources. Recruiting patients diagnosed with or treated for gynecological cancer is challenging [31, 43, 44], making it difficult to gather large samples in a timely manner for a single RCT. More collaboration between multiple centers and institutions is recommended to mobilize the necessary resources in this field of research. RCTs also typically focus on more established interventions, which can be problematic in fields where data supporting effective interventions is scarce. Nevertheless, clinical practice should be informed by the best level of evidence available, and the findings from non-RCTs in this review help inform clinical practice where RCT level evidence is unavailable [45].

Given the high prevalence of symptoms of pelvic floor disorders and sexual dysfunction in gynecological cancer populations and the evidence available supporting conservative therapies as generally safe and potentially effective, greater efforts should be made to integrate conservative therapies into screening, referral, and treatment pathways of care throughout the cancer trajectory. Caution is required regarding the use of electrostimulation on pelvic floor tissues given the lack of data supporting its safety in cancer populations; patients should be monitored closely for any adverse events that may arise when this or any other type of conservative therapies are provided. As the clinical context varies in different countries and across different regions (urban, rural, and remote), clinicians need to consider the most effective care they can deliver with available resources and to meet patient preferences, with consideration of in-person, remote, or hybrid models of care as required.

### Strengths and Limitations

This systematic review has several strengths. We conducted a comprehensive review, incorporating both RCT and non-RCT study designs, alongside a diverse array of outcomes, covering the spectrum of prehabilitation to rehabilitation conservative therapies in gynecological cancer populations. This approach allowed us to evaluate various levels of evidence to inform further research and provide essential clinical insights. A broad search was performed using a strategy adapted to the purpose of this review. Two reviewers independently conducted study selection, assessed risk of bias and quality assessment, and extracted data, with strong agreement. The TIDieR checklist was used to evaluate intervention reporting in studies [13].

This systematic review possesses several limitations that should be considered when interpreting the findings. In this review, we noted that the heterogeneity of interventions tested per outcome, the diversity of outcome measures used per outcome, and the quantity of appropriate post-intervention data to combine were insufficient to perform meta-analyses. Although this review included a large range of outcomes important to patients, the social domain individually was not covered, although it may be a dimension of quality of life. Given the significance of addressing diverse needs within cancer populations, inclusion of the social domain specifically may be considered in future reviews [46, 47]. In addition, the breadth of this work, including its conclusions, generalizations, and potential applications, is informed by the characteristics and quality of studies included. As the majority of studies represented females with cervical cancer and examined only short-term effectiveness of conservative therapies delivered after cancer treatment, findings of this systematic review may be generalizable to this specific population, this follow-up time frame, and timing of delivery.

## Conclusions

This is the first study to review the evidence on effectiveness of both PFM and education-based therapies in gynecological cancer populations, with some positive findings emerging of the benefit of these therapies on a range of important outcomes in this population. The findings of this systematic review should be interpreted with caution due to variation in quality of study reporting, as evidenced by the TIDieR checklist, and potential biases, informed by the risk of bias and quality assessment tools.

## **Key References**

- Brennen R, Lin KY, Denehy L, Frawley HC. The effect of pelvic floor muscle interventions on pelvic floor dysfunction after gynecological cancer treatment: a systematic review. Phys Ther. 2020.
  - This paper was the first systematic review on this topic and informed the search strategy of the current review.
- Campbell M, McKenzie JE, Sowden A, Katikireddi SV, Brennan SE, Ellis S, et al. Synthesis without meta-analysis (SWiM) in systematic reviews: reporting guideline. BMJ. 2020;368:l6890.
  - This document provides clear guidelines for synthesizing evidence narratively, enabling systematic and transparent reporting of findings and ensuring rigor and reliability of conclusions.
- Hoffmann TC, Glasziou PP, Boutron I, Milne R, Perera R, Moher D, et al. Better reporting of interventions: template for intervention description and replication (TIDieR) checklist and guide. BMJ. 2014;348:g1687.
  - This document provides a clear list of items that should be met for comprehensive and transparent reporting of interventions, facilitating replication and implementation of research findings. This process is critical for translating findings into practice.
- OCEBM Levels of Evidence Working Group. The Oxford 2011 Levels of Evidence. Oxford Centre for Evidence-Based Medicine. 2011.
  - This document provides a standardized framework for assessing the quality and strength of evidence across different studies, which ensures a consistent and objective evaluation of research findings, essential for drawing reliable and valid conclusions.

## Supplementary Information

Below is the link to the electronic supplementary material.

Supplementary file1 (DOCX 32 KB)

Supplementary file2 (DOCX 149 KB)

Supplementary file3 (DOCX 222 KB)

## Data Availability

No datasets were generated or analysed during the current study.
