# Supplementary material for: Effectiveness of Pelvic Floor Muscle and Education-Based Therapies on Bladder, Bowel, Vaginal, Sexual, Psychological Function, Quality of Life, and Pelvic Floor Muscle Function in Females Treated for Gynecological Cancer: A Systematic Review
Source: Curr Oncol Rep. 2024 Aug 23;26(11):1293–320. doi: 10.1007/s11912-024-01586-7 (PMC11579103; doi:10.1007/s11912-024-01586-7)
Supplement: Supplementary file 1 — Supplementary file1 (DOCX 32 KB) [file 11912_2024_1586_MOESM1_ESM.docx]

**Supplementary Information 1. Database Search Strategy.**

Studies included in the previous review [9] were considered for inclusion, and a search update using the same search strategy was performed to identify newly published studies since June 2018. Given that the previous review included studies written in English only [9], a supplementary search was conducted to identify studies written in French, and none were found. One additional and separate search was performed to identify studies on education-based therapies in gynecological cancer populations. No restrictions on publication date were applied. References cited in included studies were considered for inclusion. For screening, if a report was unretrievable, attempts were made to contact the authors to obtain the report.

| **Database search strategy and related question** | **Database** | **Search terms for populations** | **Search terms for interventions** | **Other search terms** |
| --- | --- | --- | --- | --- |
| 1. What is the evidence of the effectiveness of non-surgical, non-pharmaceutical, pelvic floor muscle (PFM) therapies on any type of pelvic floor dysfunction in patients before, during, or after any type of treatment for any type of gynecological cancer? | CINAHL | (“gyn#ecol*” OR “cervi*” OR “uter*” OR “endomet*” OR “vulv*” OR “vagin*” OR “ovar*”) AND (“cancer” OR “neoplasm”) | “physiotherap*” OR “therap*” OR “*training” OR “exercise OR educat*” OR “dilator” OR “physical therap*” OR “rehabilitat*” OR “biofeedback” OR “conservative therapy” OR “behavio#ral therap*” OR “Kegel” | “pelvic floor” OR “pelvic floor muscle” |
|  | Cochrane Library | (“gynecol*” OR “cervi*” OR “uter*” OR “endomet*” OR “vulv*” OR “vagin*” OR “ovar*”) AND (“cancer” OR “neoplasm”) | “physiotherap*” OR “therap*” OR “*training” OR “exercise OR educat*” OR “dilator” OR “physical therap*” OR “rehabilitat*” OR “biofeedback” OR “conservative therapy” OR “behavioural therap*” OR “Kegel” | “pelvic floor” OR “pelvic floor muscle” |
|  | OVID Medline / PsycINFO / Embase / Emcare | (“gyn?ecol*” OR “cervi*” OR “uter*” OR “endomet*” OR “vulv*” OR “vagin*” OR “ovar*”) AND (“cancer” OR “neoplasm”) | “physiotherap*” OR “therap*” OR “*training” OR “exercise OR educat*” OR “dilator” OR “physical therap*” OR “rehabilitat*” OR “biofeedback” OR “conservative therapy” OR “behavio?ral therap*” OR “Kegel” | “pelvic floor” OR “pelvic floor muscle” |
| 2. What is the evidence of the effectiveness of education-based therapies on any type of pelvic floor dysfunction in patients before, during, or after any type of treatment for any type of gynecological cancer? | CINAHL | (“gyn#ecol*” OR “cervi*” OR “uter*” OR “endomet*” OR “vulv*” OR “vagin*” OR “ovar*”) AND (“cancer” OR “neoplasm”) | ((“pain” OR “sexual” OR “psychological” OR “therapeutic” OR “psychosexual” OR “neuroscience” OR “patient”) AND “education”) OR (“behavio*” AND (“therapy” OR “intervention”)) OR “CBT” OR (“sex*” AND (“therapy” OR “rehabilitation”)) OR “mindfulness” | “pelvic floor” OR “pelvic floor muscle”  AND  “urinary incontinence” OR “fecal incontinence” OR “anal incontinence” OR (“urinary” AND “urge*”) OR “genitourinary syndrome of menopause” OR “sexual dysfunction” OR “dyspareunia” OR “vaginismus” OR “pelvic organ prolapse”  AND  (“intervention*” AND “study”) OR “clinical trial” OR “pilot study” OR “randomized controlled trial” OR “non-randomized controlled trial” OR “pre-post study” OR “quasi-experimental study” OR “experimental study”) |
|  | Cochrane Library | (“gynecol*” OR “cervi*” OR “uter*” OR “endomet*” OR “vulv*” OR “vagin*” OR “ovar*”) AND (“cancer” OR “neoplasm”) | ((“pain” OR “sexual” OR “psychological” OR “therapeutic” OR “psychosexual” OR “neuroscience” OR “patient”) AND “education”) OR (“behavio*” AND (“therapy” OR “intervention”)) OR “CBT” OR (“sex*” AND (“therapy” OR “rehabilitation”)) OR “mindfulness” | “pelvic floor” OR “pelvic floor muscle”  AND  “urinary incontinence” OR “fecal incontinence” OR “anal incontinence” OR (“urinary” AND “urge*”) OR “genitourinary syndrome of menopause” OR “sexual dysfunction” OR “dyspareunia” OR “vaginismus” OR “pelvic organ prolapse”  AND  (“intervention*” AND “study”) OR “clinical trial” OR “pilot study” OR “randomized controlled trial” OR “non-randomized controlled trial” OR “pre-post study” OR “quasi-experimental study” OR “experimental study”) |
|  | OVID Medline / PsycINFO / Embase / Emcare | (“gyn?ecol*” OR “cervi*” OR “uter*” OR “endomet*” OR “vulv*” OR “vagin*” OR “ovar*”) AND (“cancer” OR “neoplasm”) | ((“pain” OR “sexual” OR “psychological” OR “therapeutic” OR “psychosexual” OR “neuroscience” OR “patient”) AND “education”) OR (“behavio*” AND (“therapy” OR “intervention”)) OR “CBT” OR (“sex*” AND (“therapy” OR “rehabilitation”)) OR “mindfulness” | “pelvic floor” OR “pelvic floor muscle”  AND  “urinary incontinence” OR “fecal incontinence” OR “anal incontinence” OR (“urinary” AND “urge*”) OR “genitourinary syndrome of menopause” OR “sexual dysfunction” OR “dyspareunia” OR “vaginismus” OR “pelvic organ prolapse”  AND  (“intervention*” AND “study”) OR “clinical trial” OR “pilot study” OR “randomized controlled trial” OR “non-randomized controlled trial” OR “pre-post study” OR “quasi-experimental study” OR “experimental study”) |
