# Supplementary material for: Effectiveness of Pelvic Floor Muscle and Education-Based Therapies on Bladder, Bowel, Vaginal, Sexual, Psychological Function, Quality of Life, and Pelvic Floor Muscle Function in Females Treated for Gynecological Cancer: A Systematic Review
Source: Curr Oncol Rep. 2024 Aug 23;26(11):1293–320. doi: 10.1007/s11912-024-01586-7 (PMC11579103; doi:10.1007/s11912-024-01586-7)
Supplement: Supplementary file 2 — Supplementary file2 (DOCX 149 KB) [file 11912_2024_1586_MOESM2_ESM.docx]

**Supplementary Information 2. Treatment Group Interventions According to the TIDieR Checklist and as Reported by the Authors.**

(a) Summary TIDieR Checklist

| **Authors, year** | **Study design** | **Name of intervention** | **Rationale** | **Materials** | **Procedures** | **Provider** | **Mode of delivery** | **Location** | **Intervention dosage** | **Tailoring** | **Modifications** | **Planned intervention adherence & strategies for adherence** | **Actual intervention adherence** | **TIDieR score** |
| --- | --- | --- | --- | --- | --- | --- | --- | --- | --- | --- | --- | --- | --- | --- |
| Sun et al., 2023 | RCT | 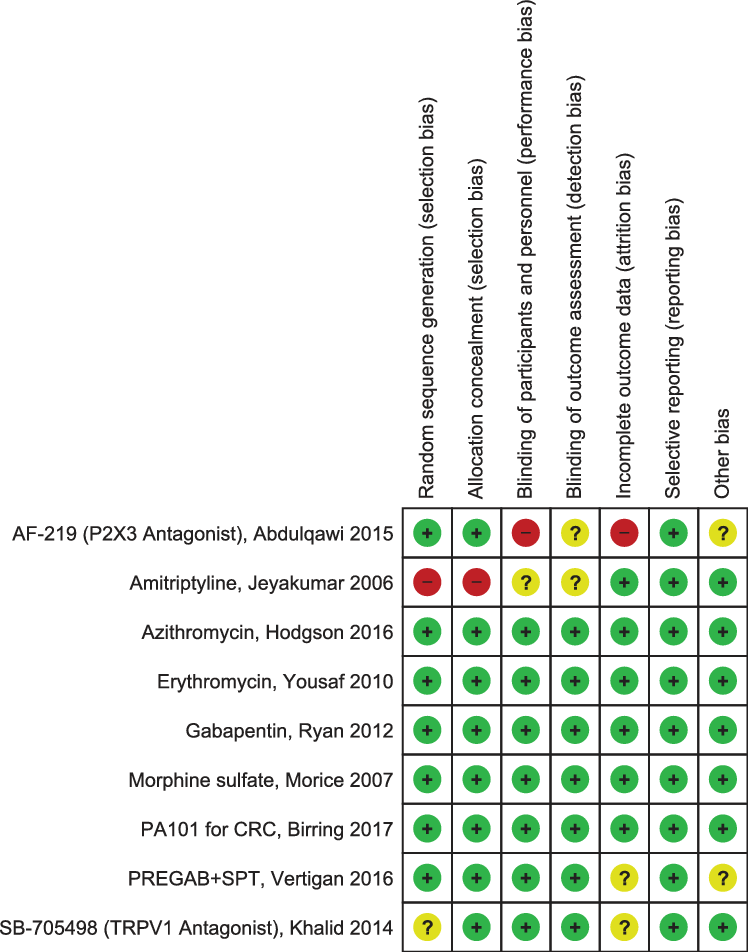 | 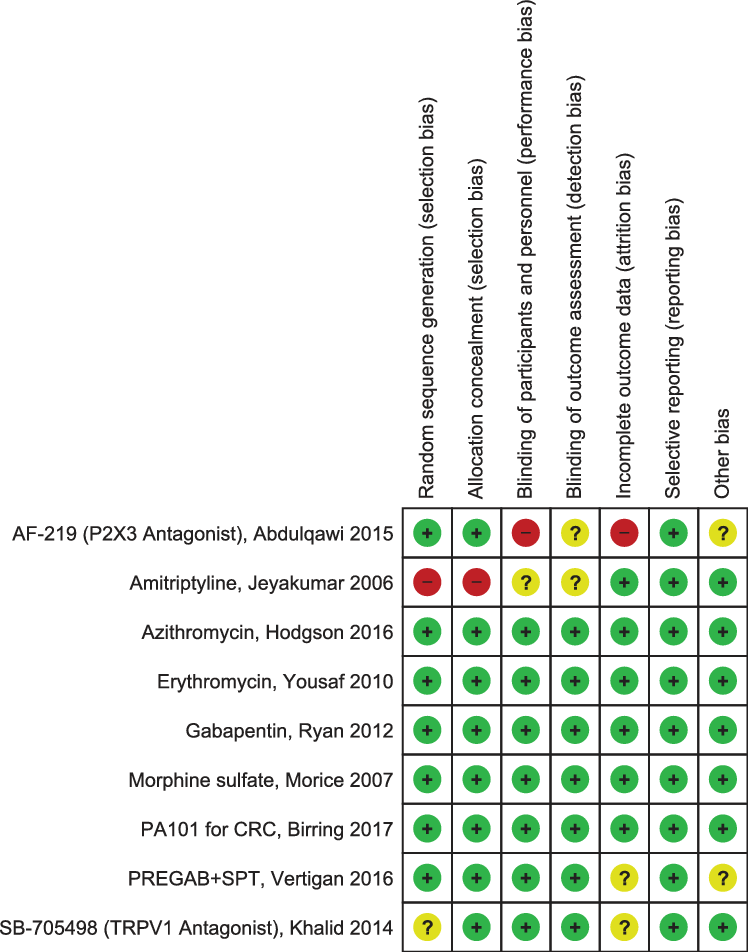 | 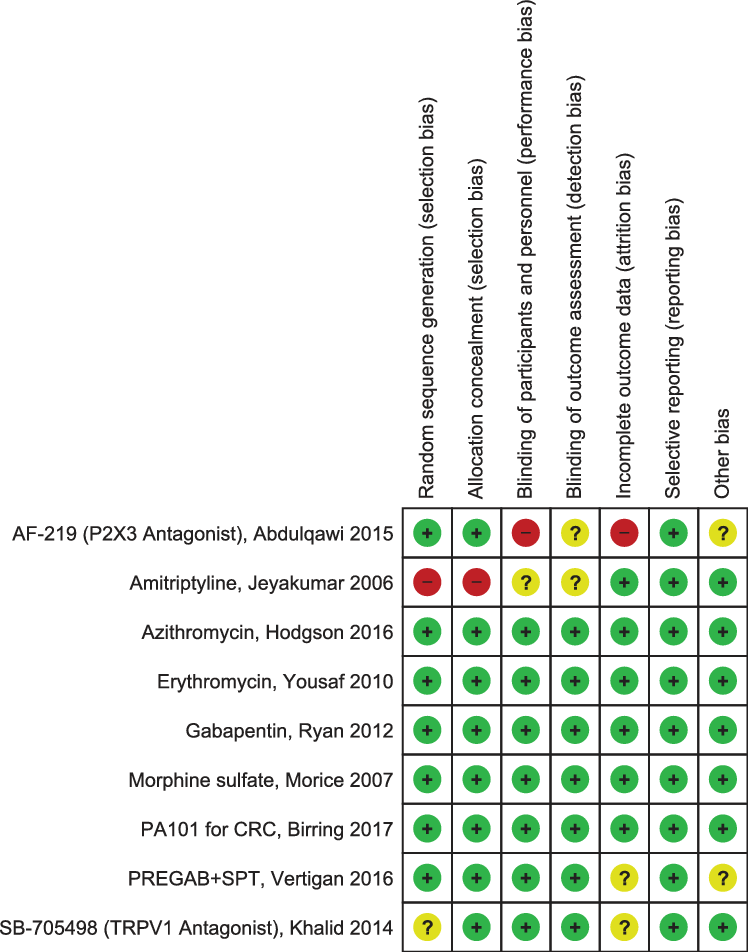 | 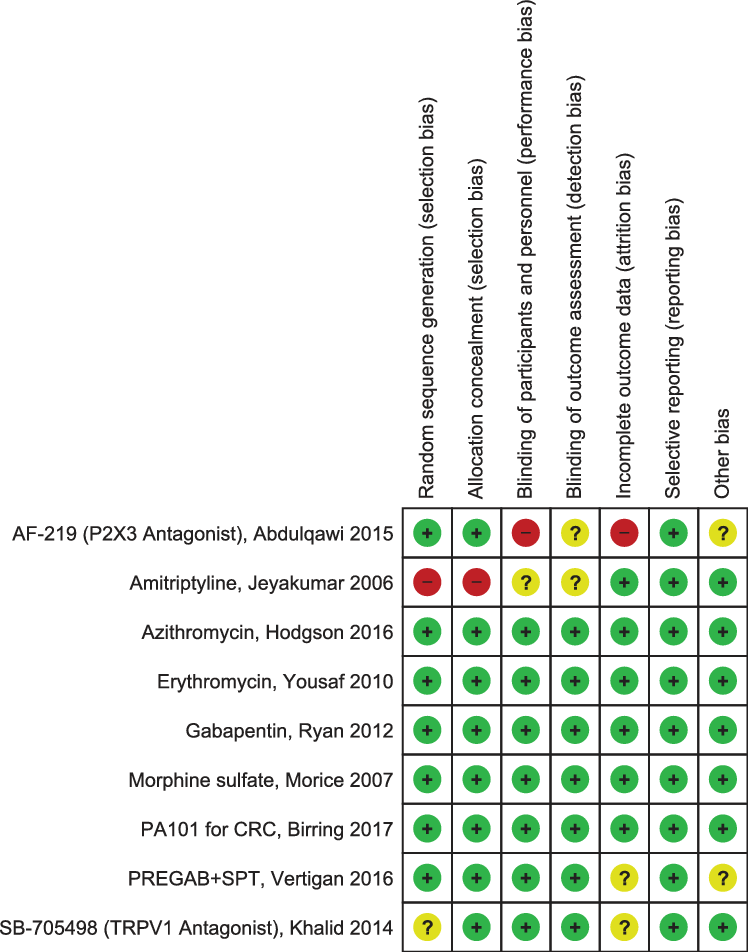 | 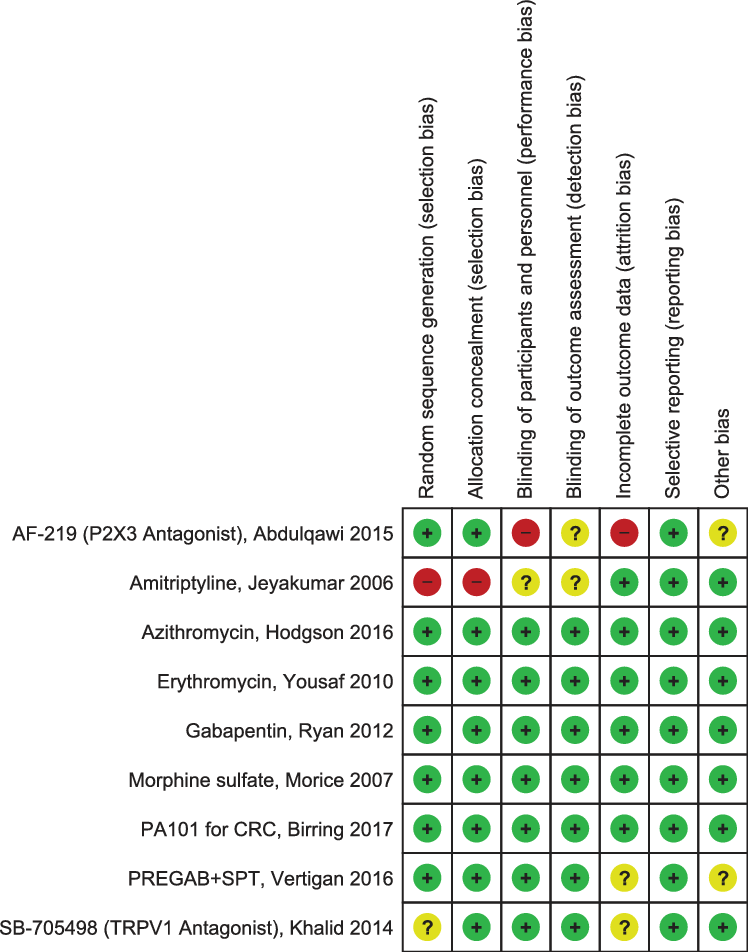 | 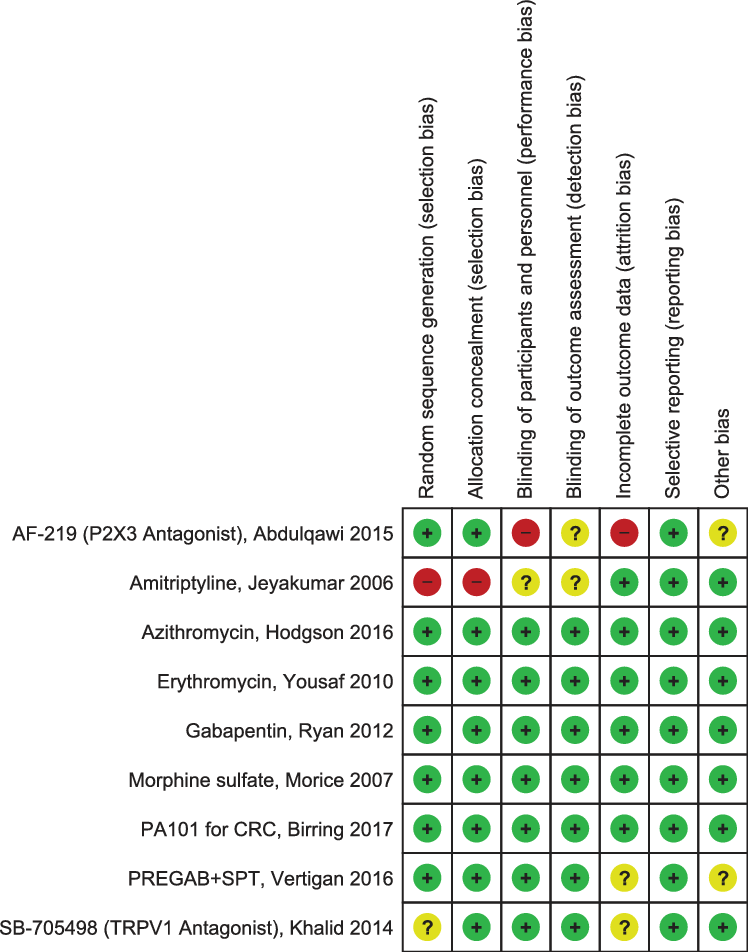 | 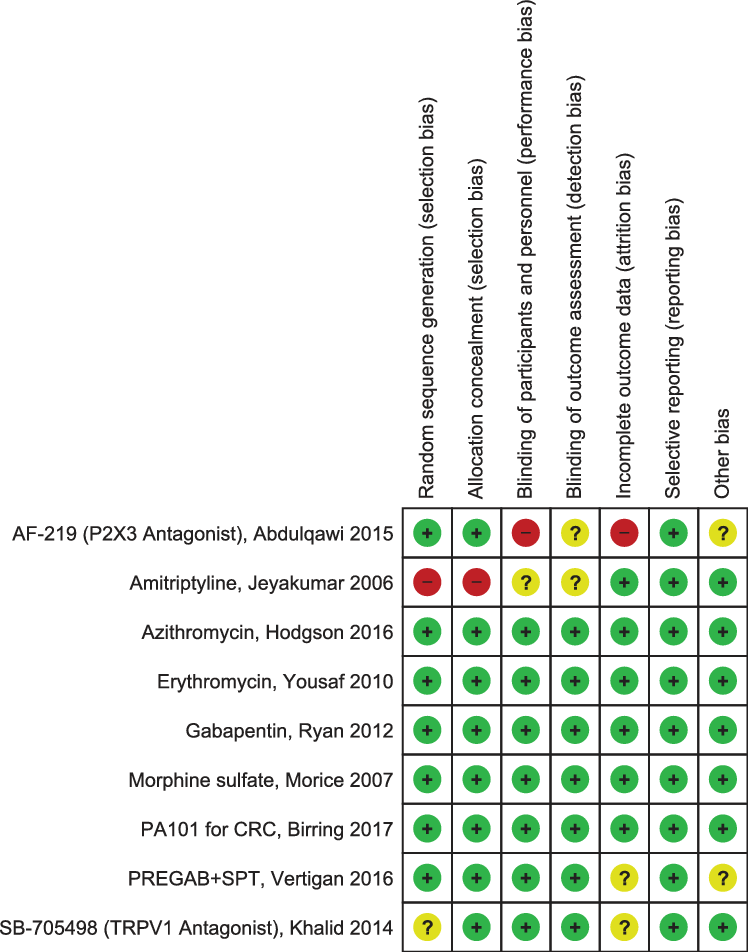 | 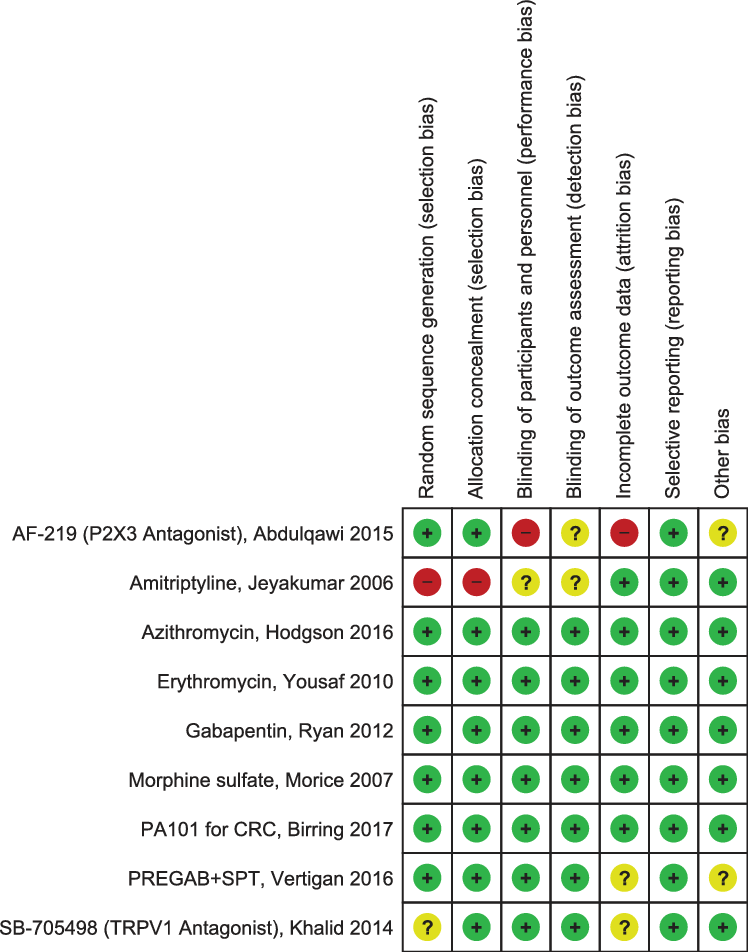 | 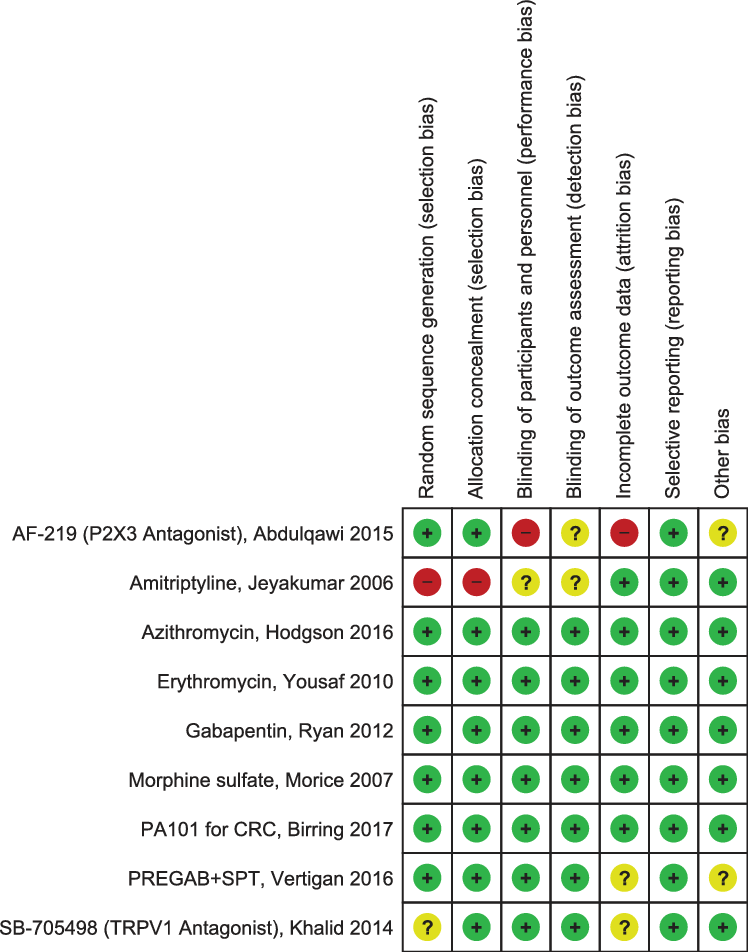 | 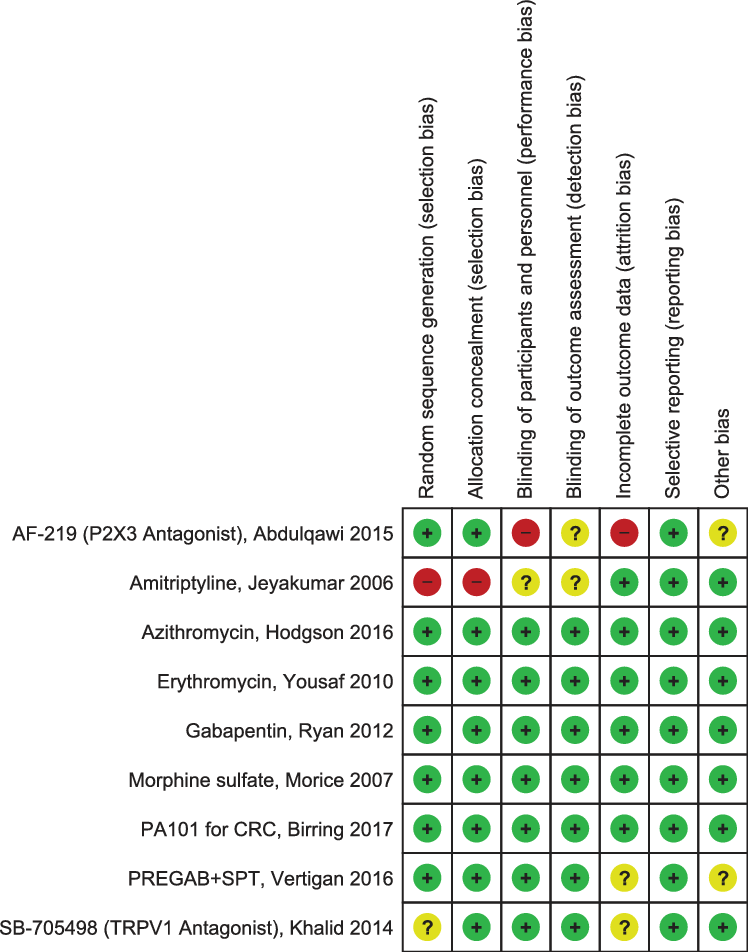 | 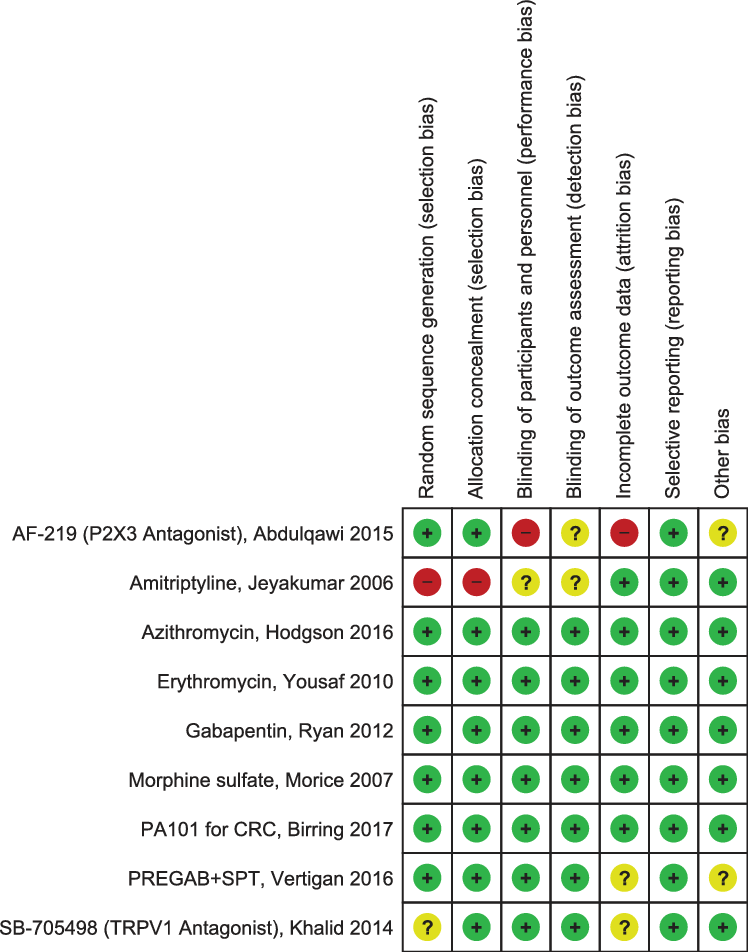 | 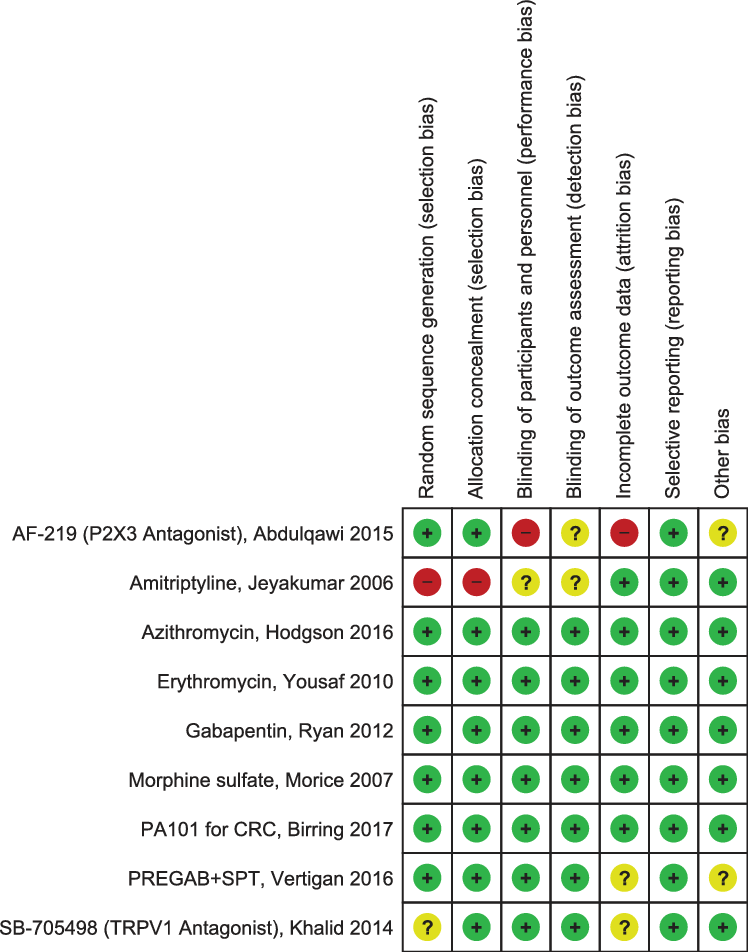 | 7 |
| Zong et al., 2022 | RCT | 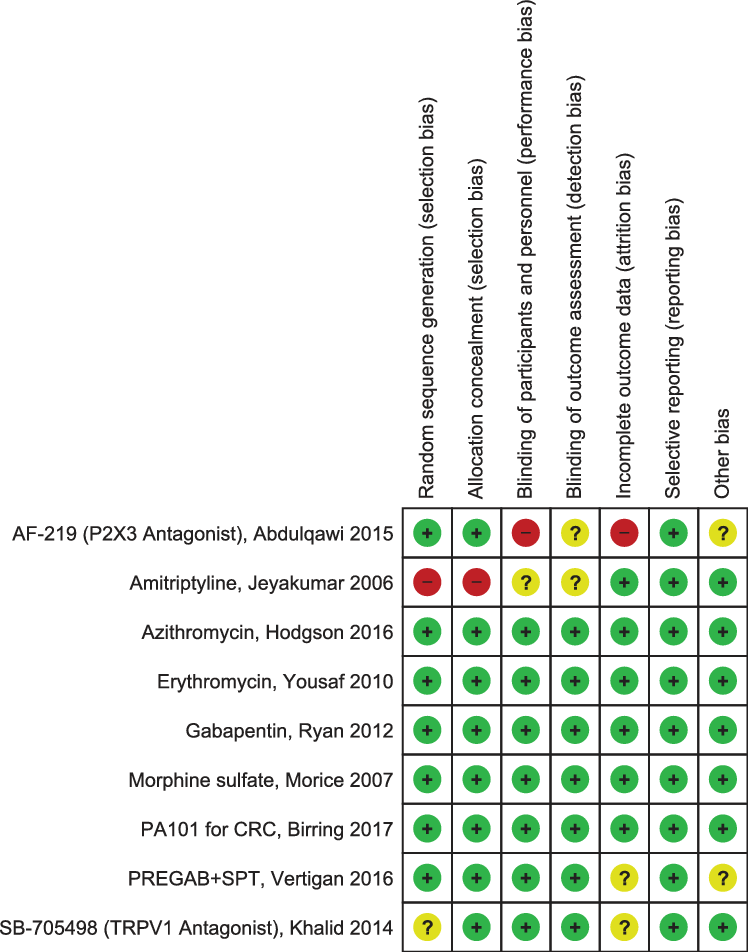 | 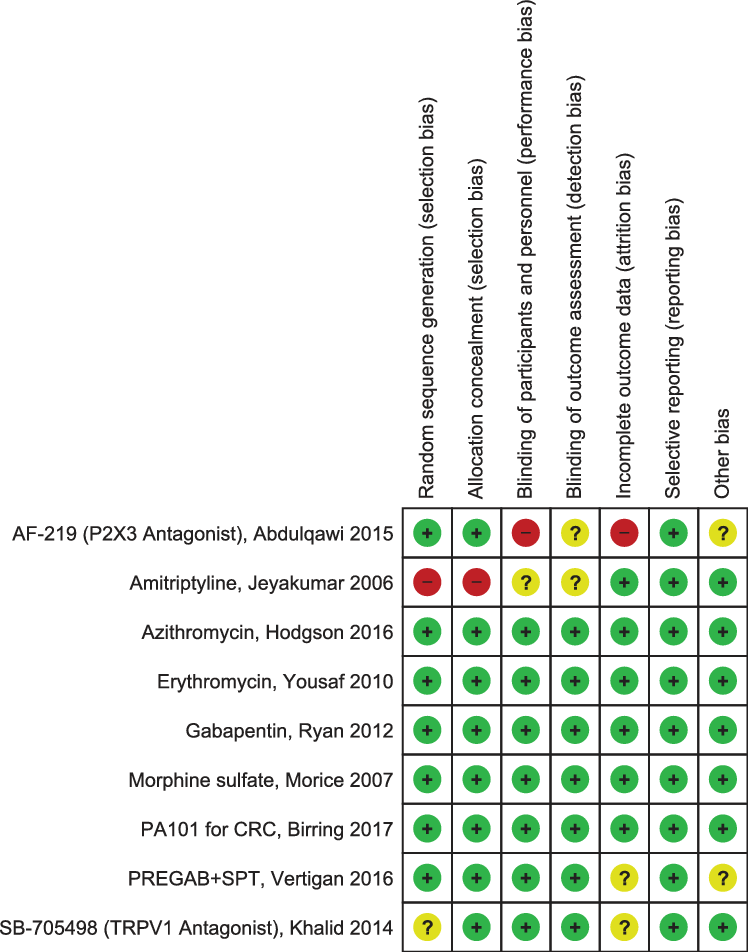 | 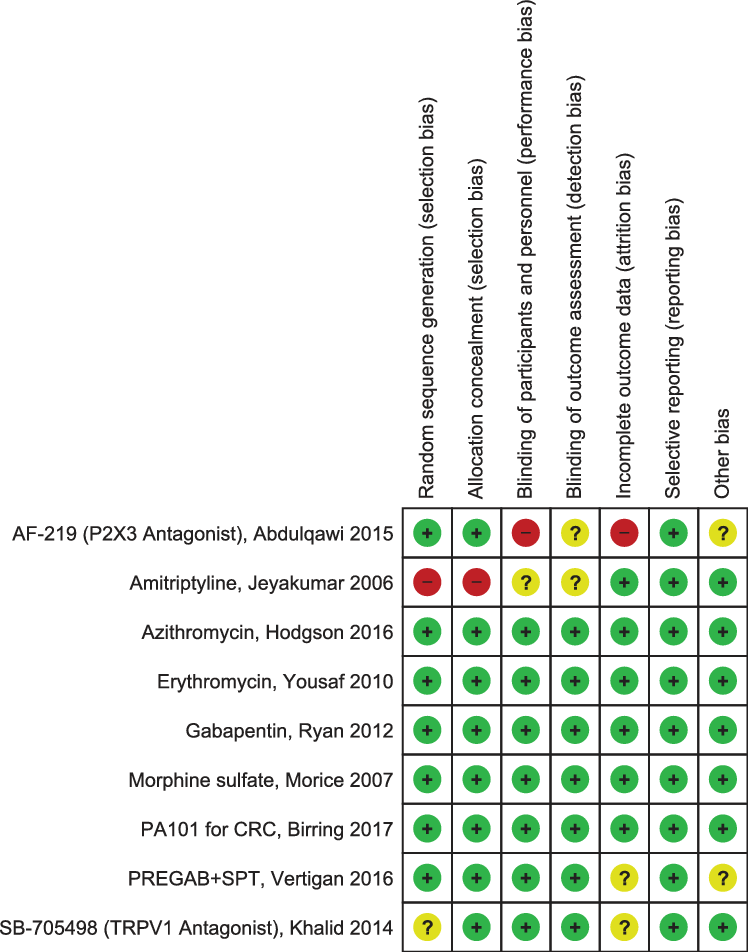 | 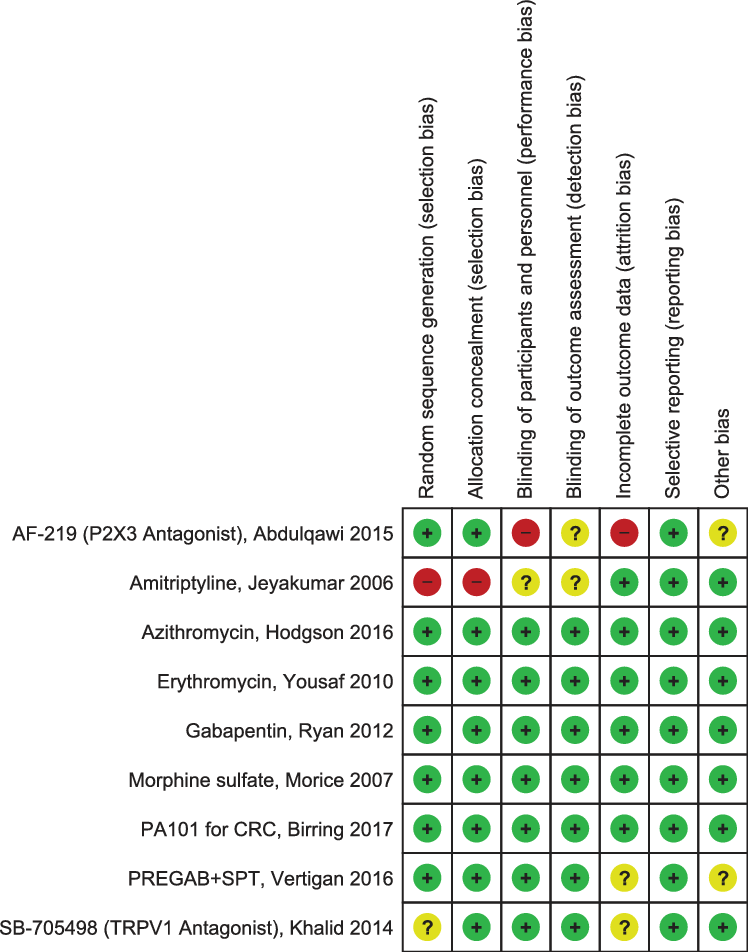 | 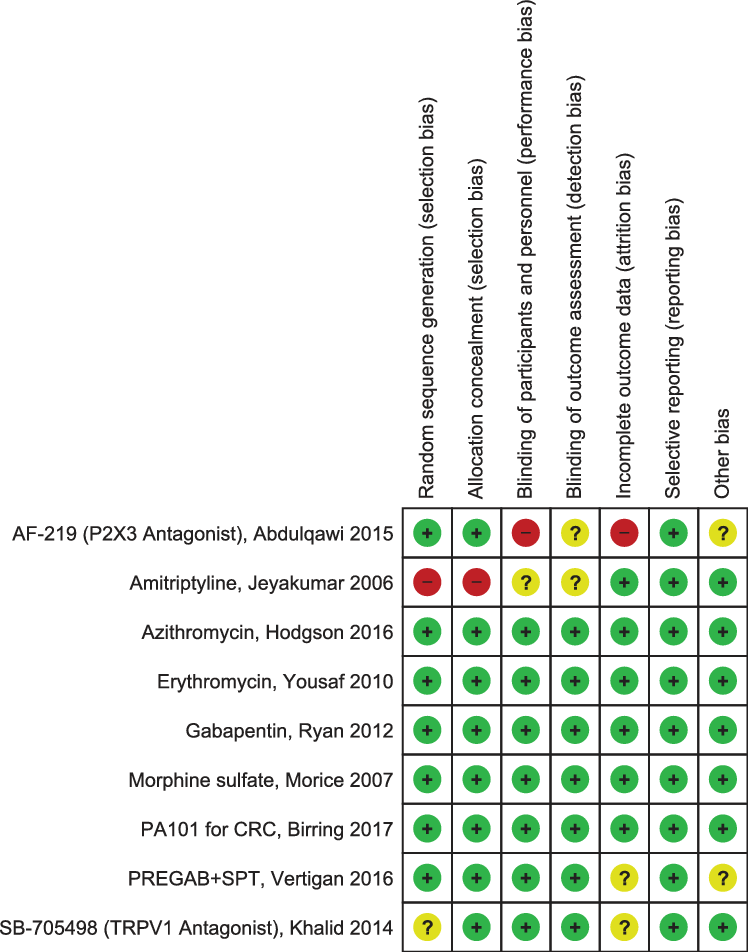 | 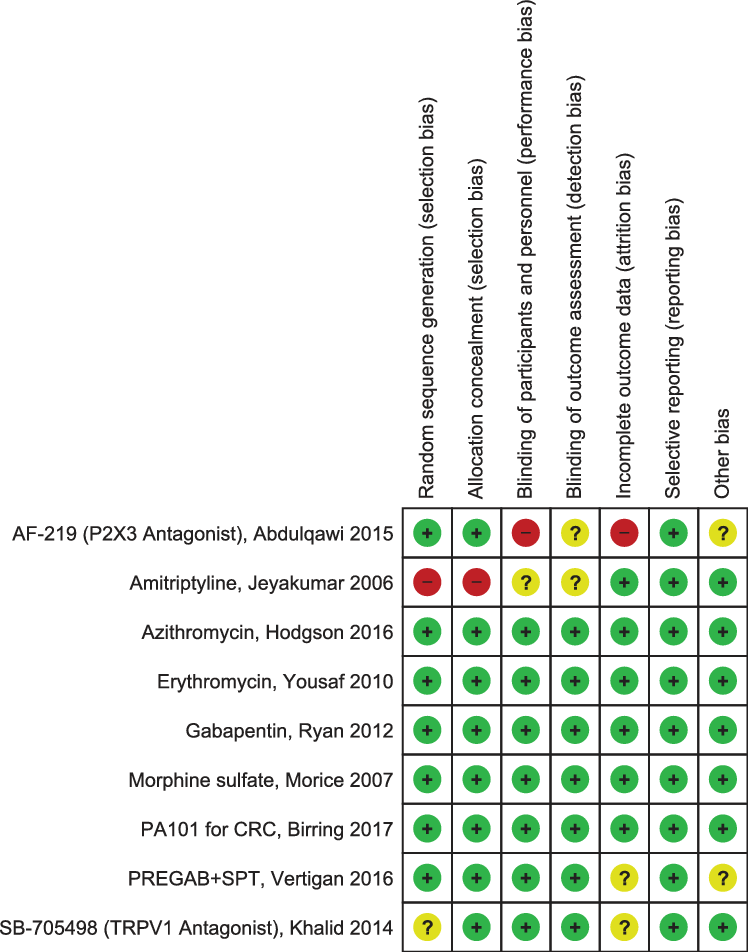 | 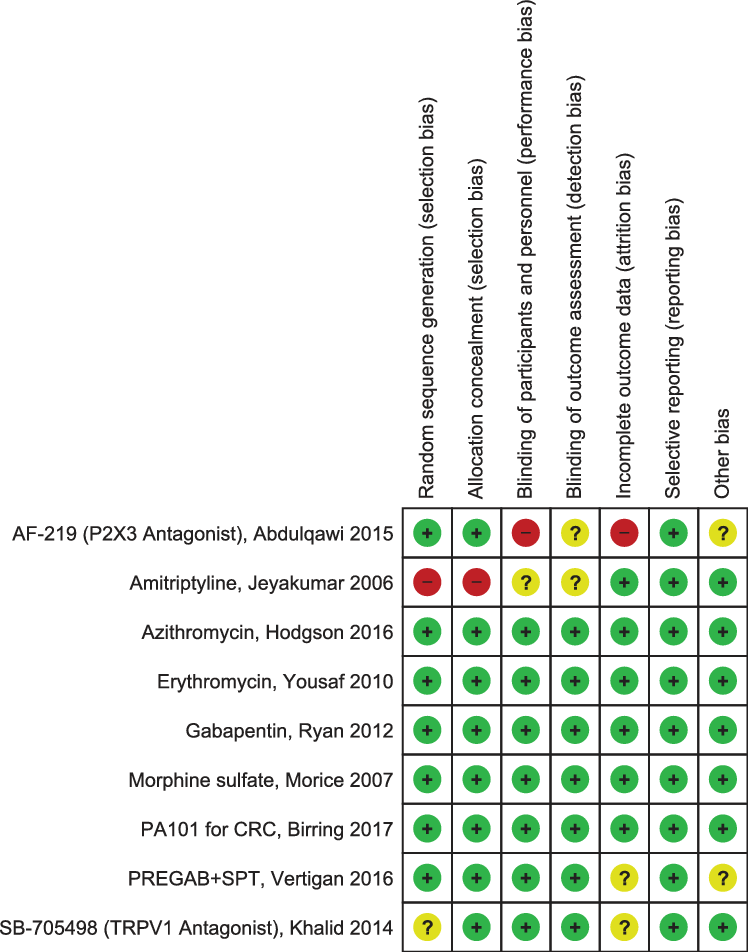 | 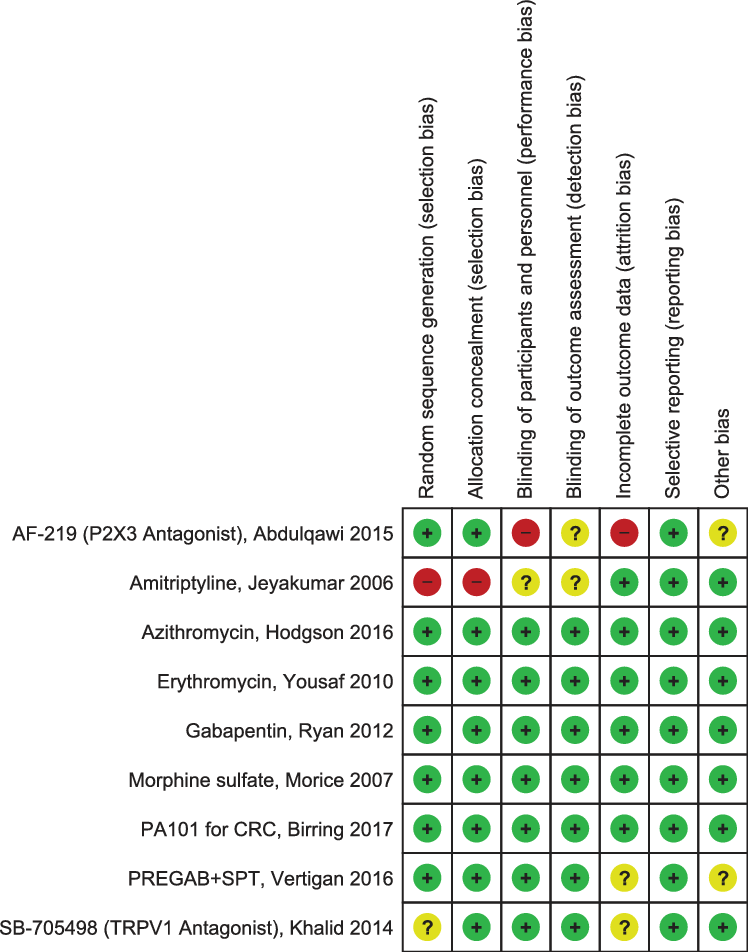 | 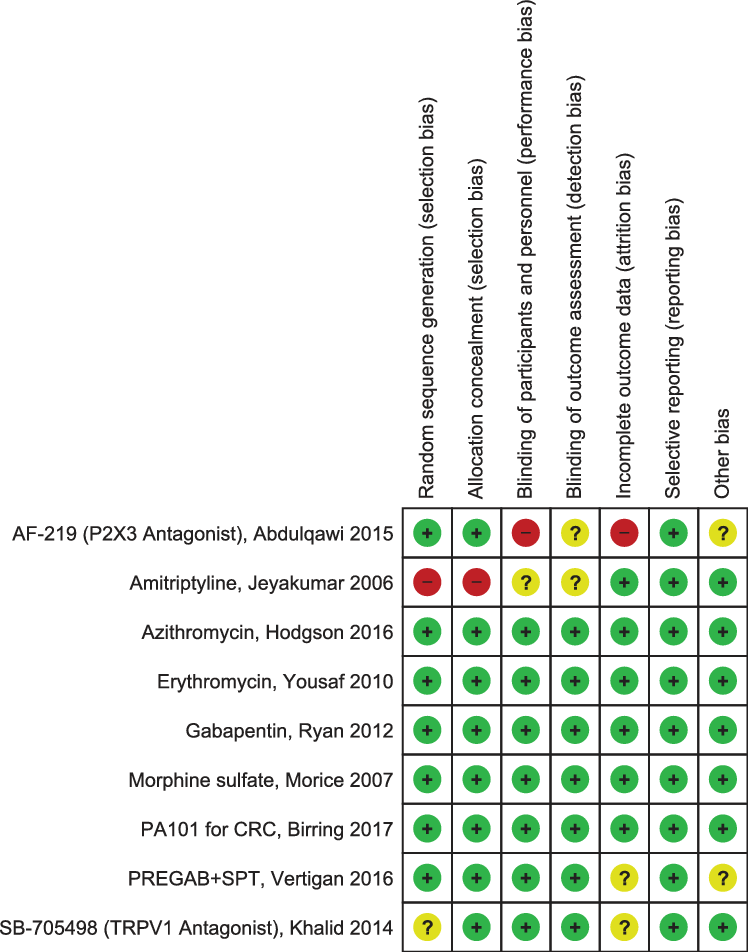 | 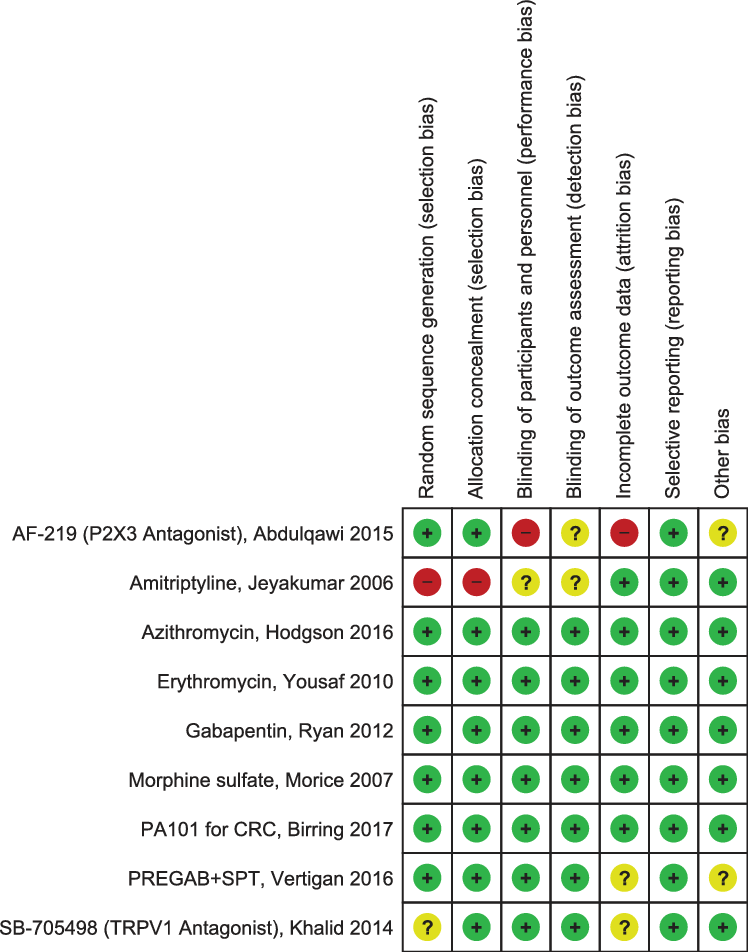 | 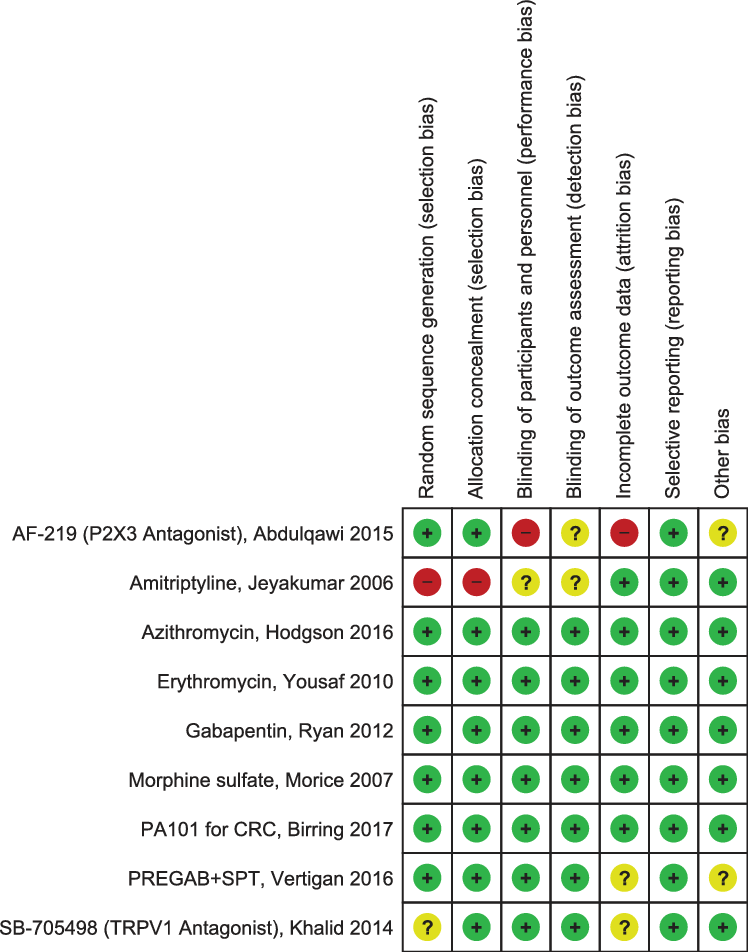 | 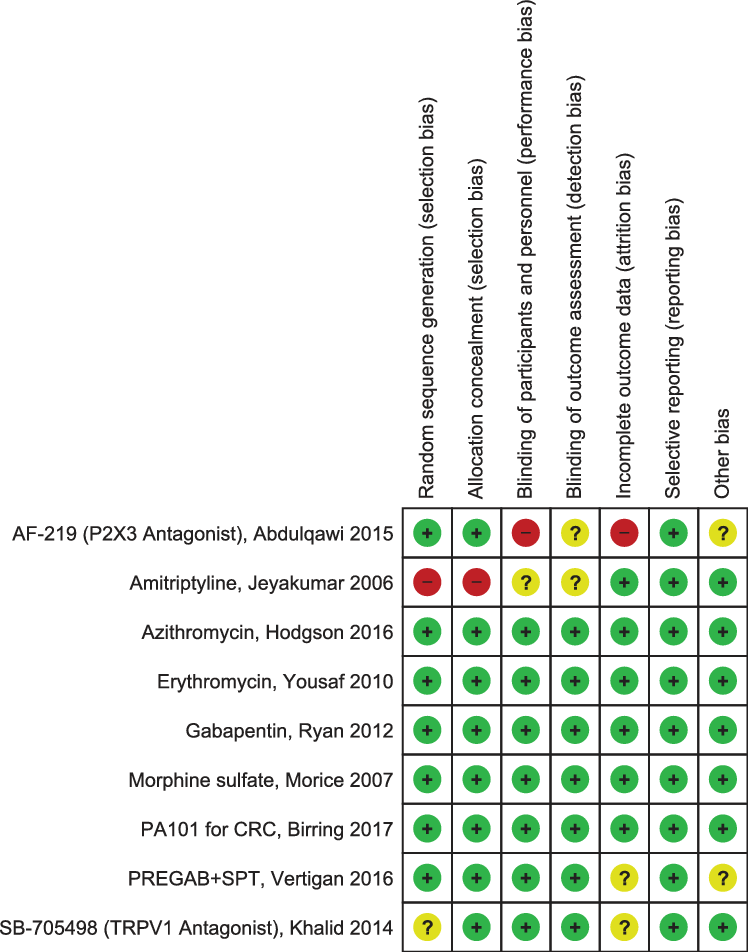 | 7 |
| Li et al., 2019 | RCT | 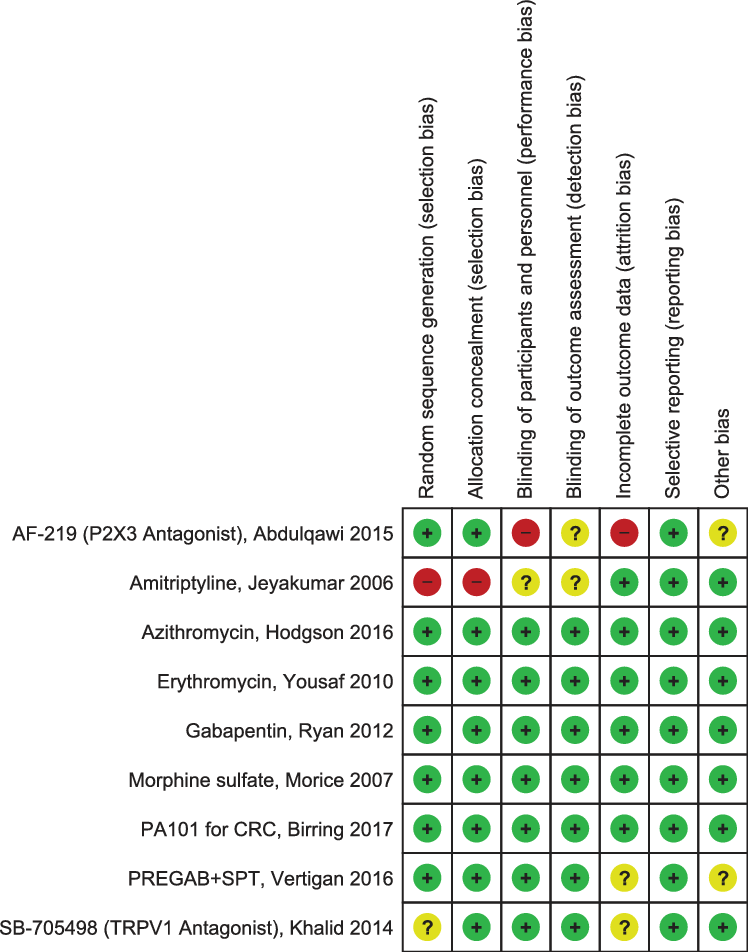 | 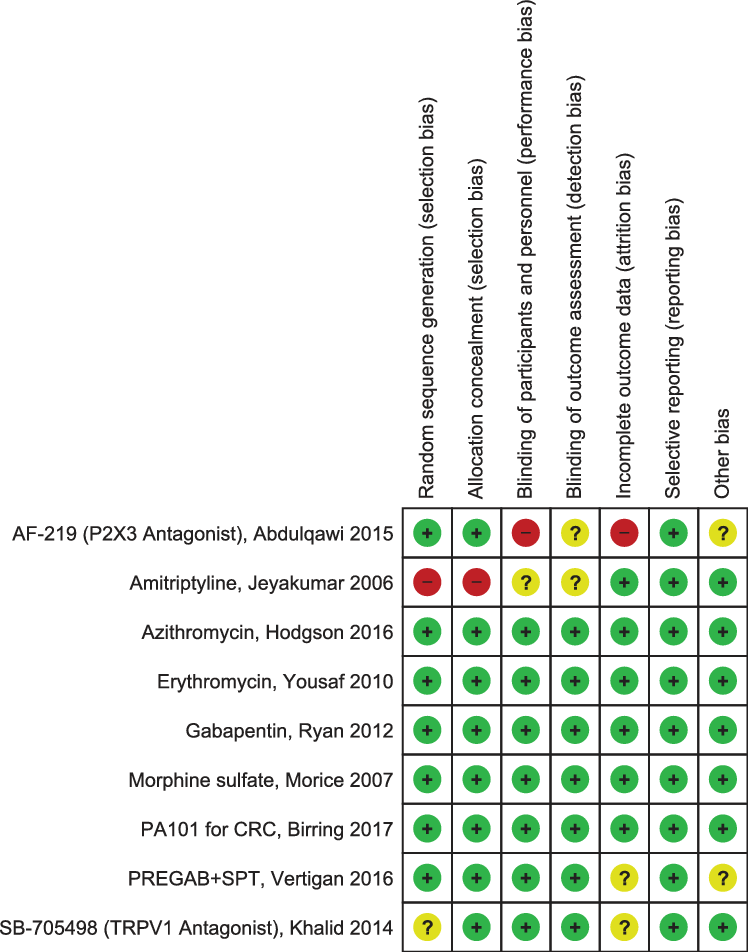 | 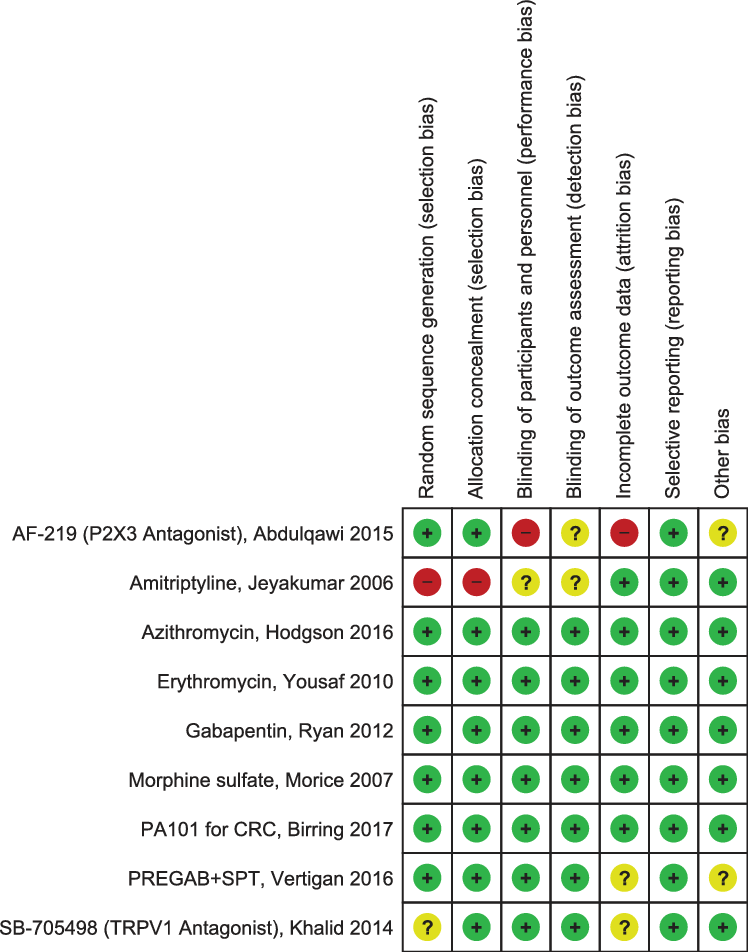 | 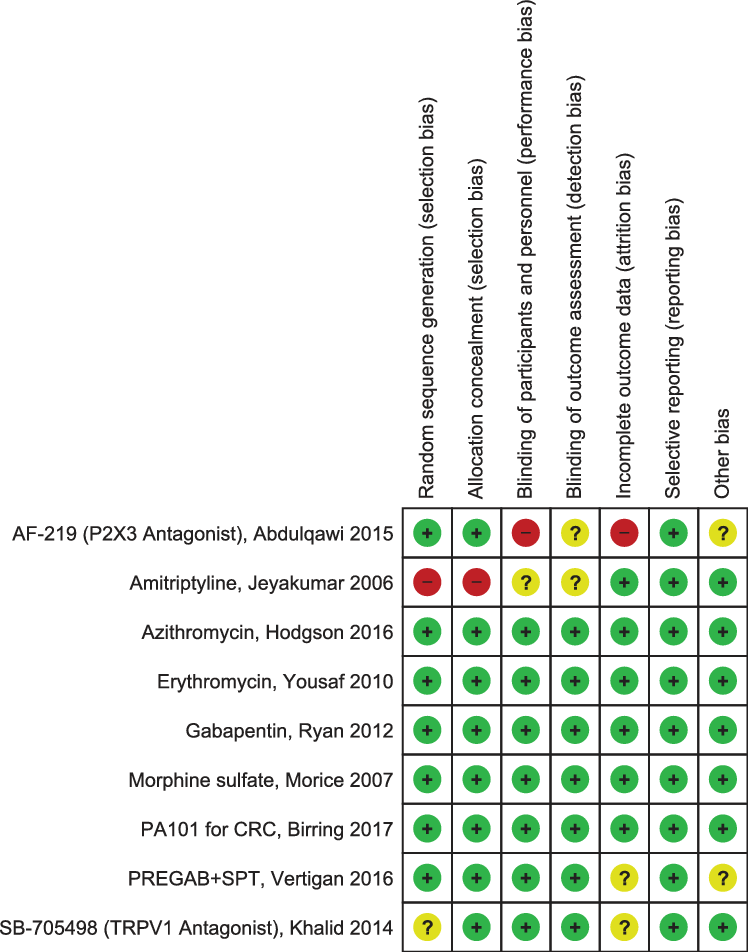 | 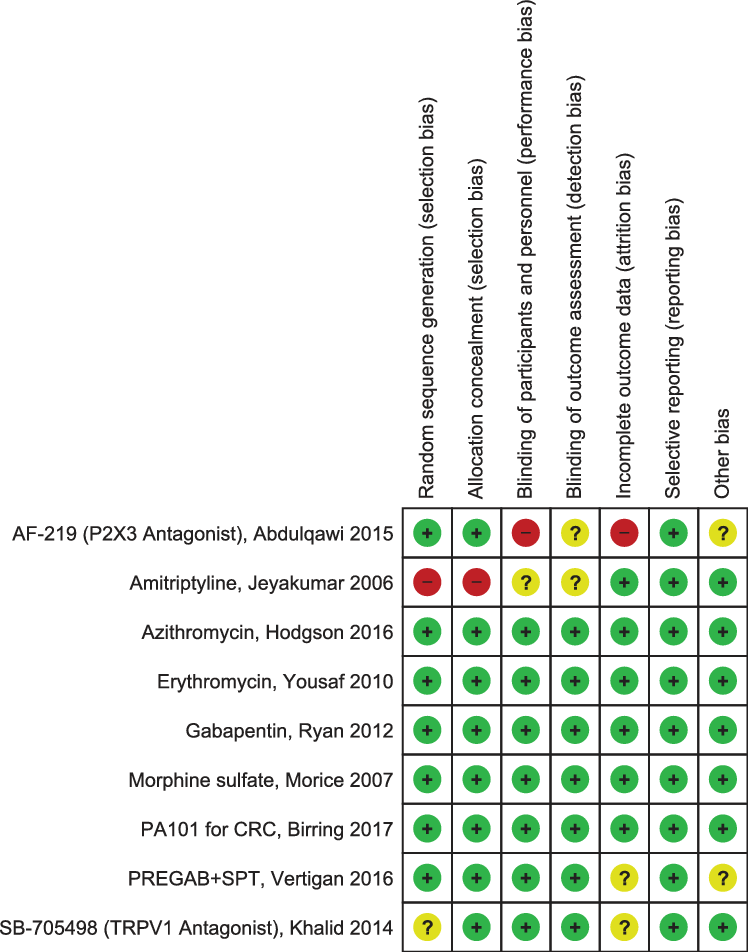 | 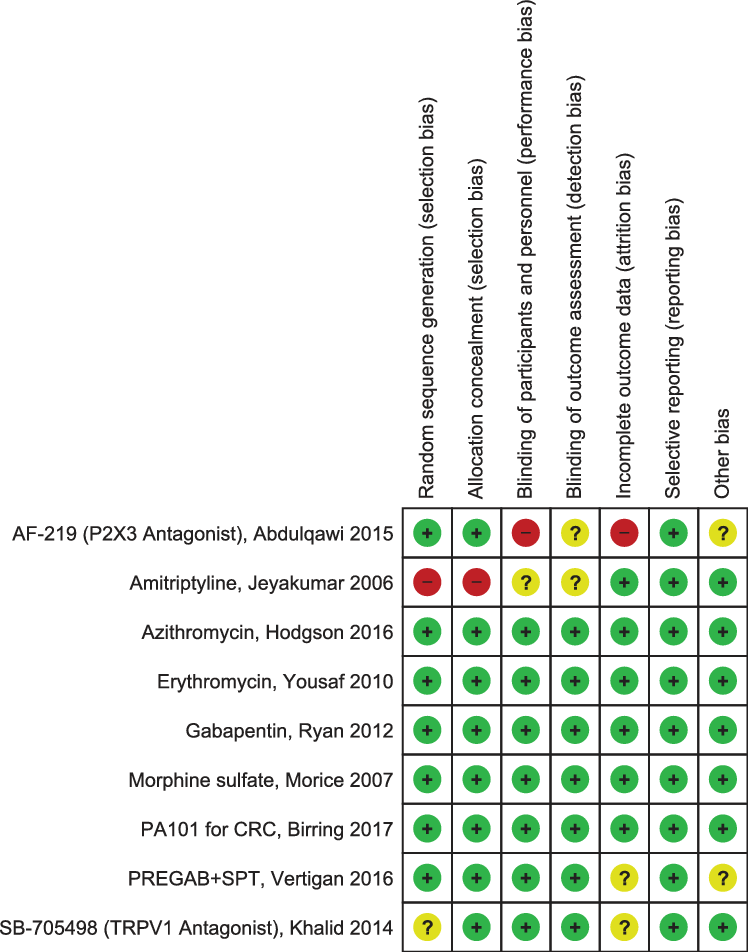 | 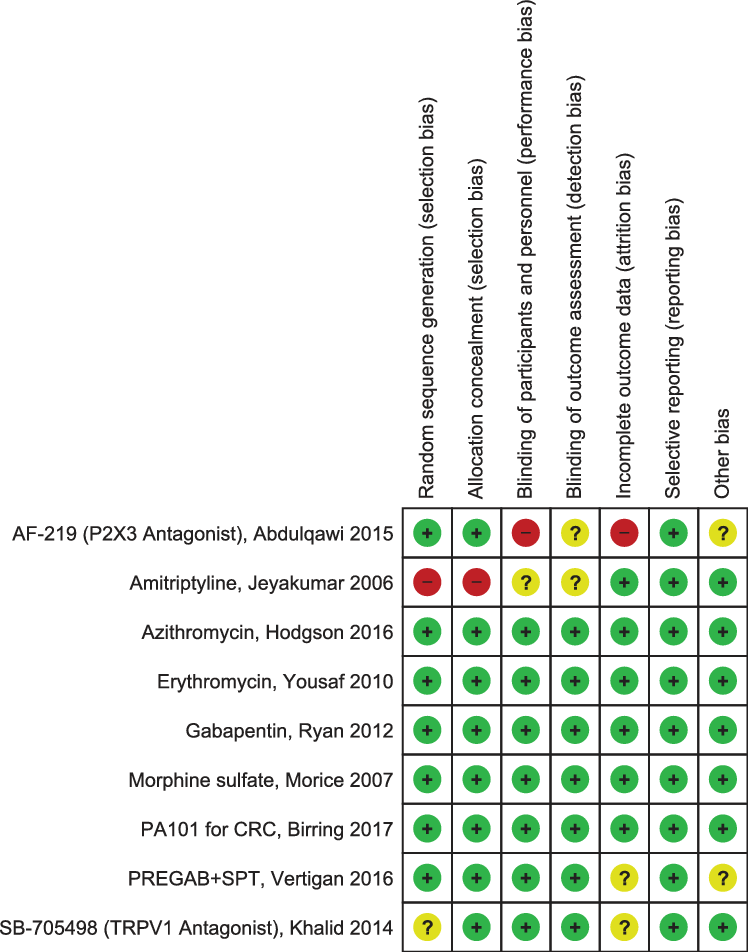 | 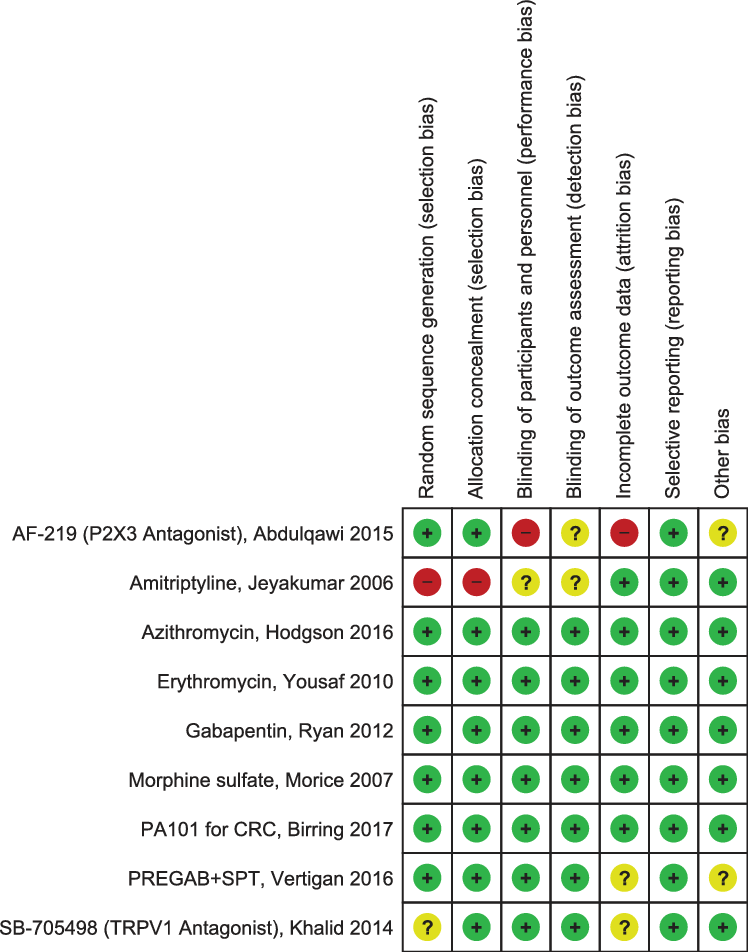 | 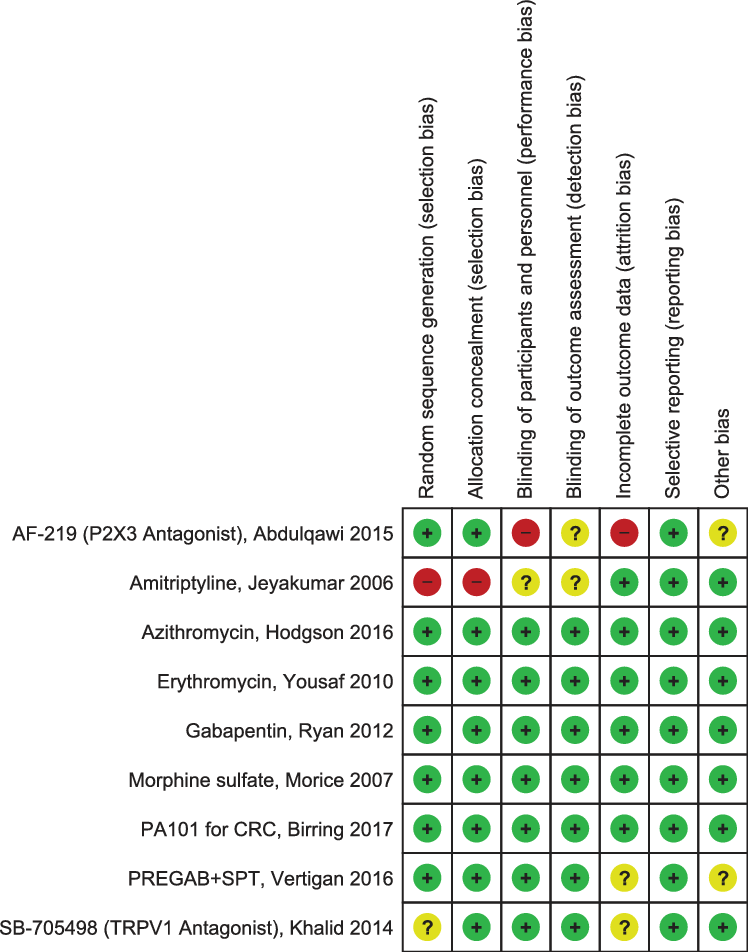 | 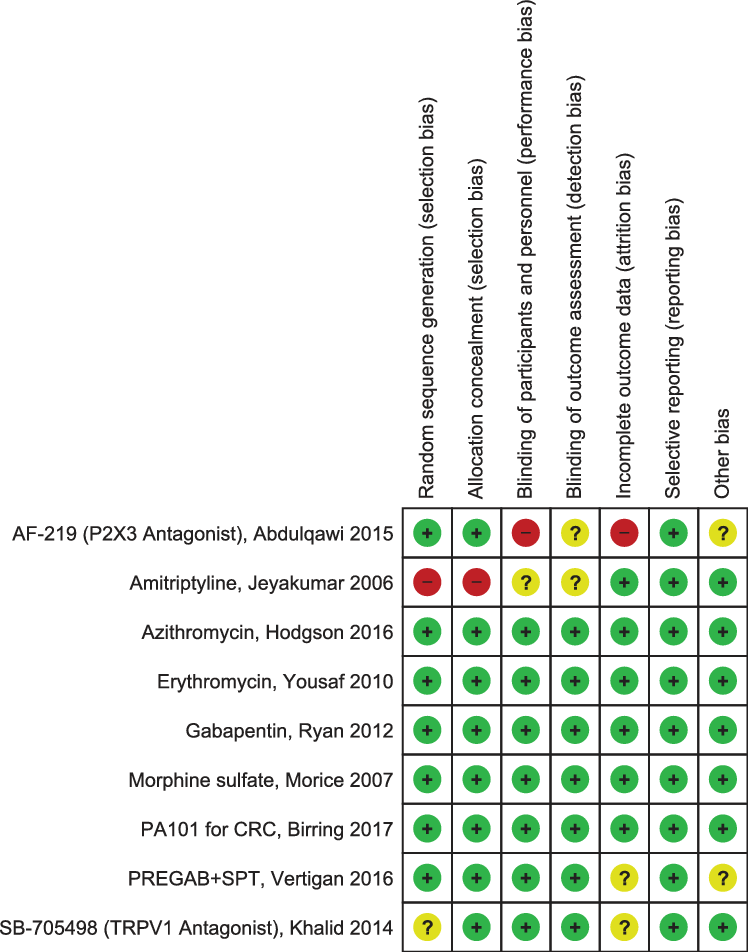 | 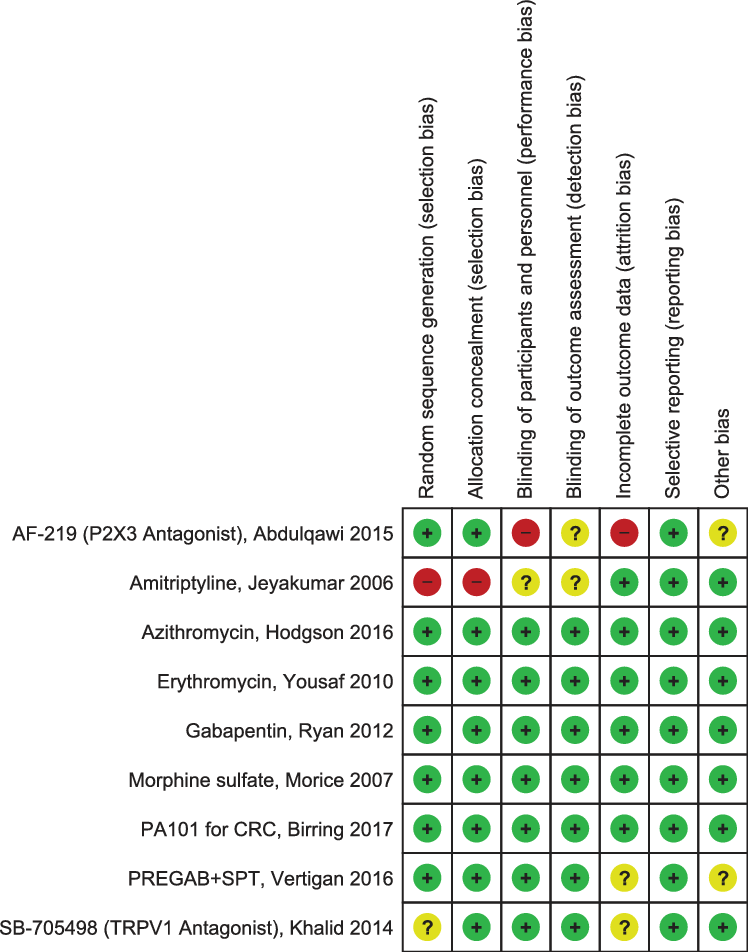 | 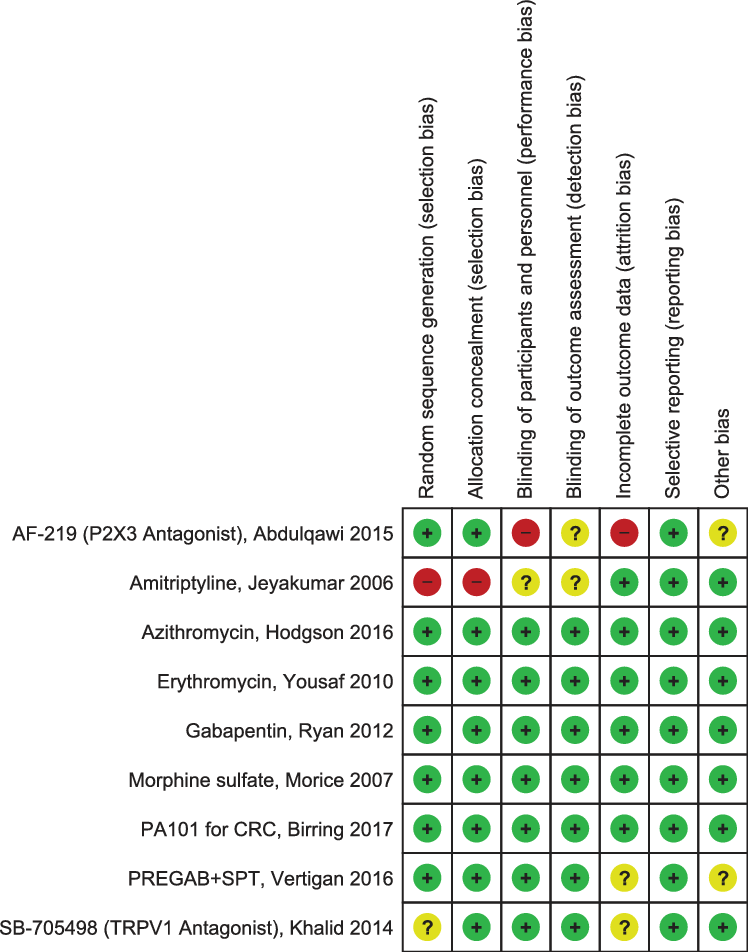 | 6 |
| Li et al., 2016 | RCT | 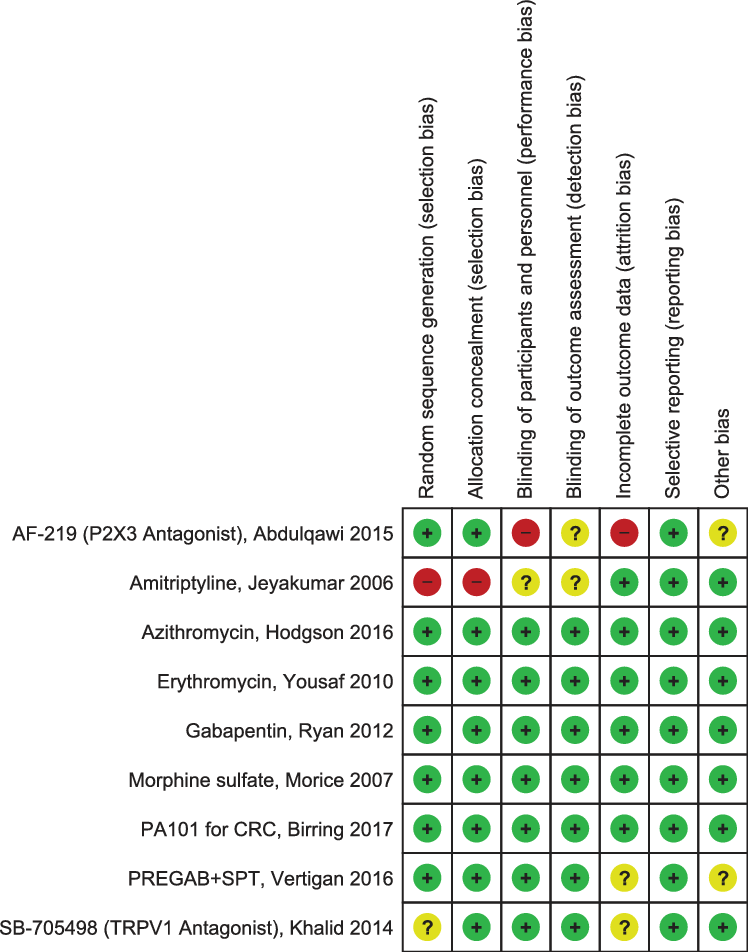 | 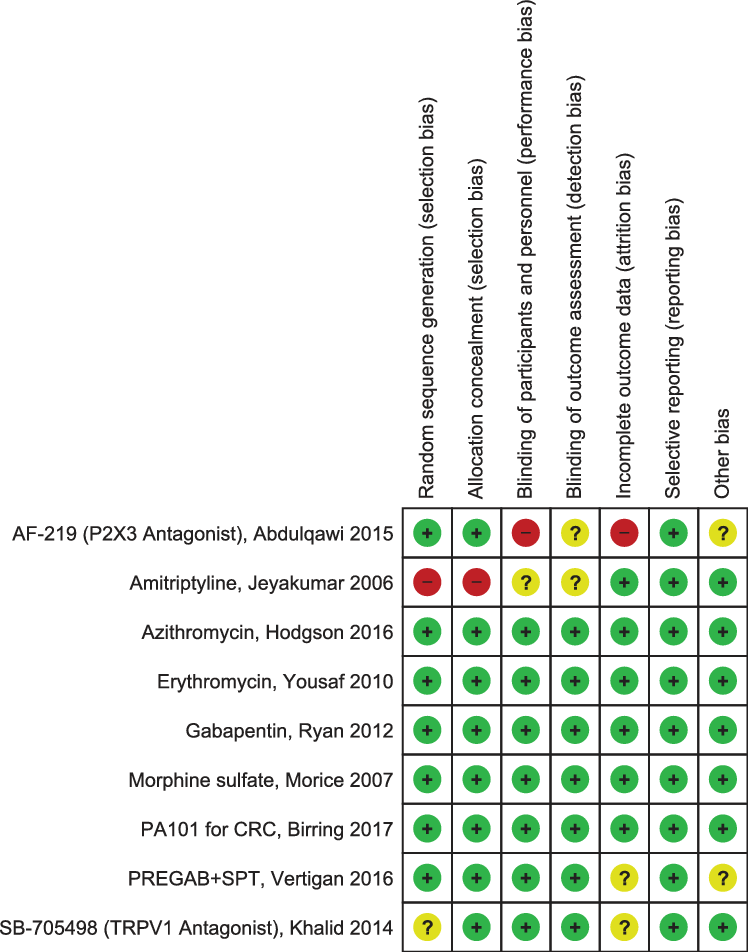 | 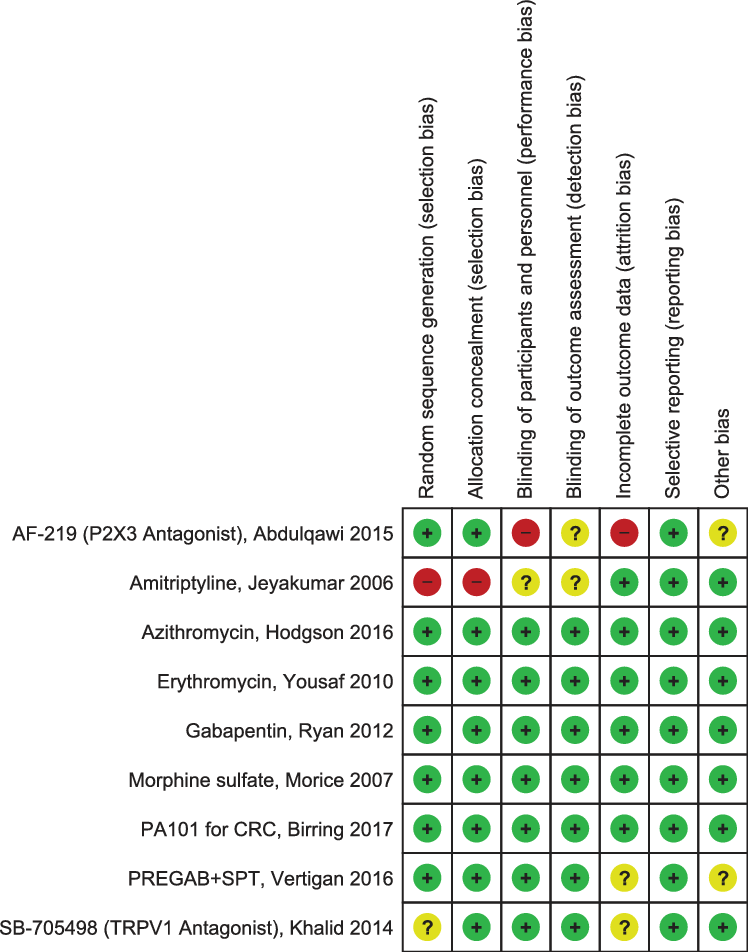 | 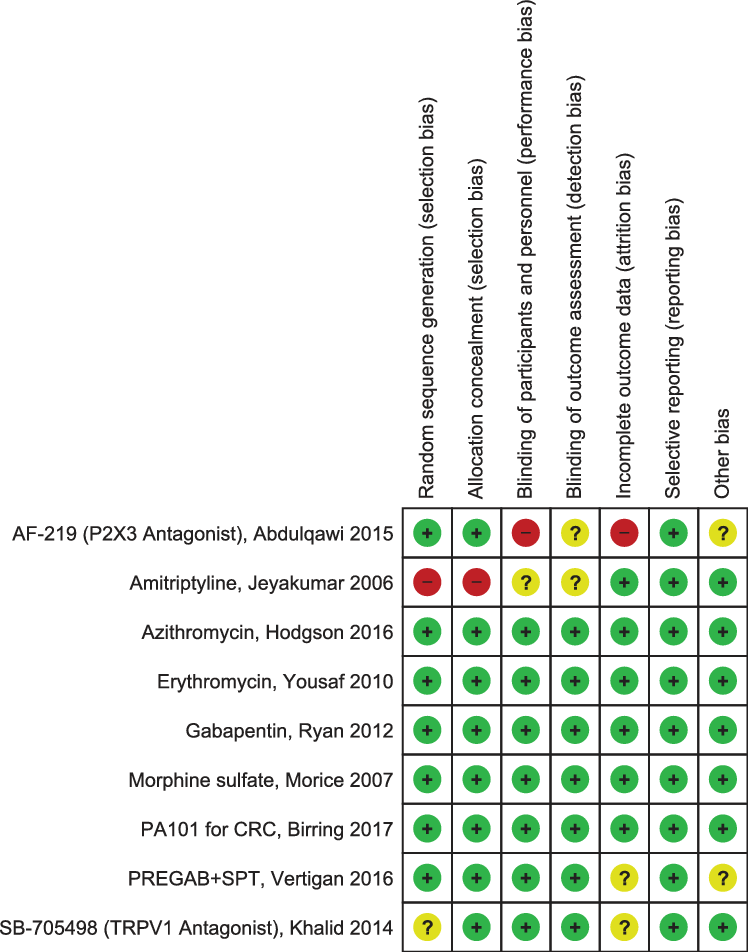 | 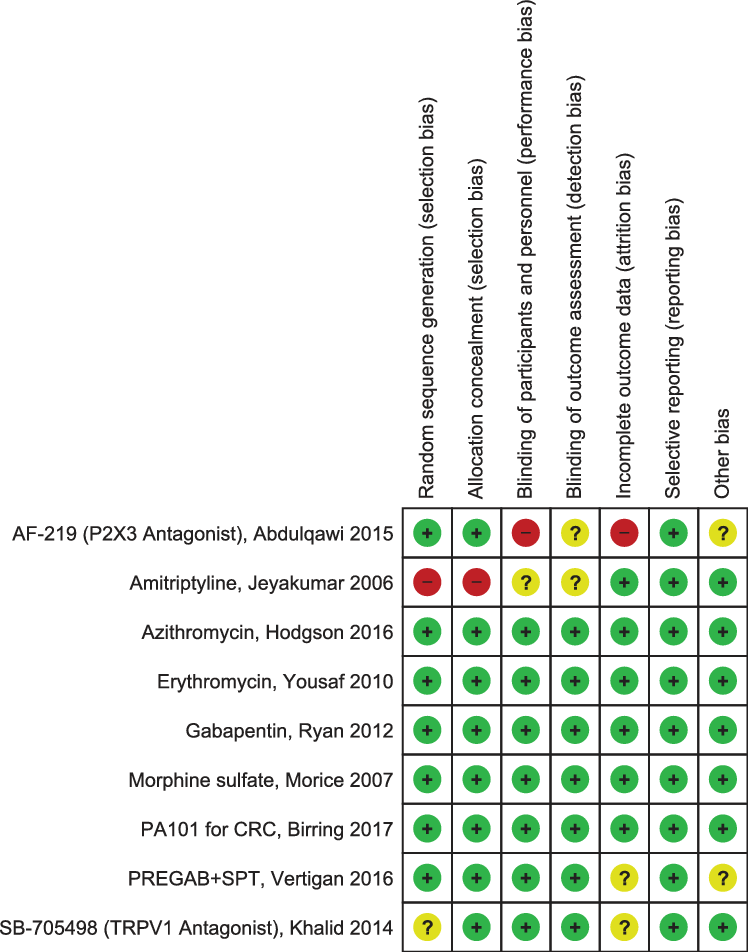 | 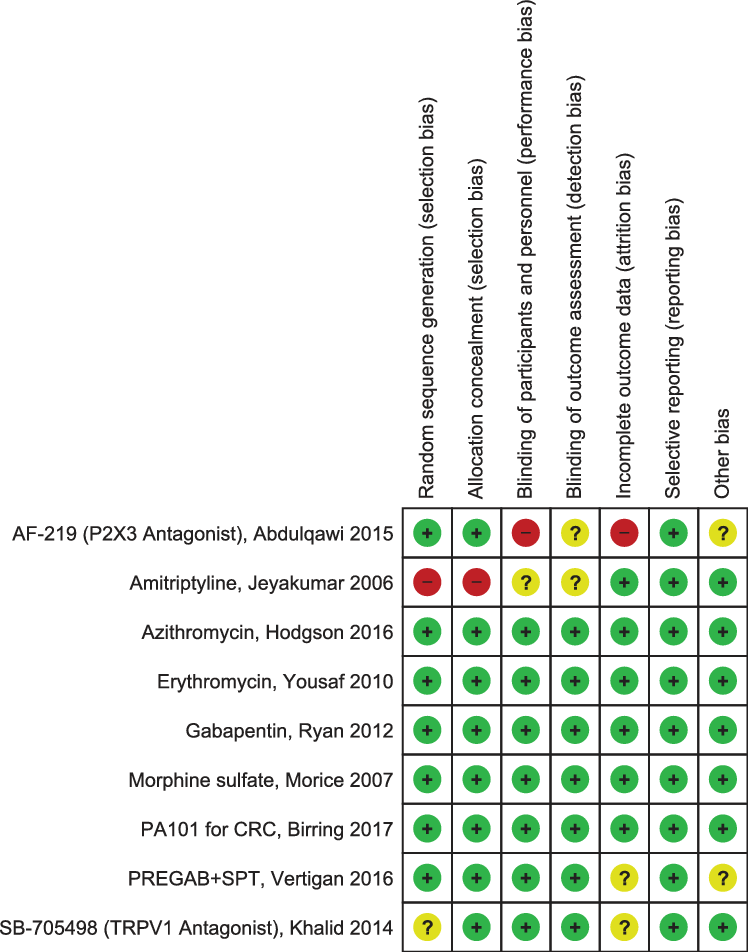 | 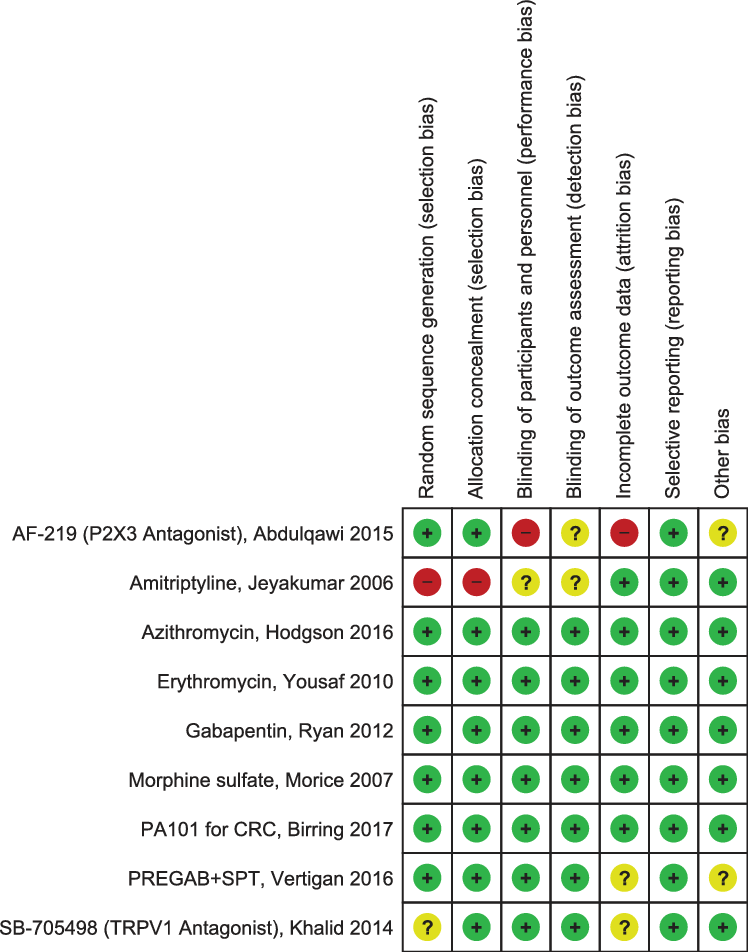 | 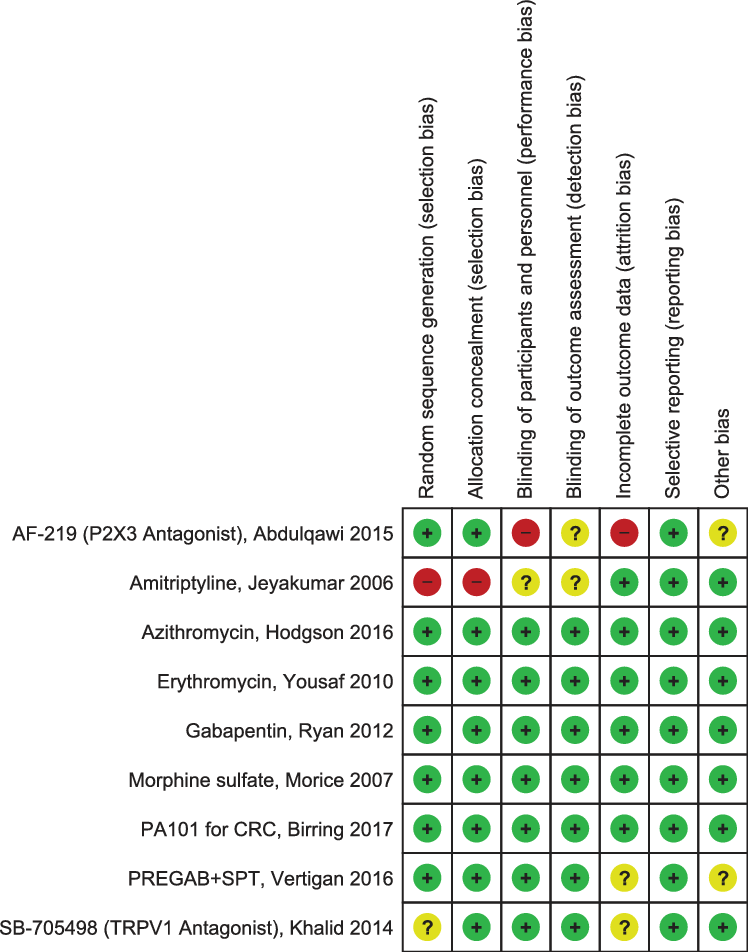 | 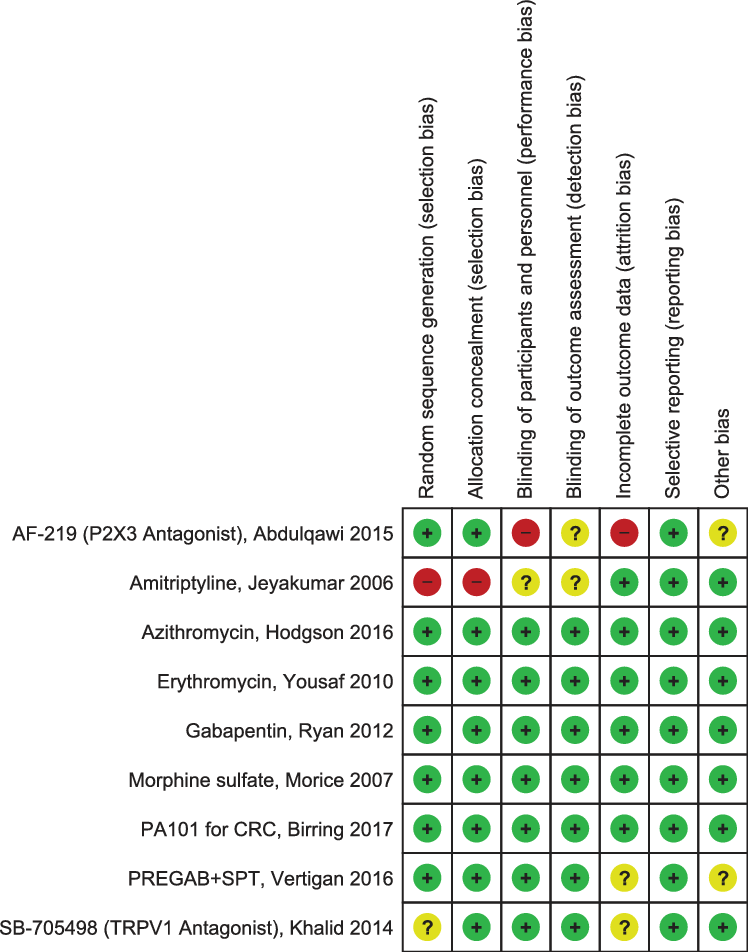 | 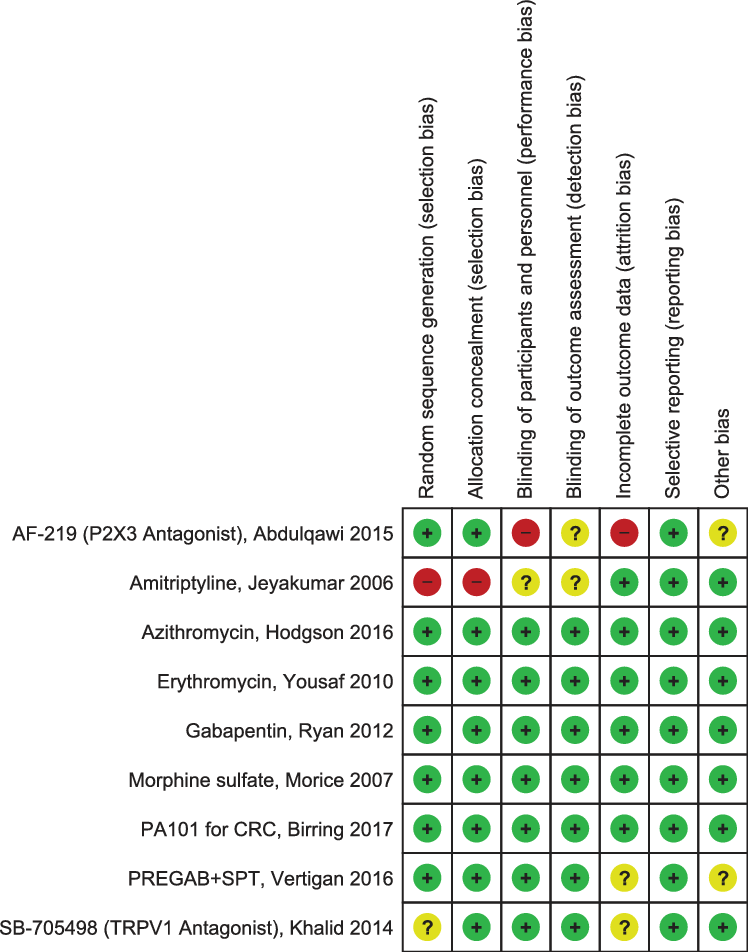 | 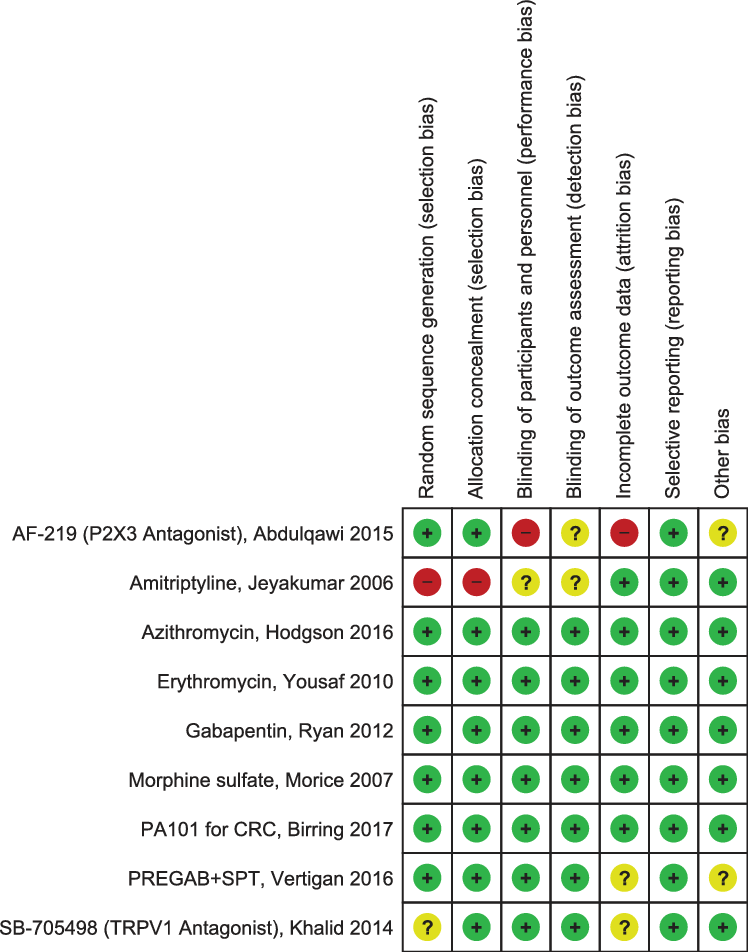 | 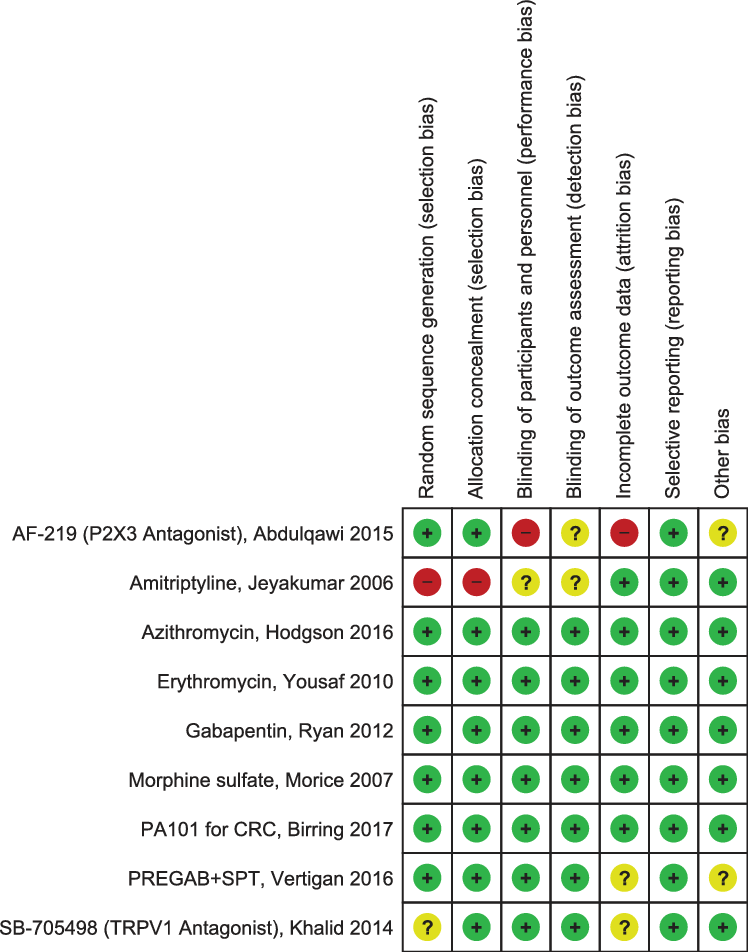 | 9 |
| Rutledge et al., 2014 | RCT | 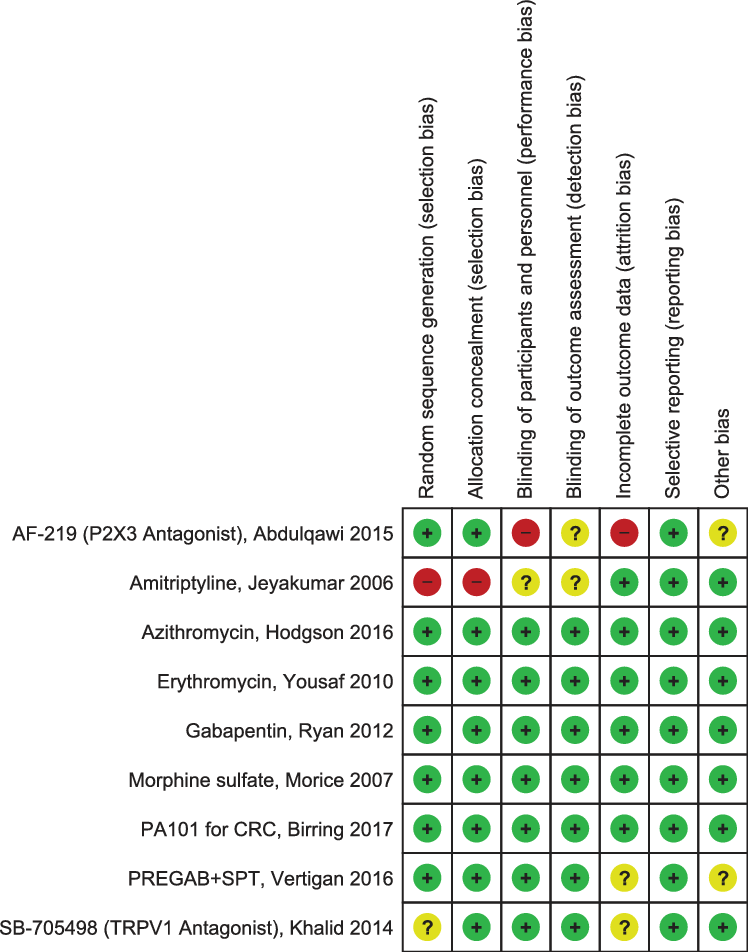 | 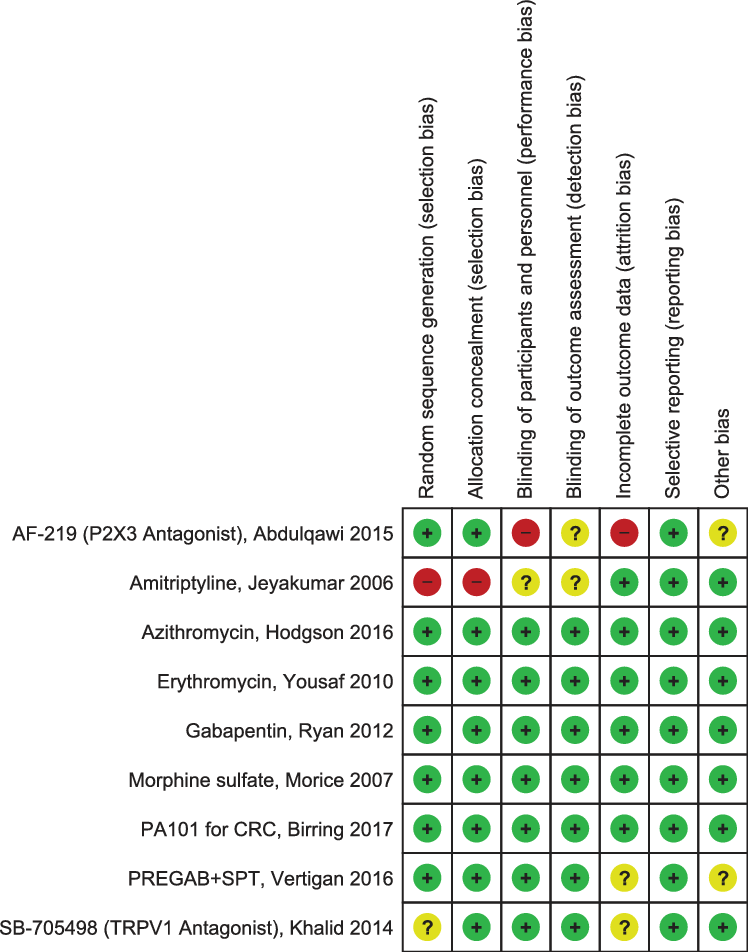 | 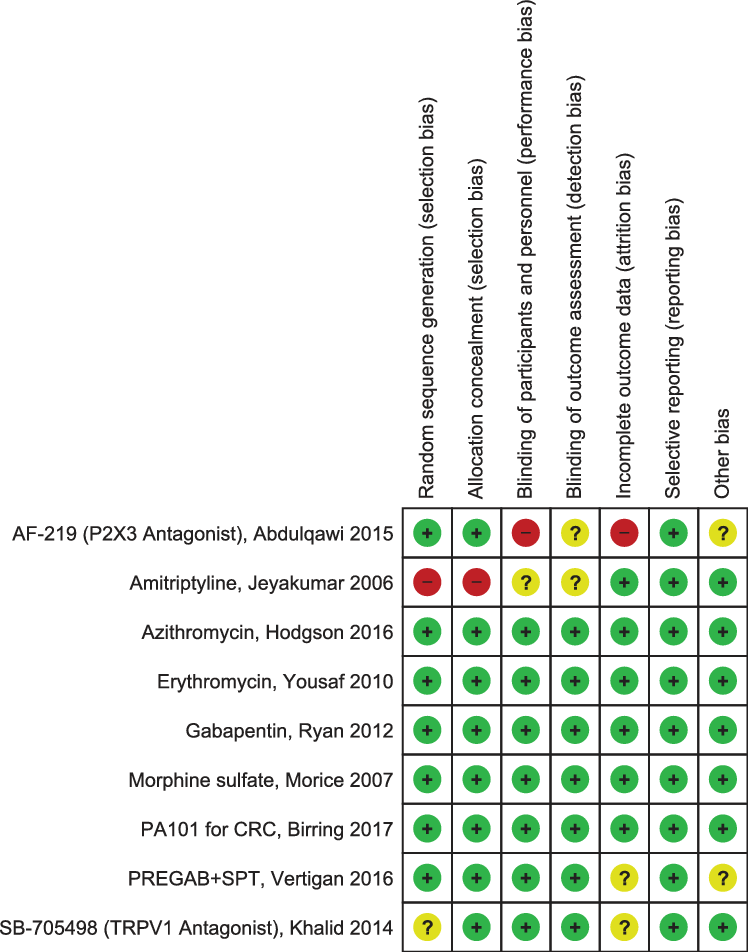 | 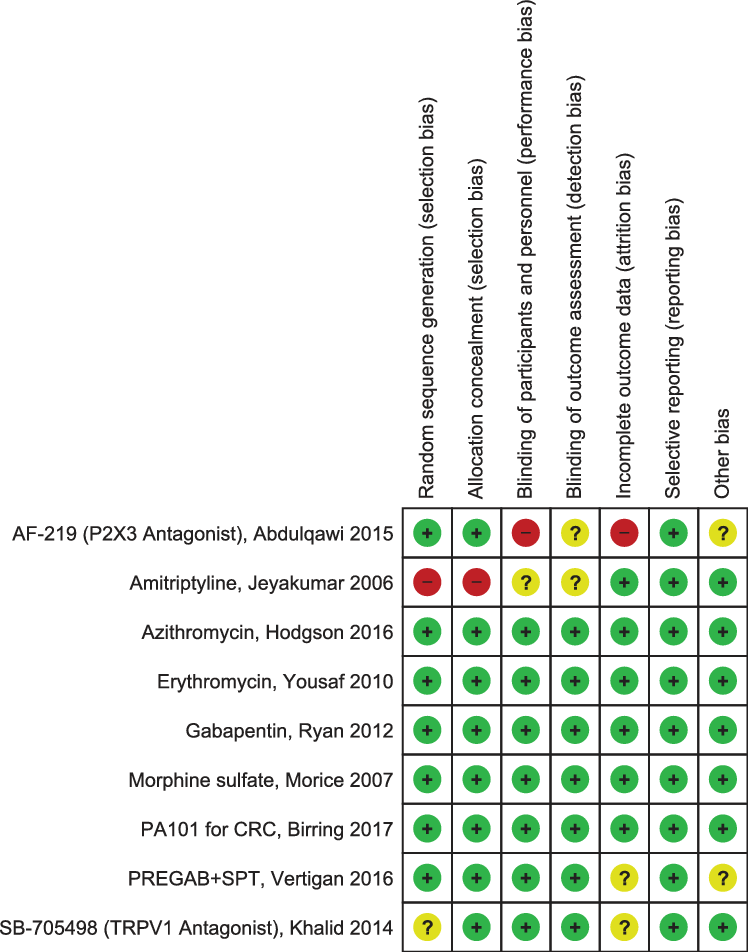 | 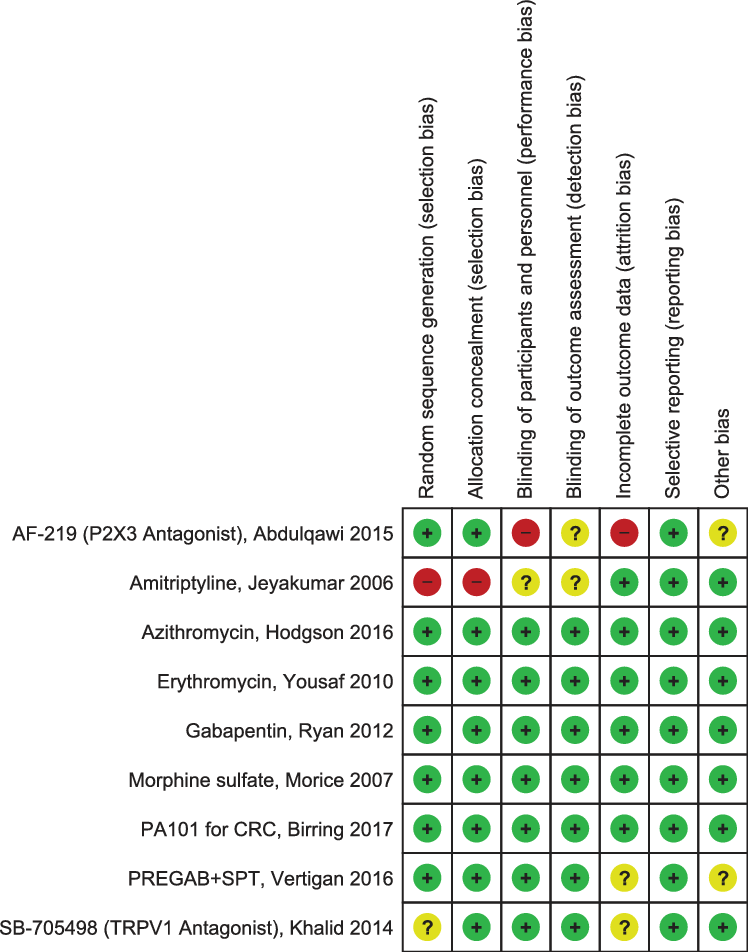 | 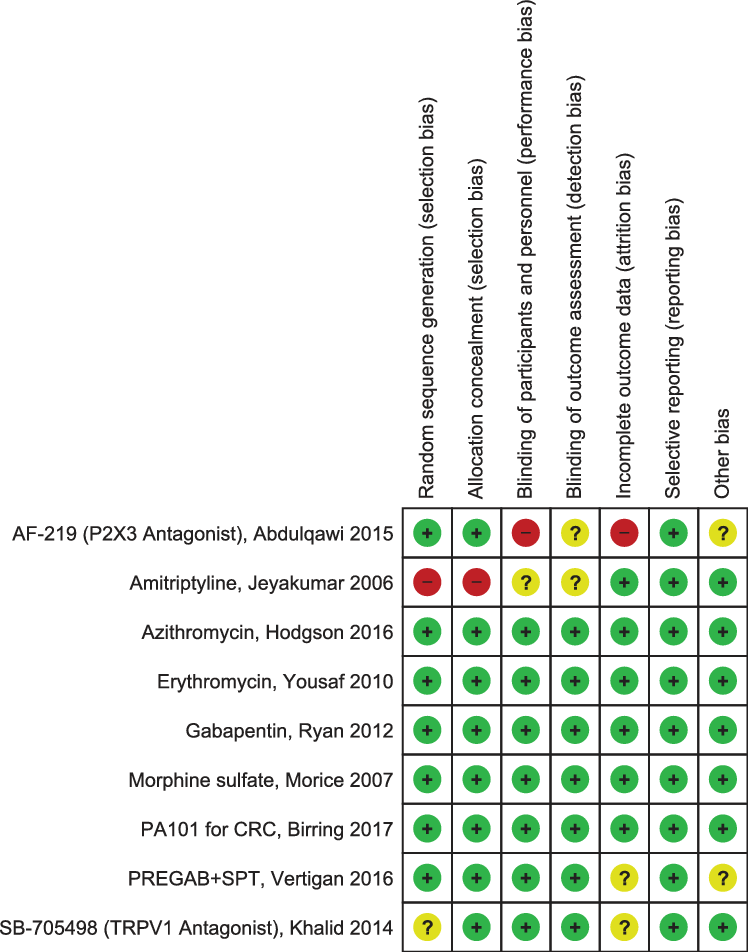 | 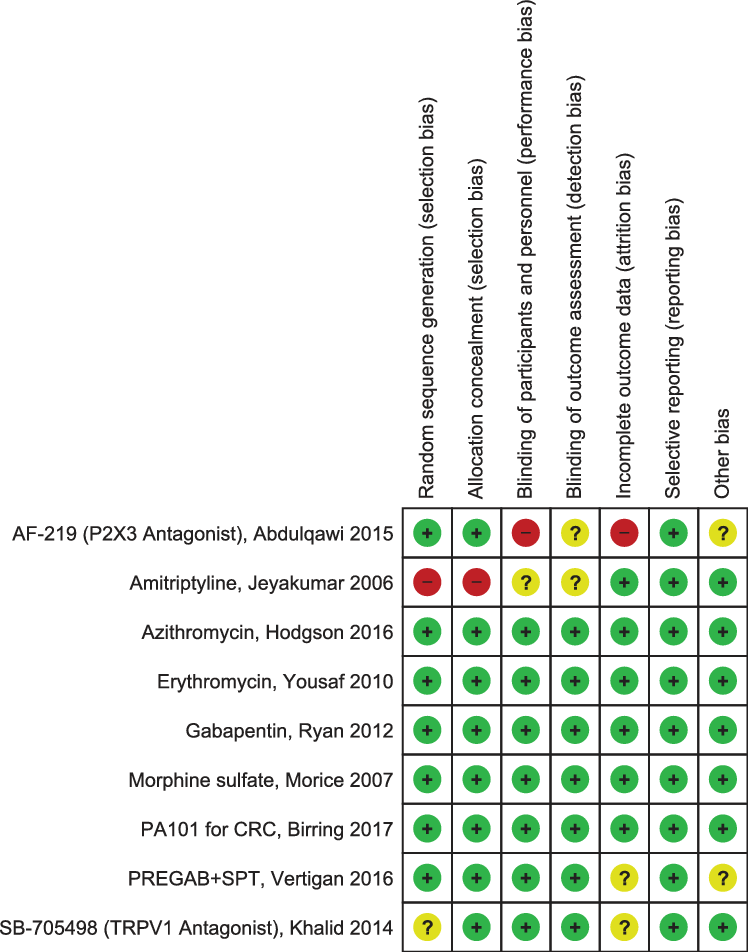 | 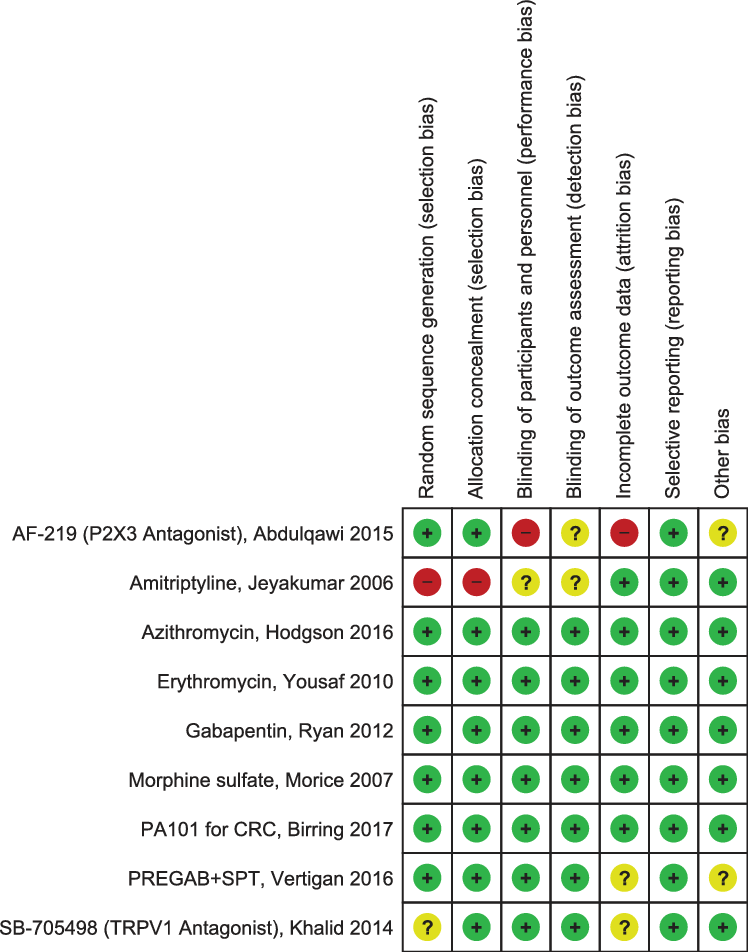 | 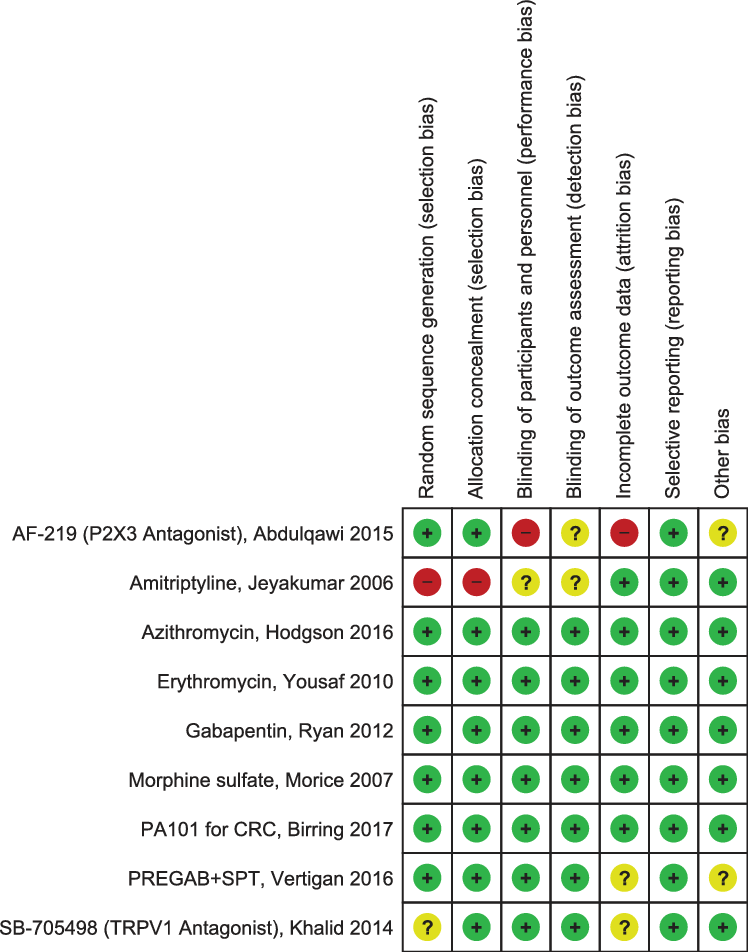 | 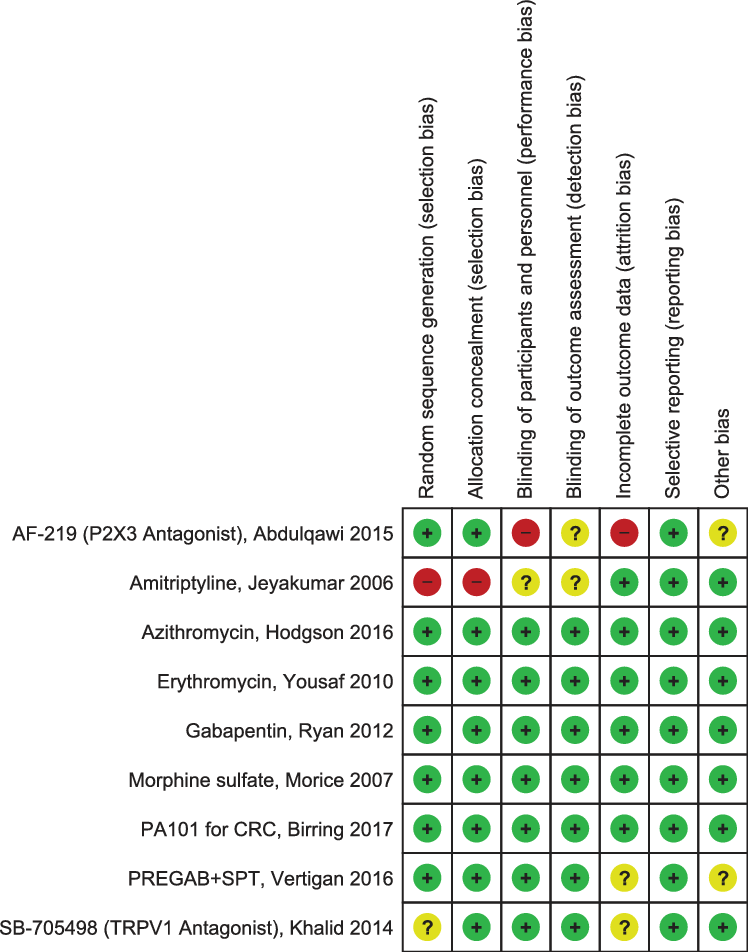 | 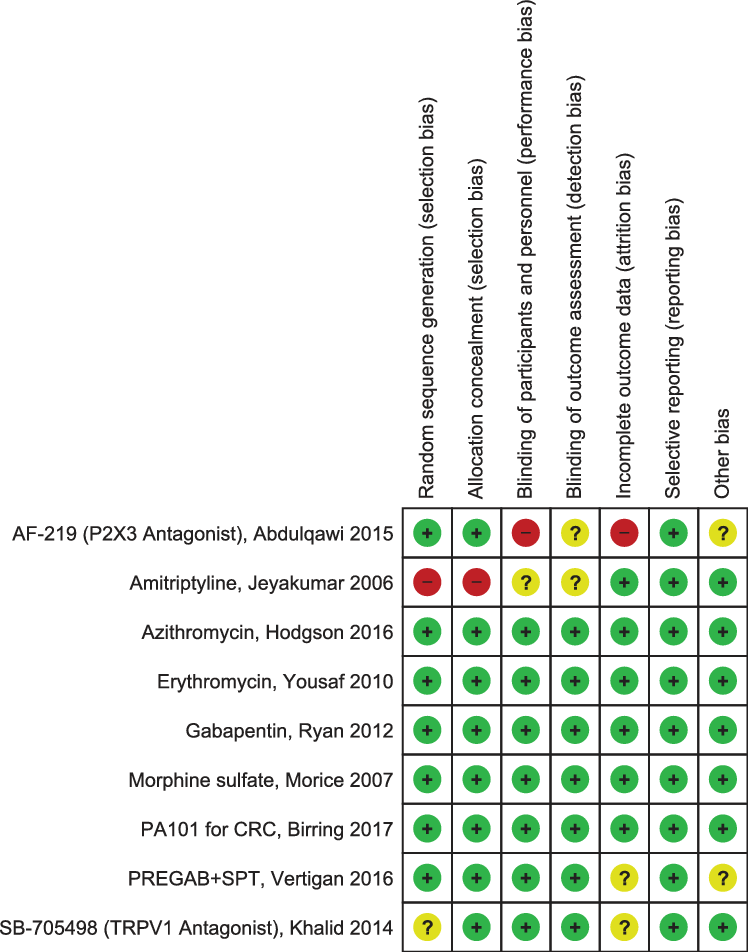 | 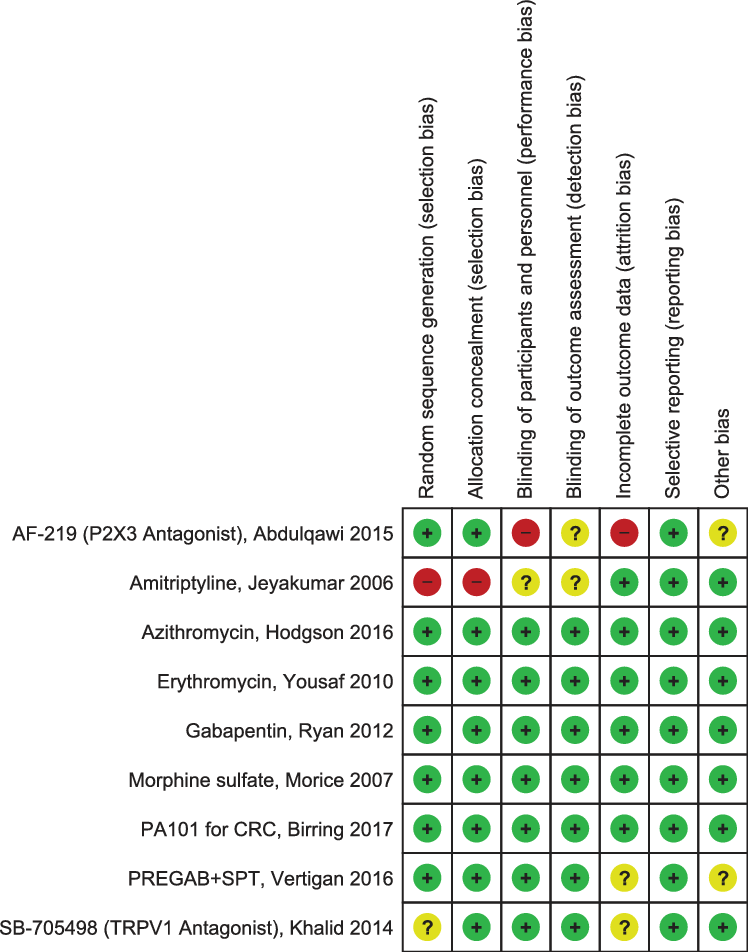 | 11 |
| Yang et al., 2012 | RCT | 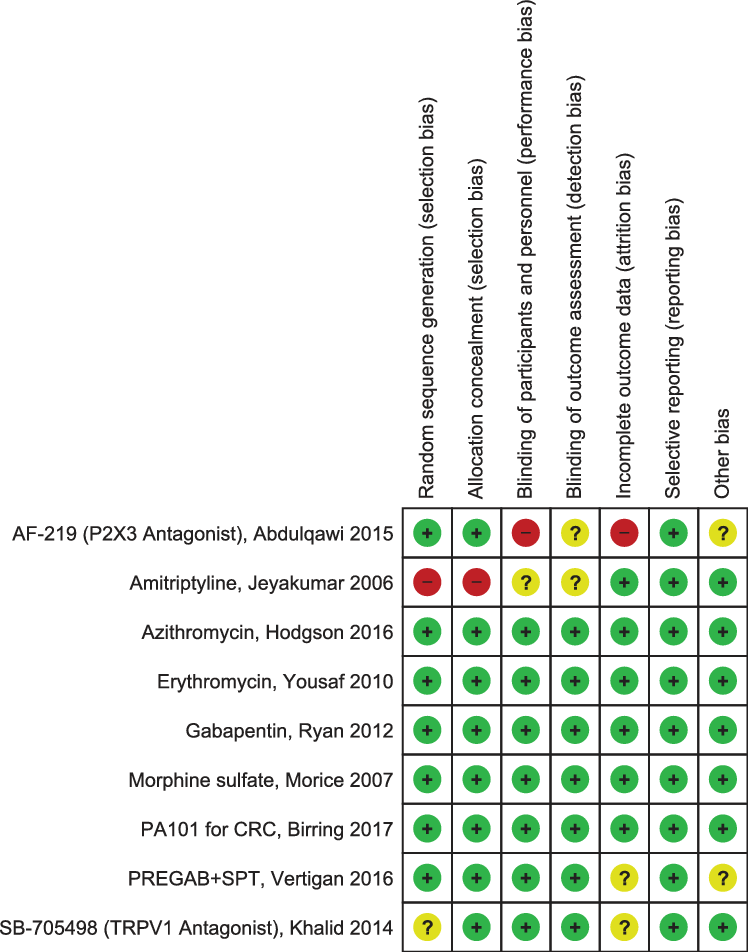 | 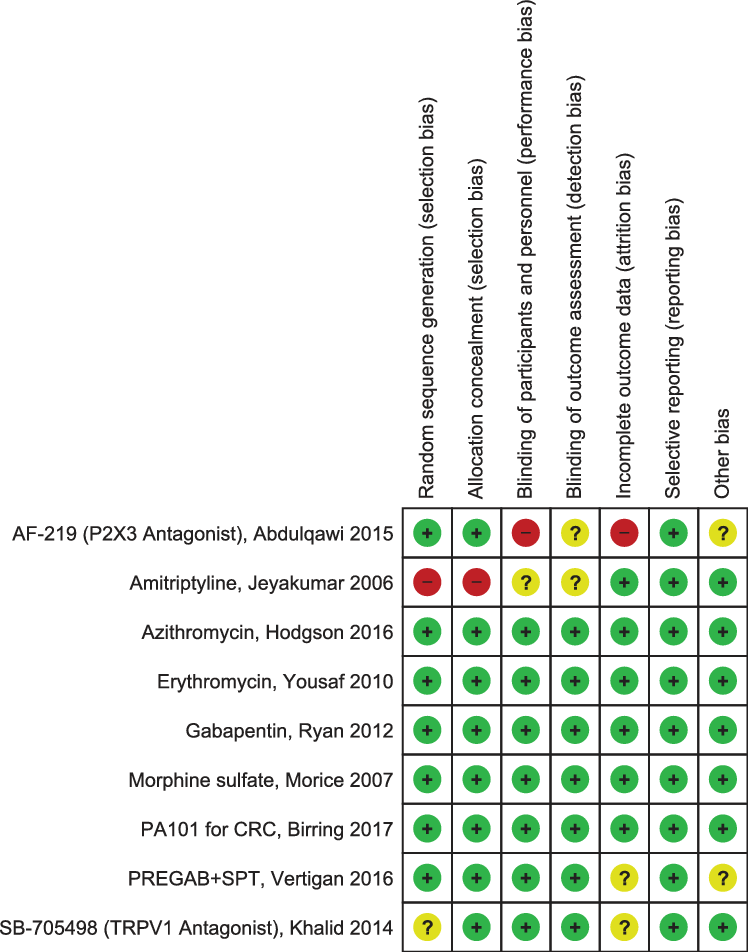 | 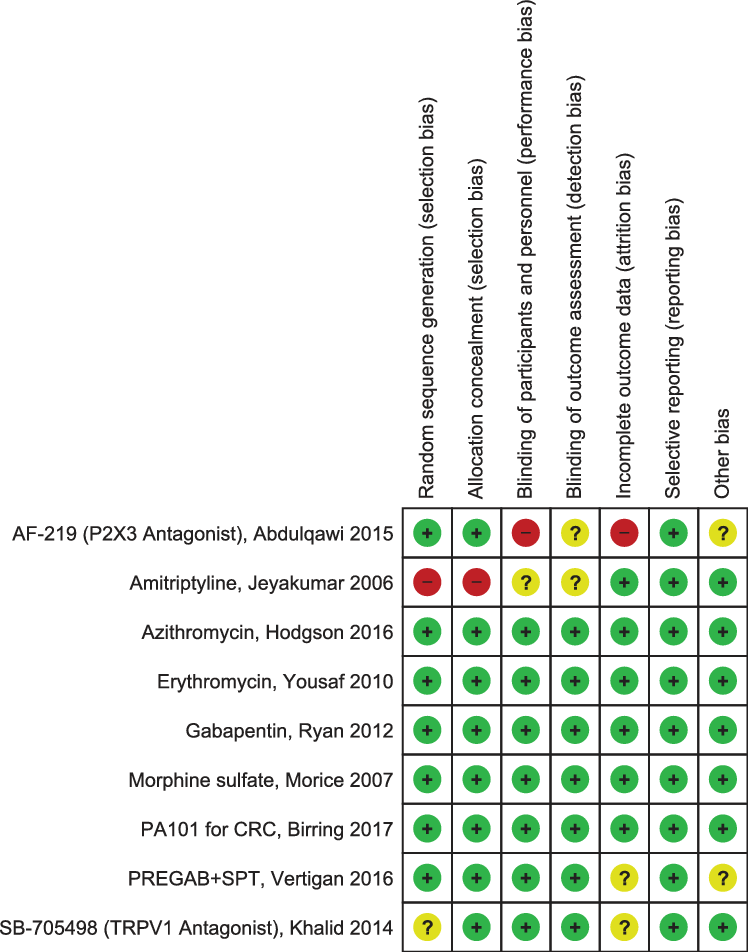 | 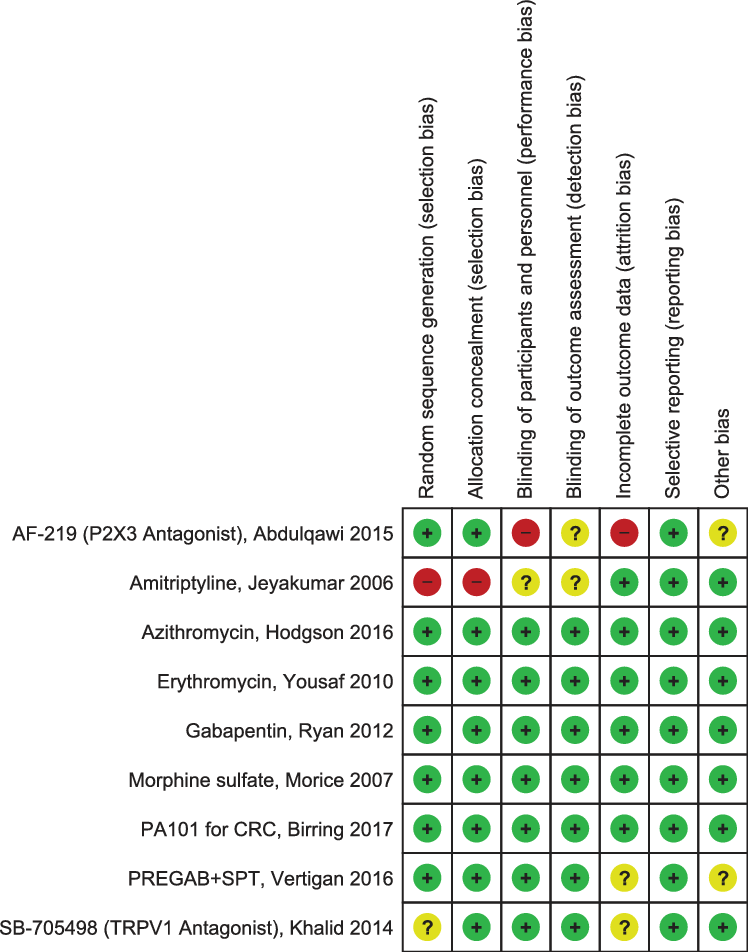 | 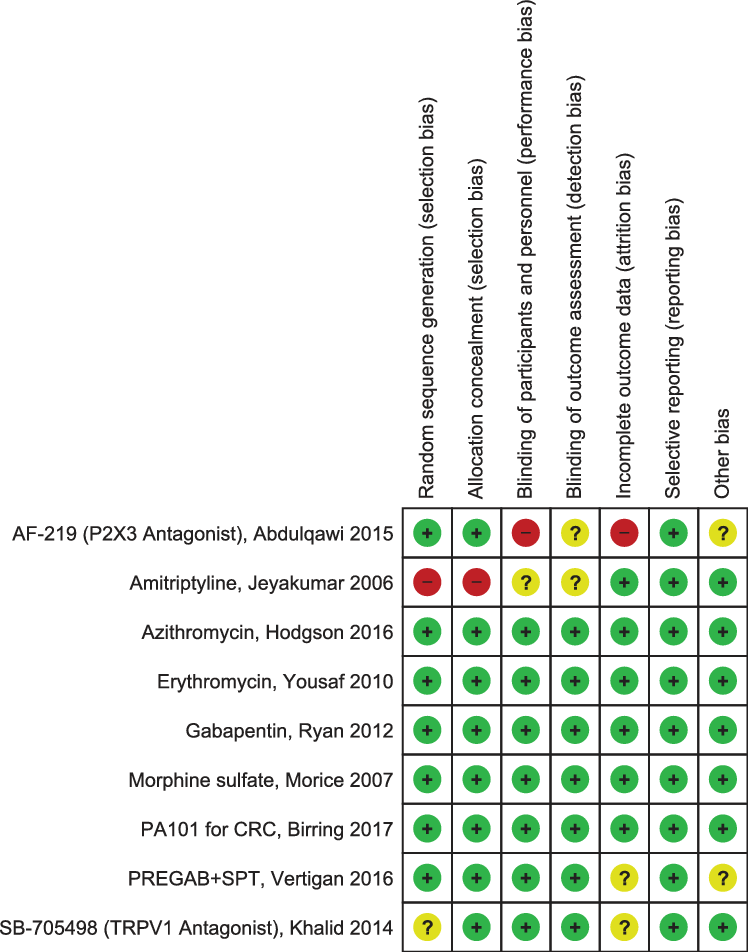 | 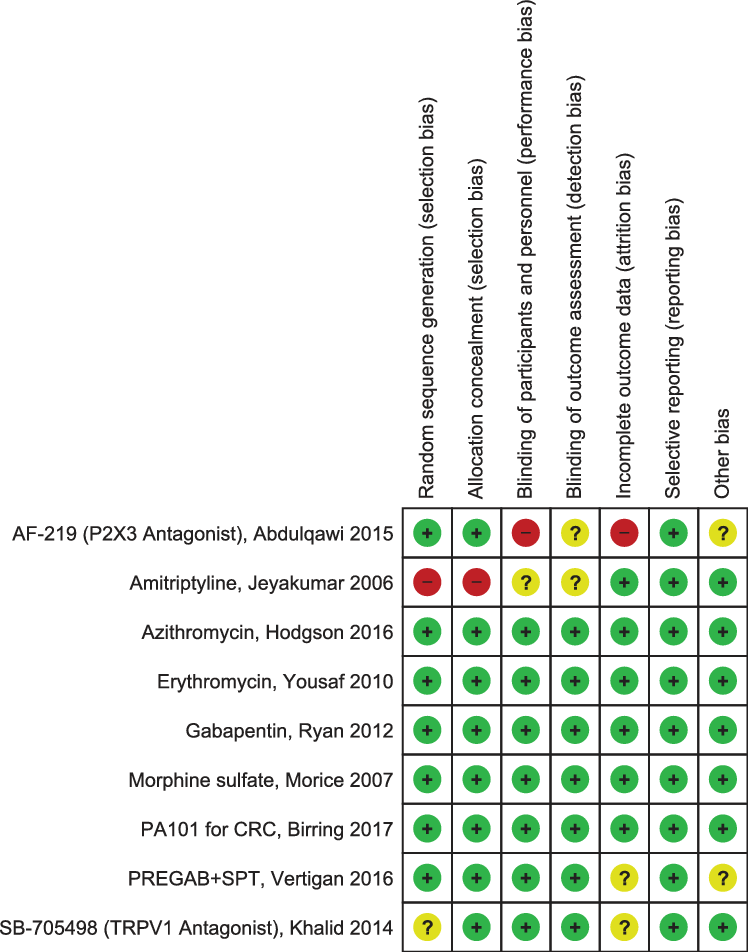 | 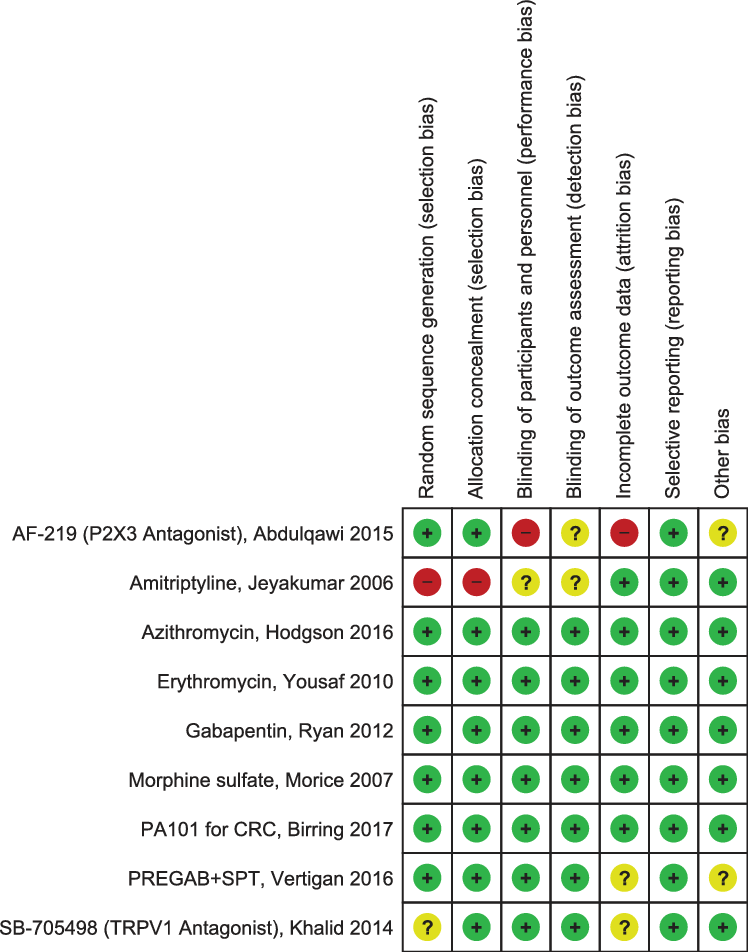 | 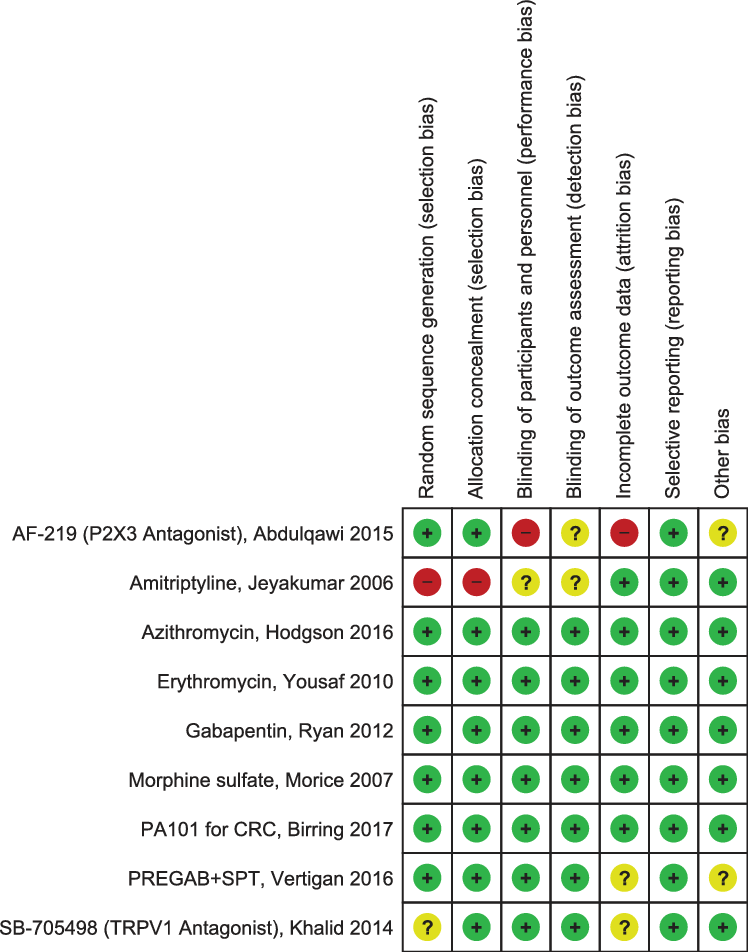 | 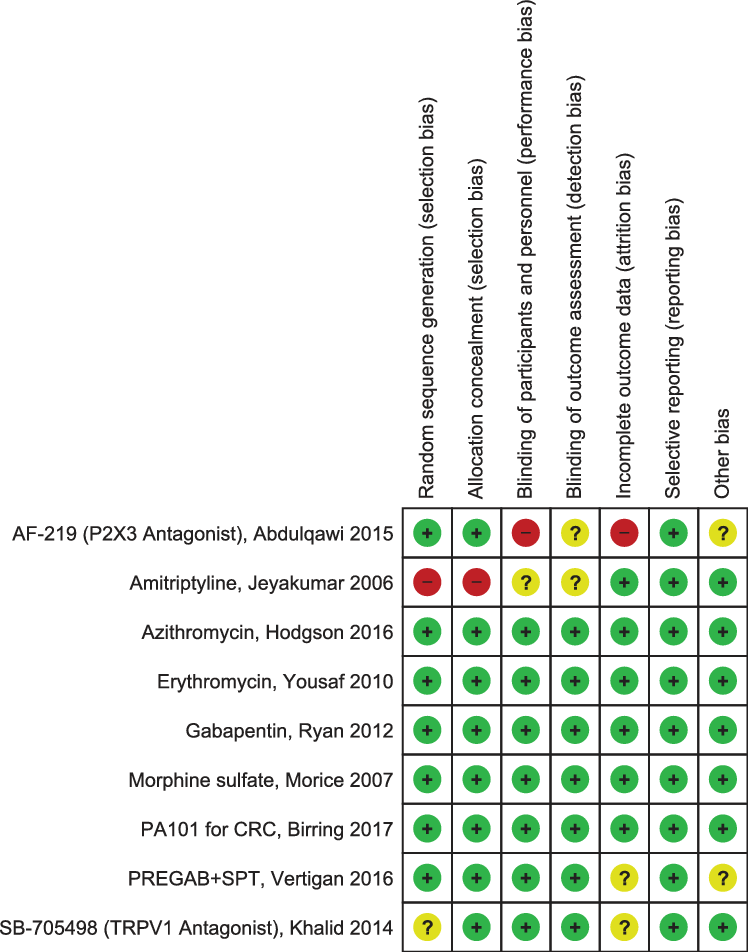 | 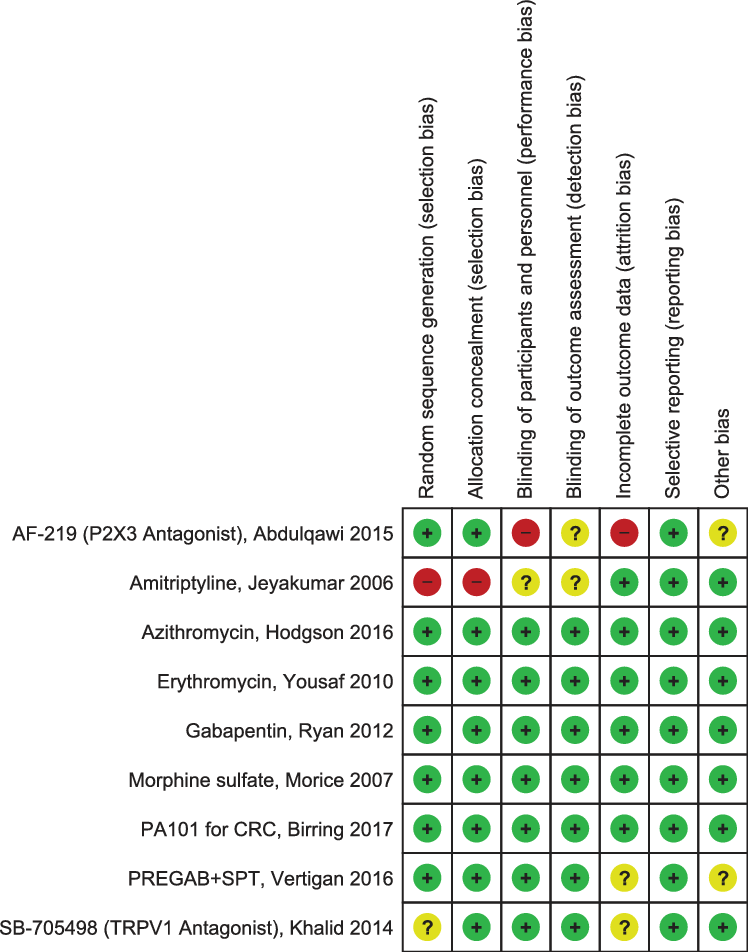 | 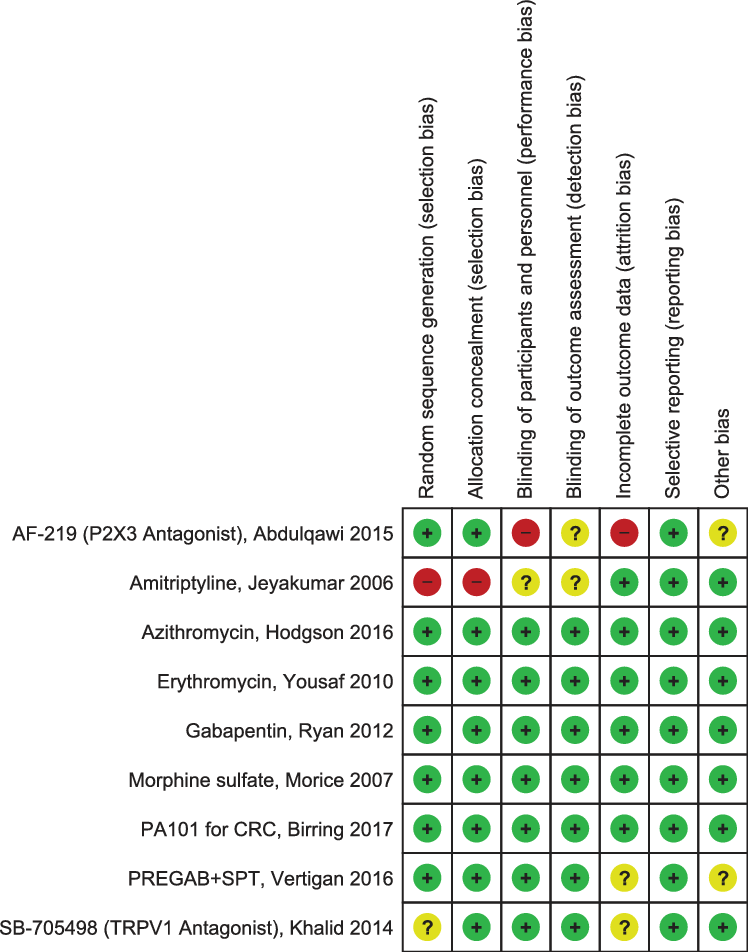 | 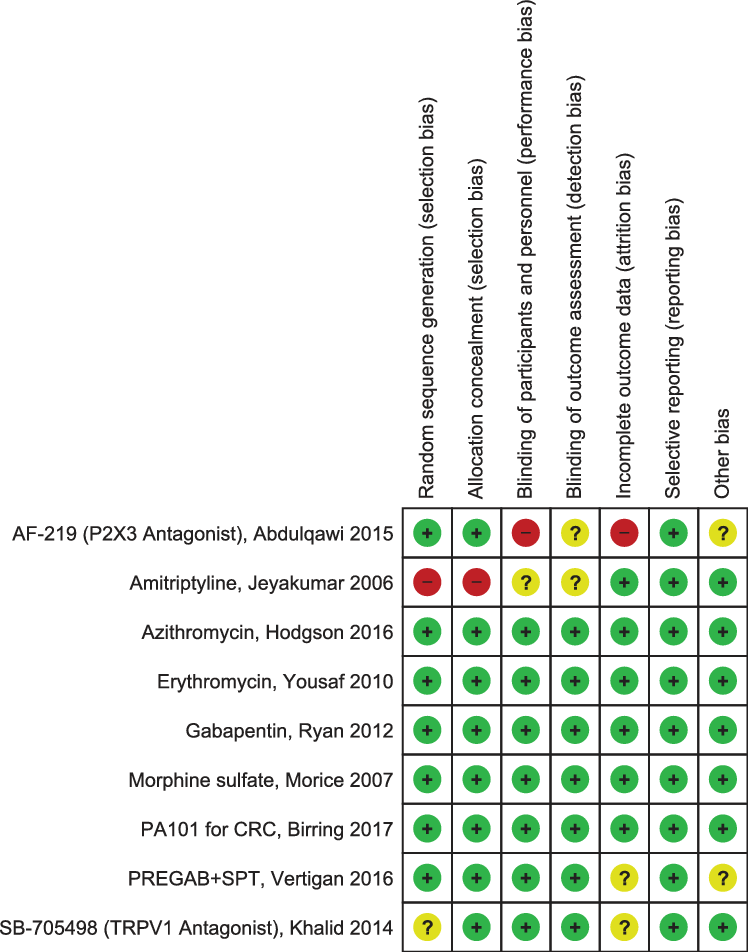 | 9 |
| Cerentini et al., 2019 | RCT | 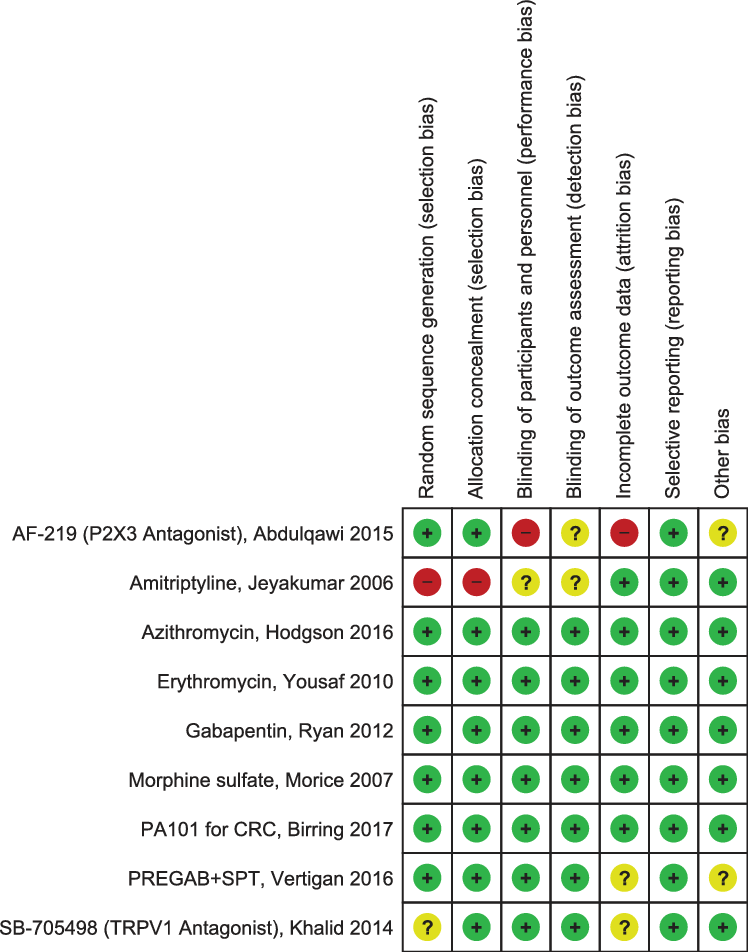 | 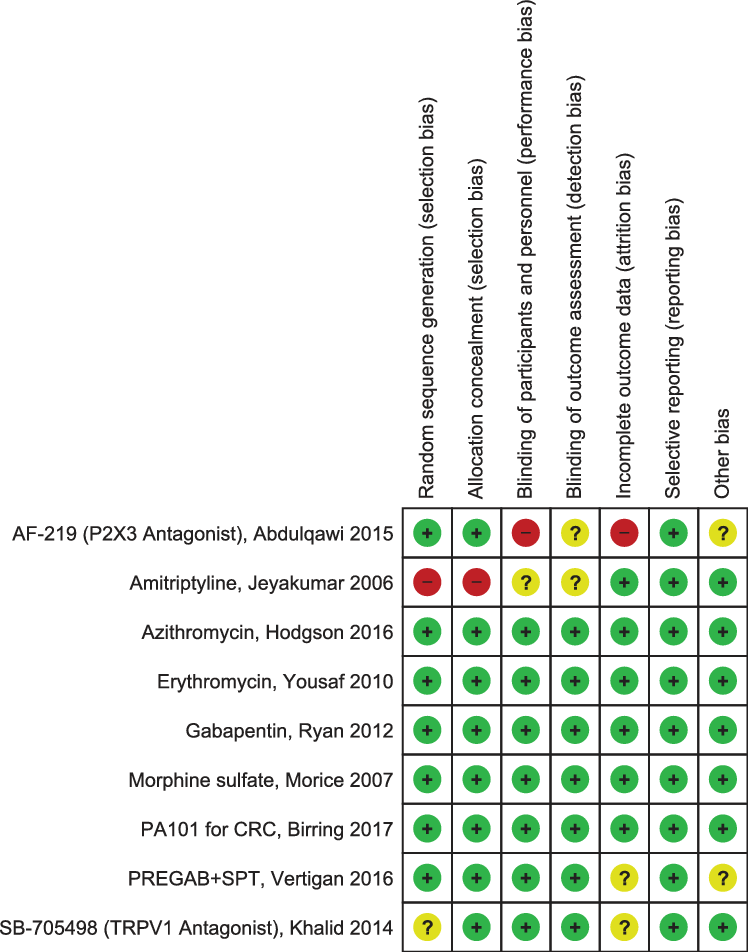 | 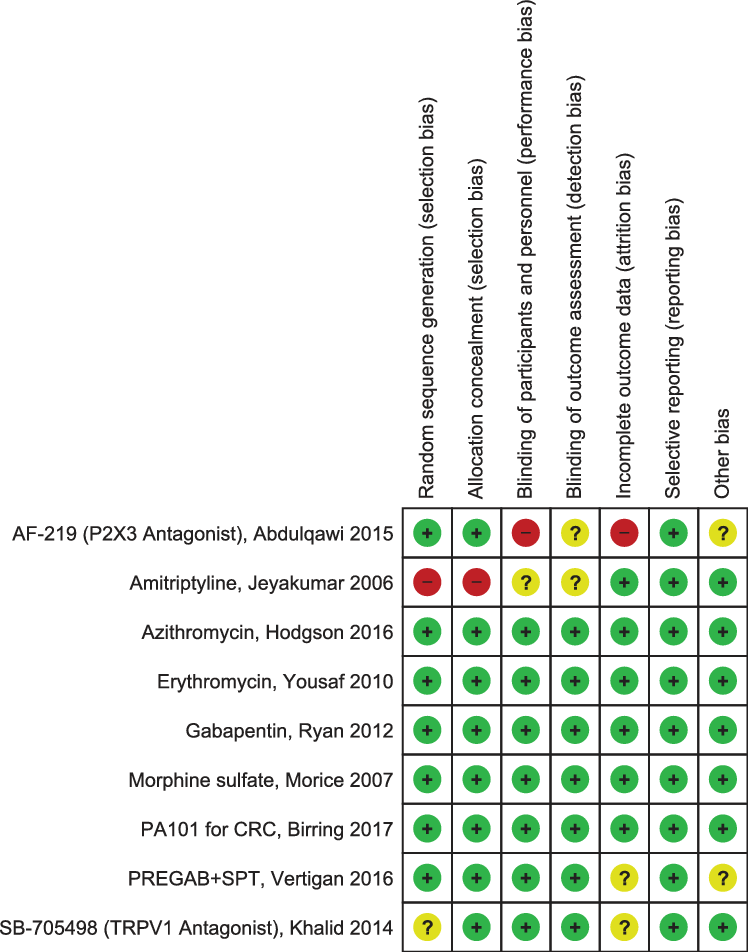 | 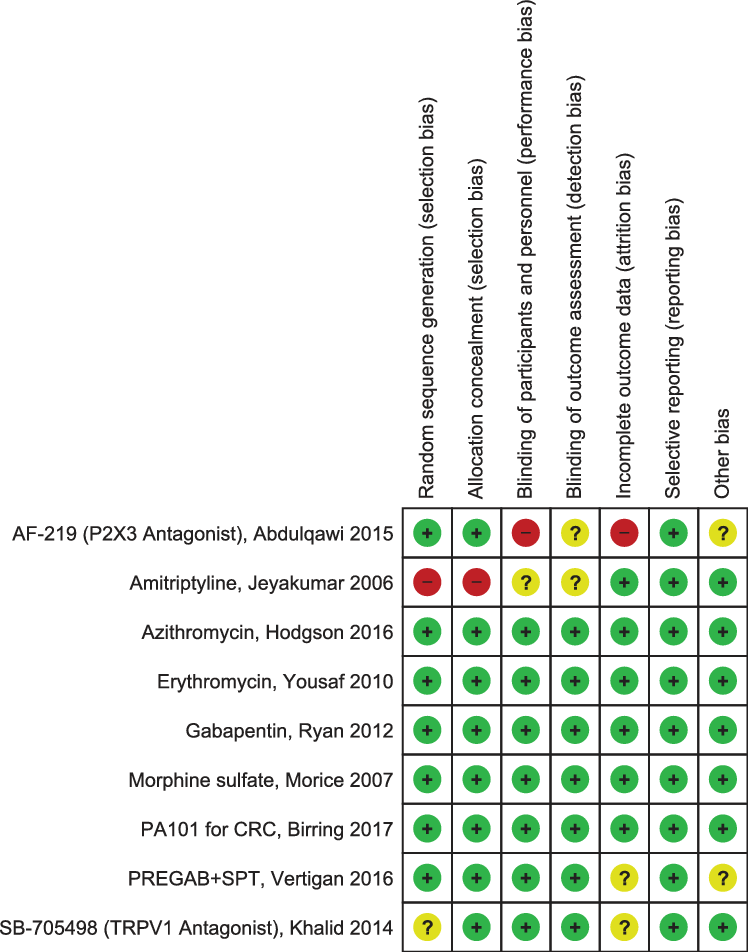 | 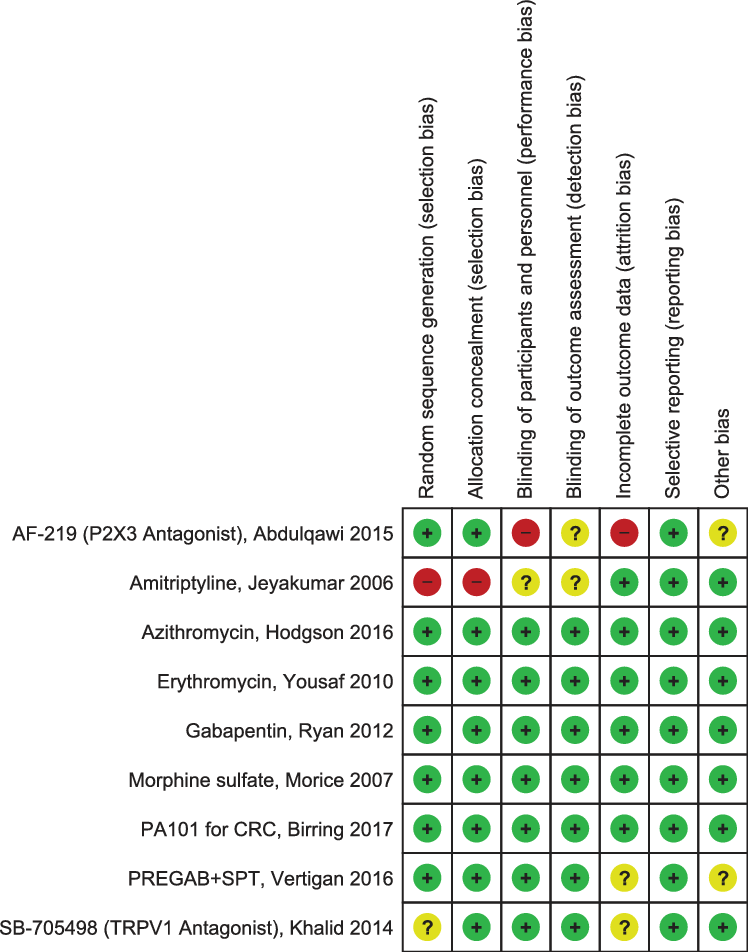 | 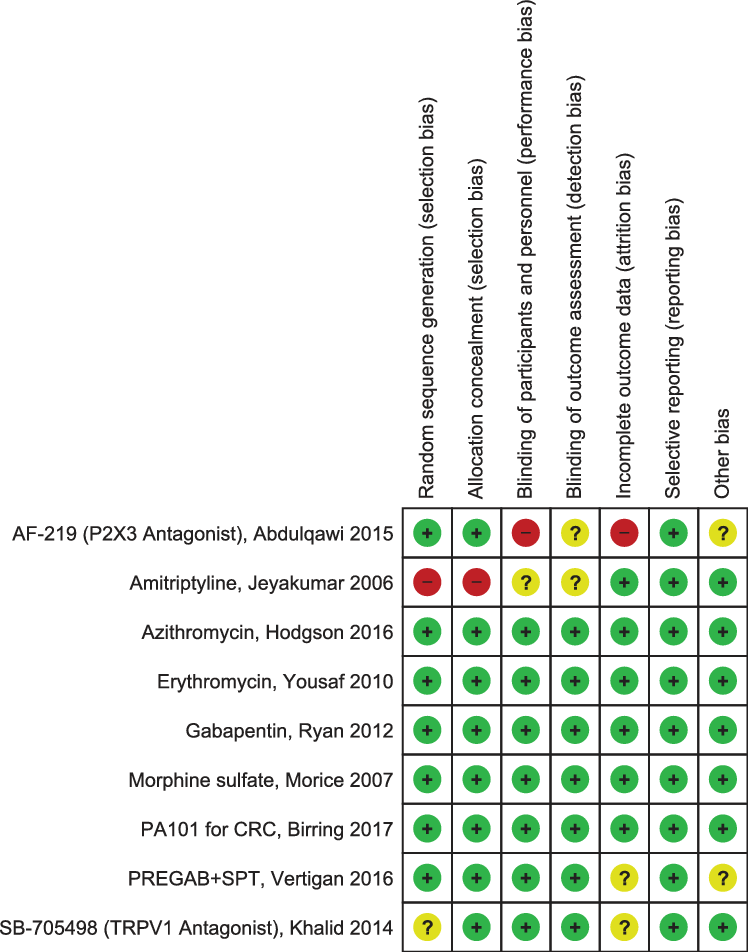 | 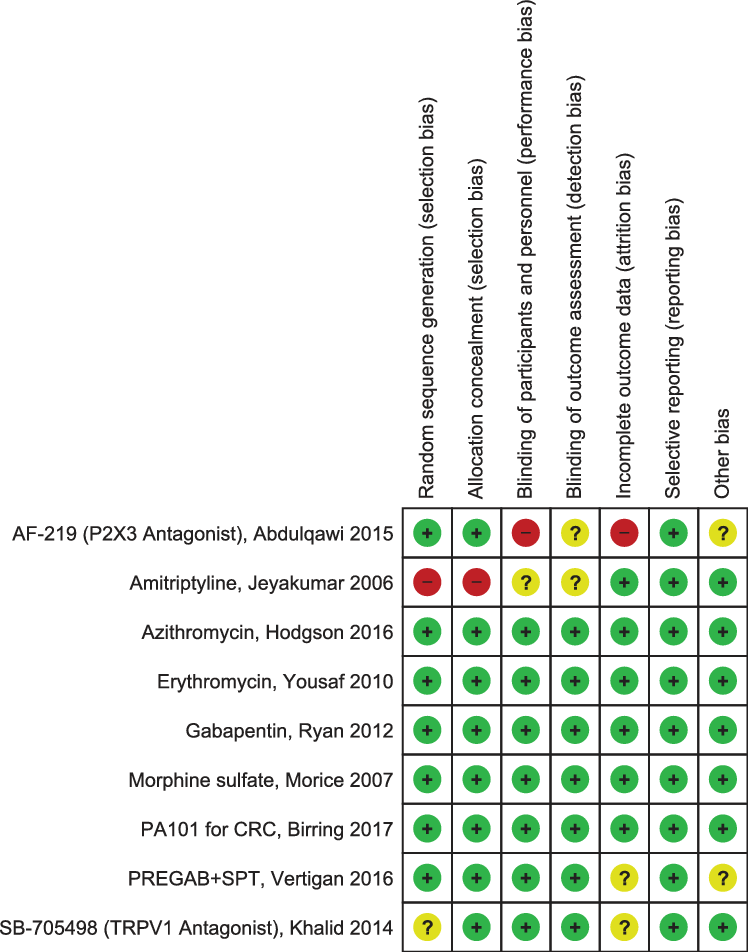 | 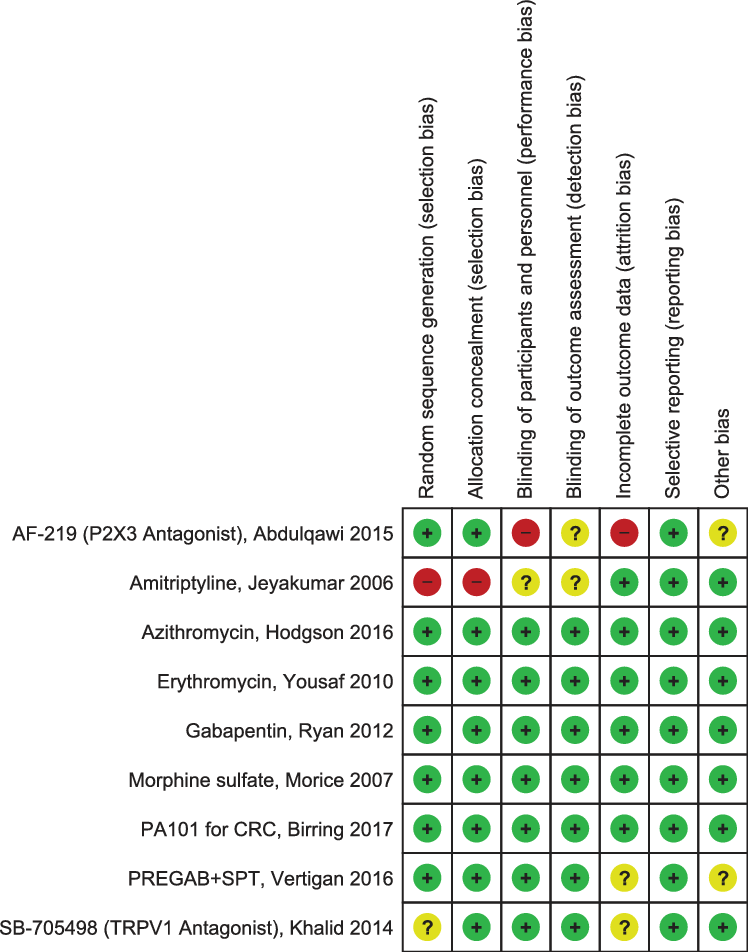 | 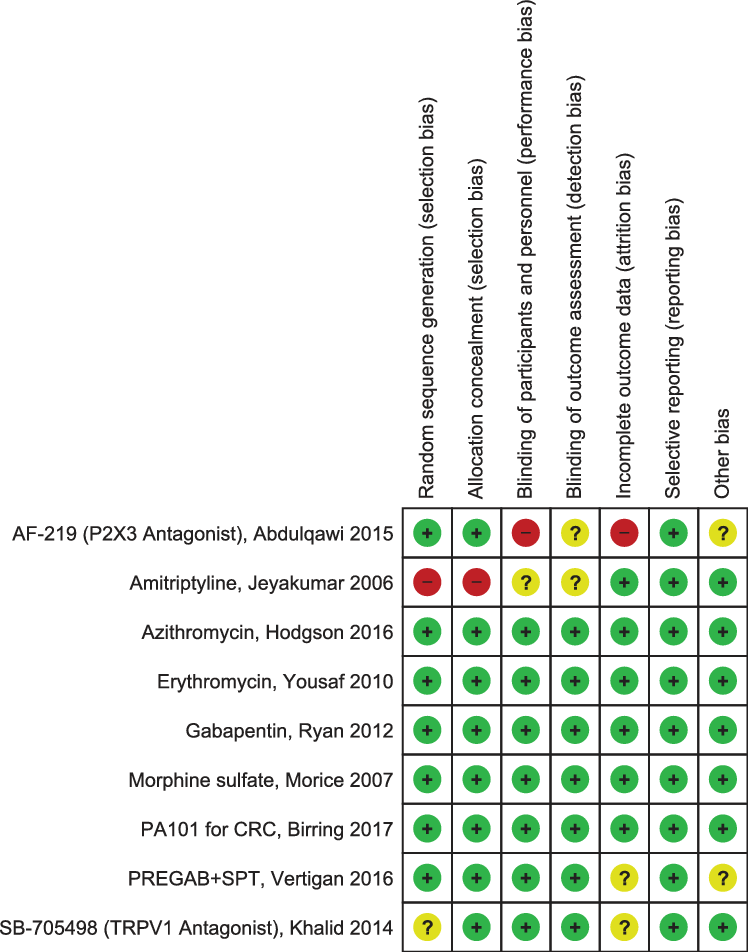 | 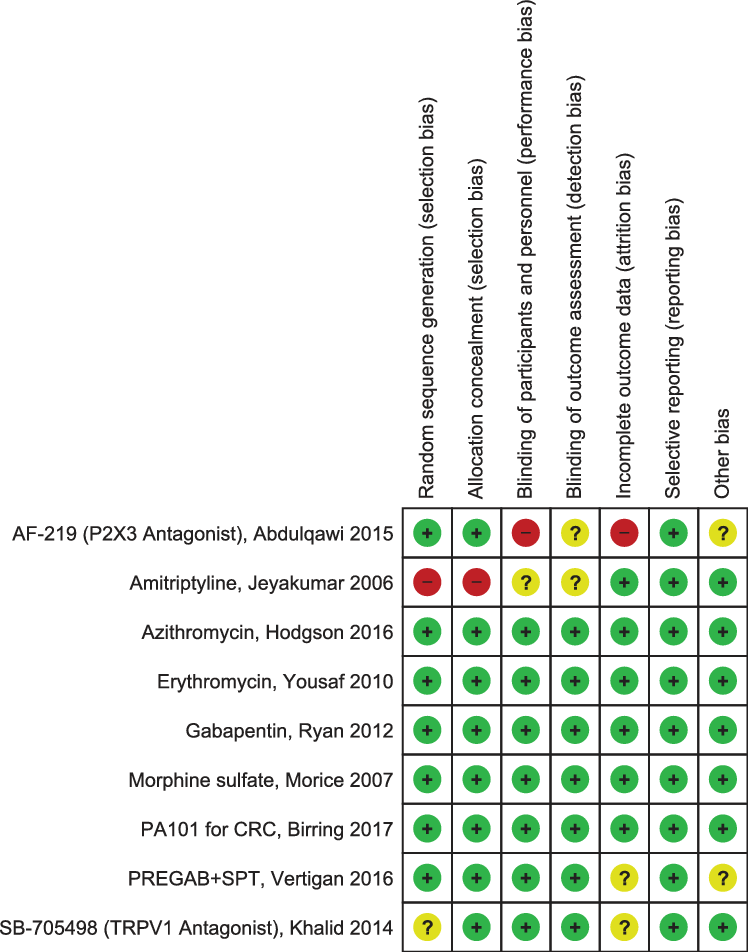 | 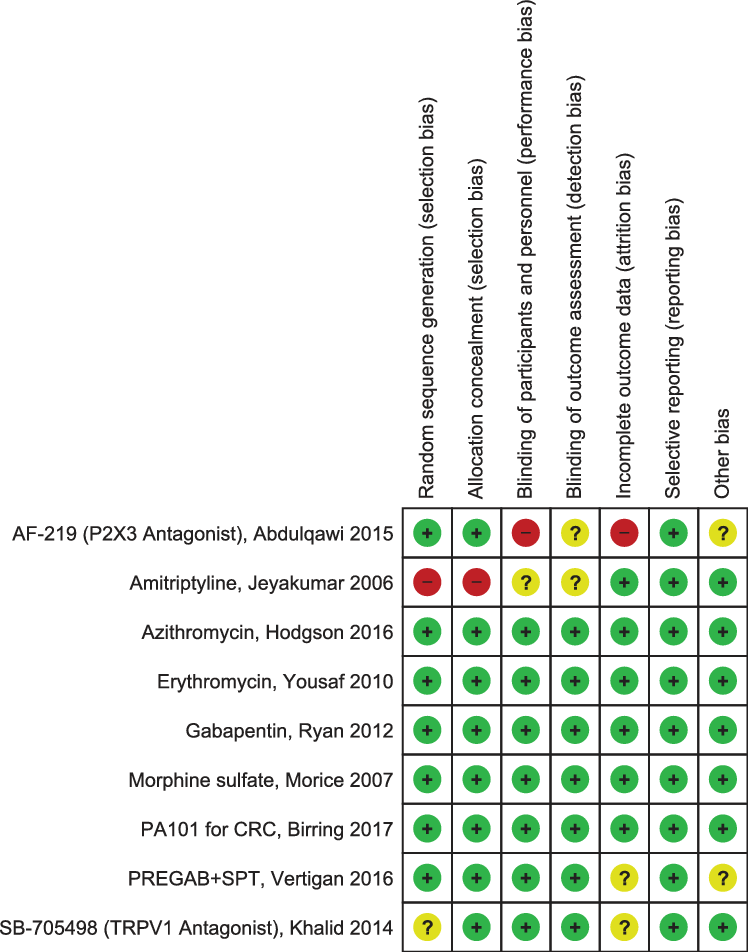 | 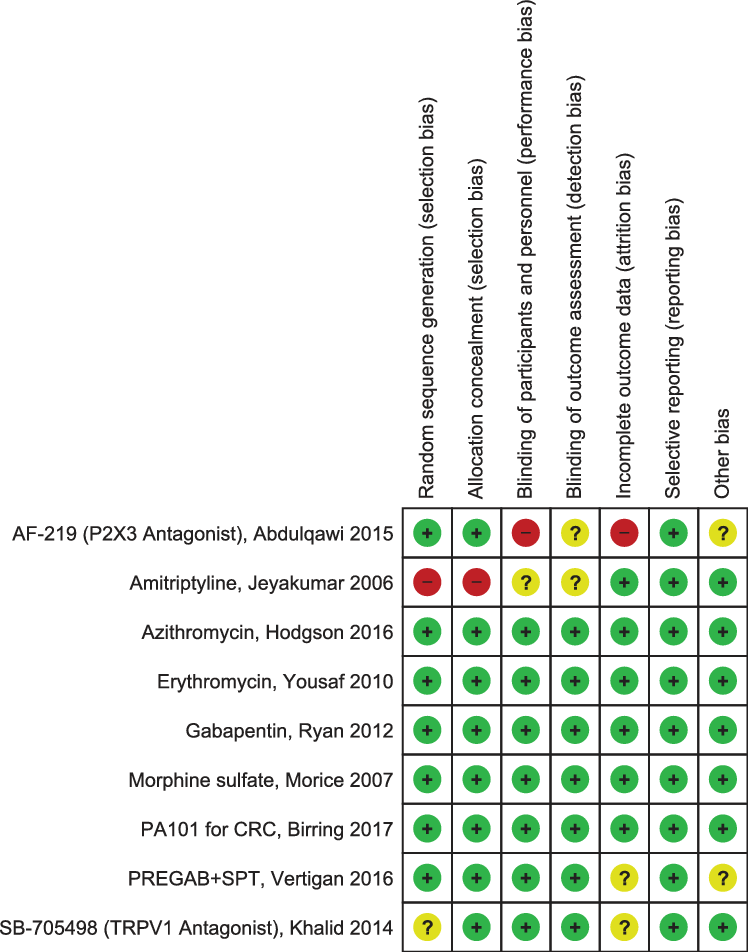 | 8 |
| Jiang et al., 2023 | RCT | 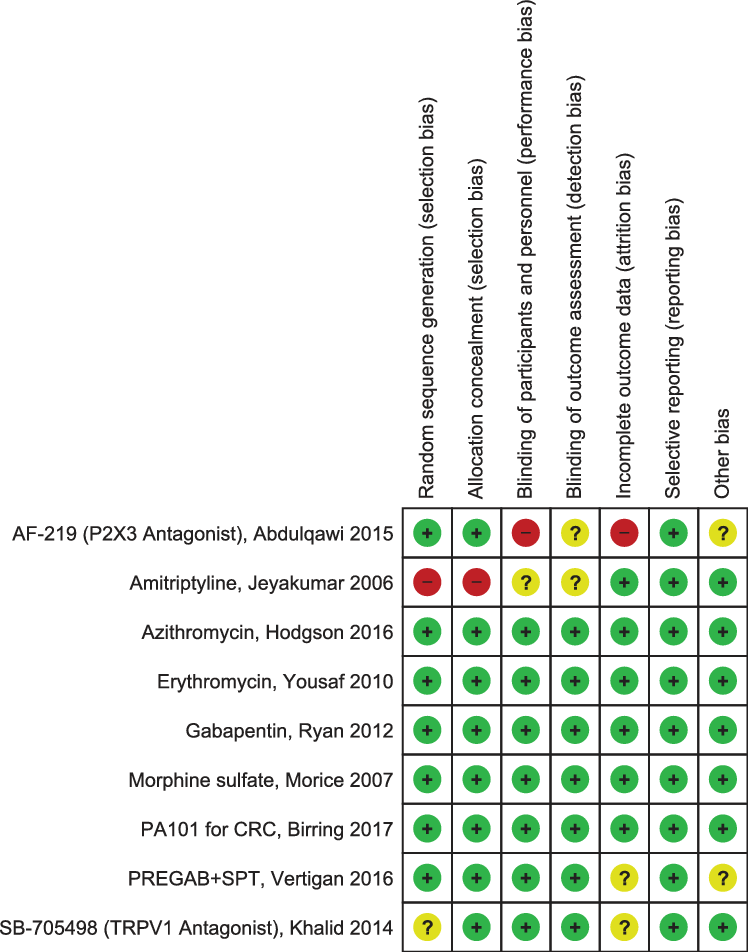 | 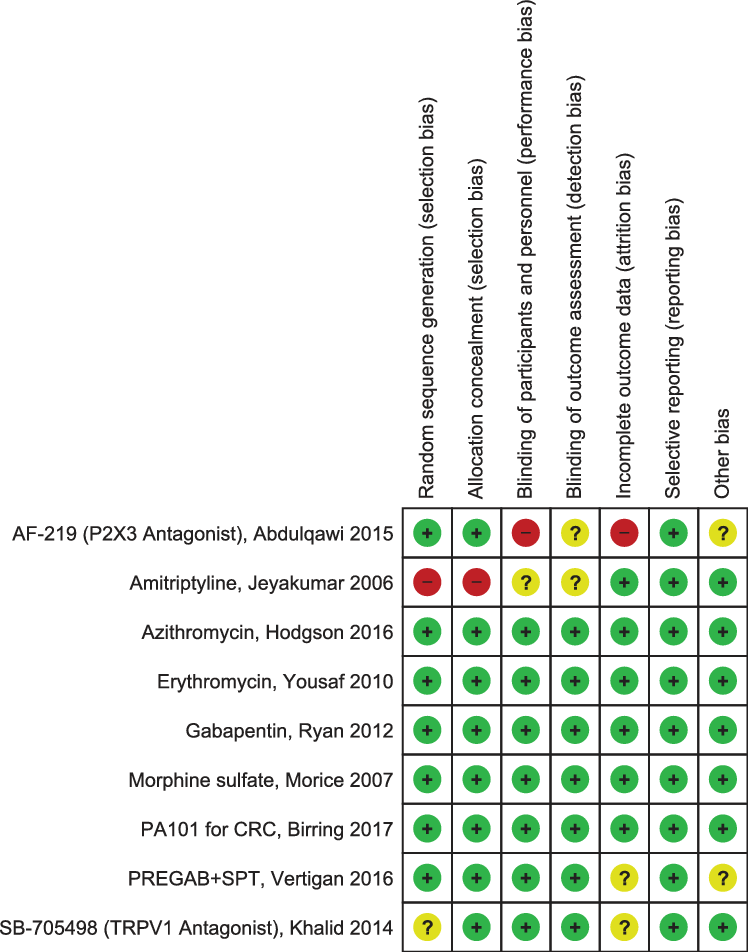 | 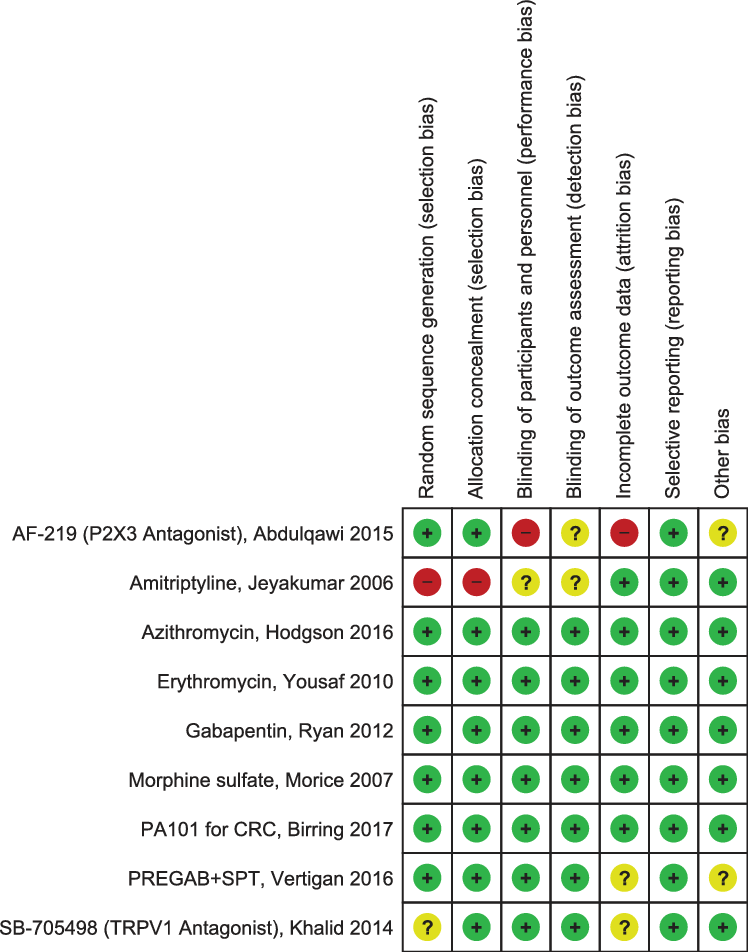 | 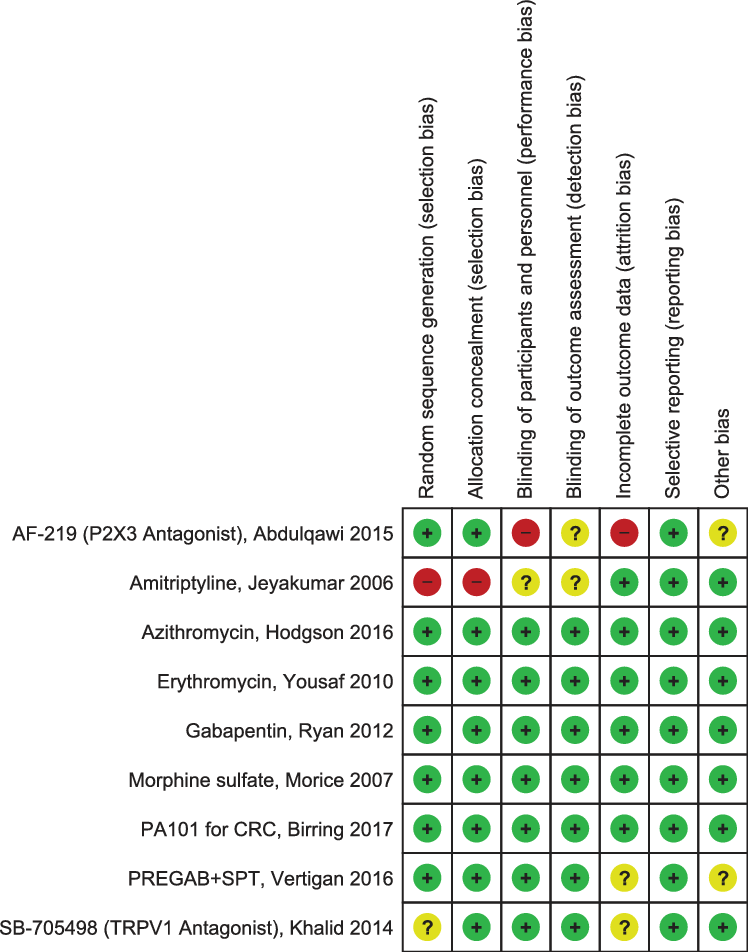 | 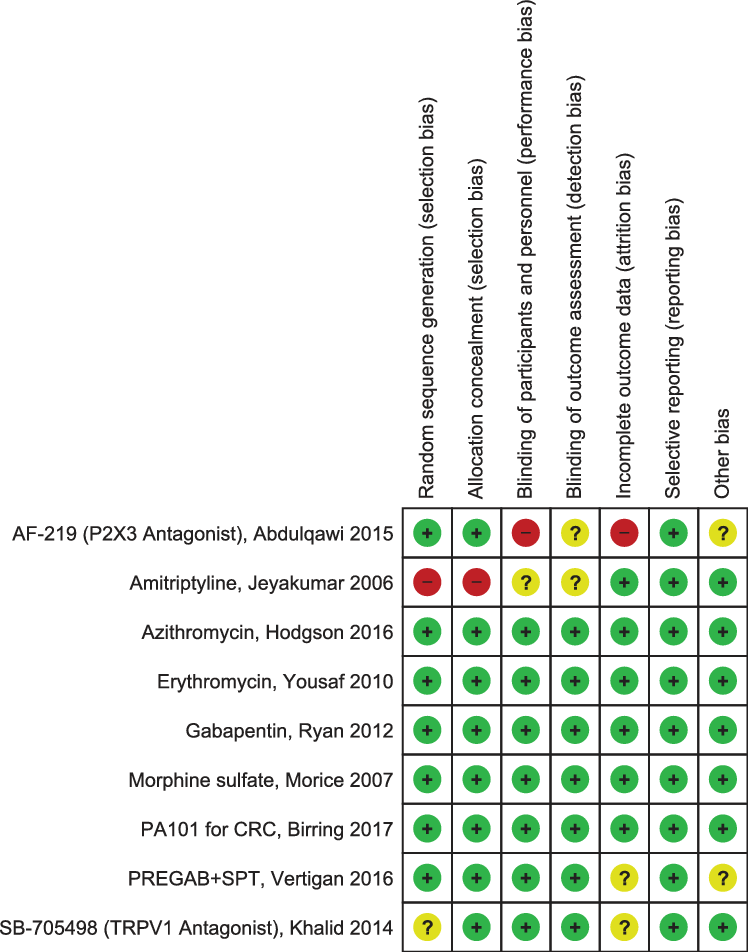 | 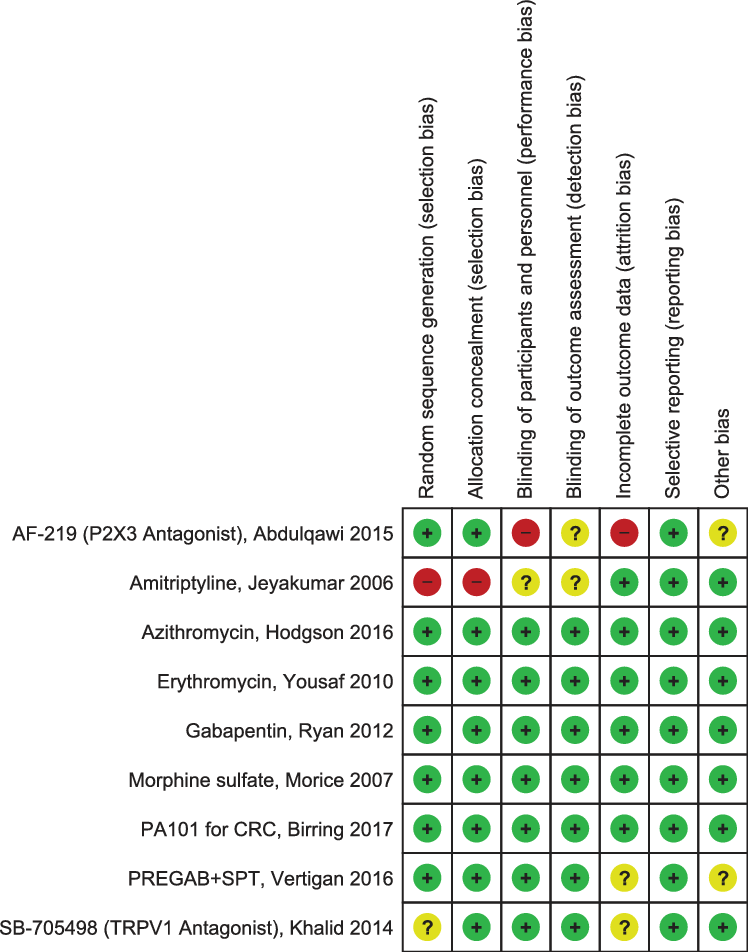 | 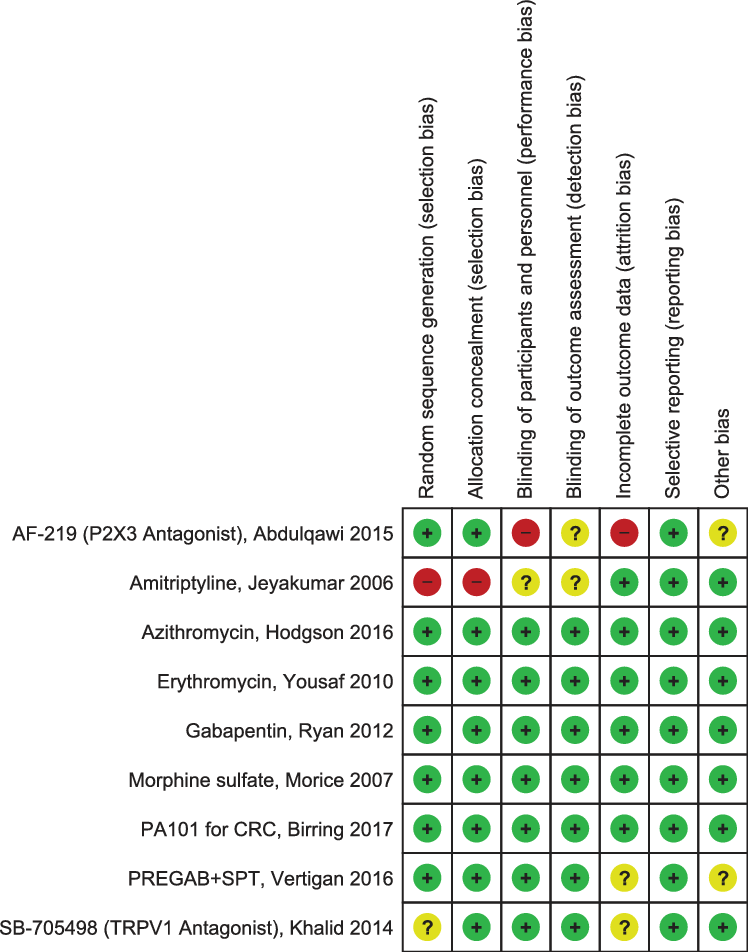 | 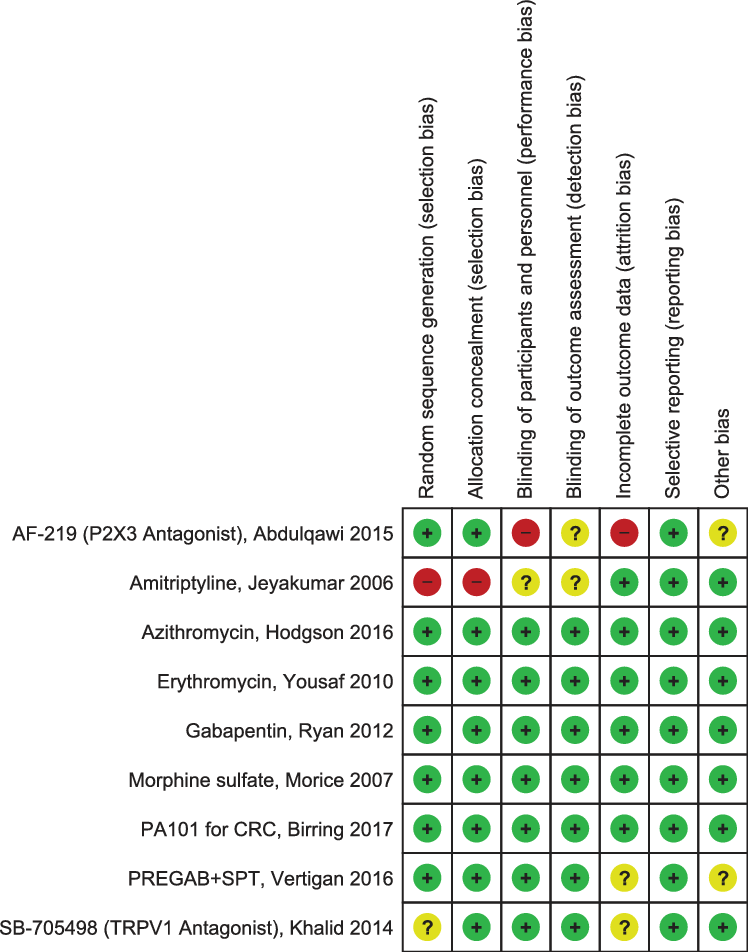 | 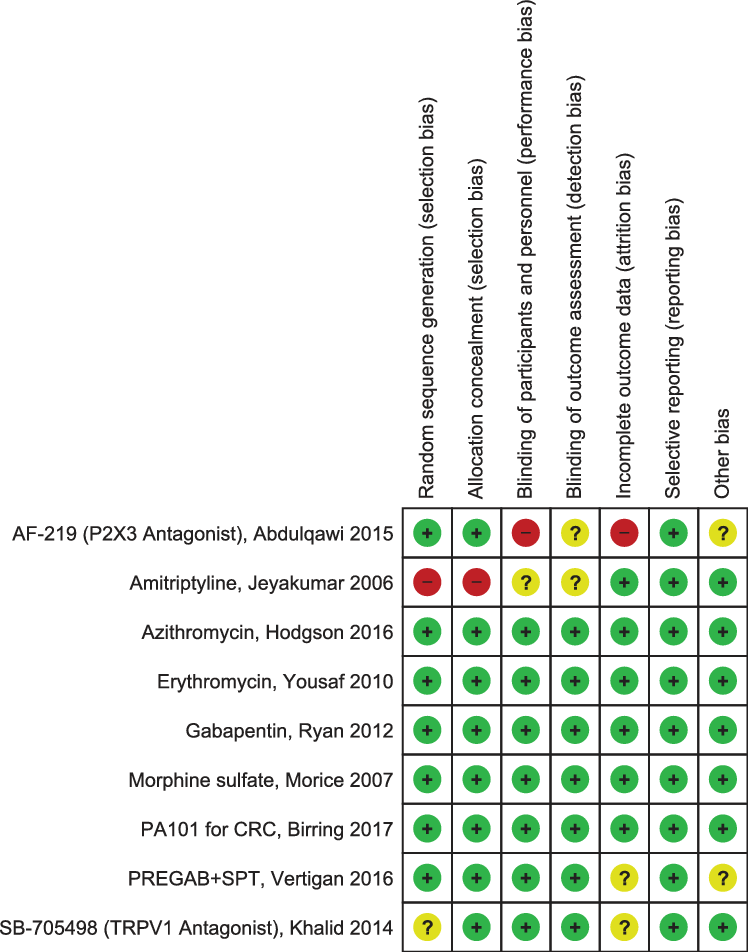 | 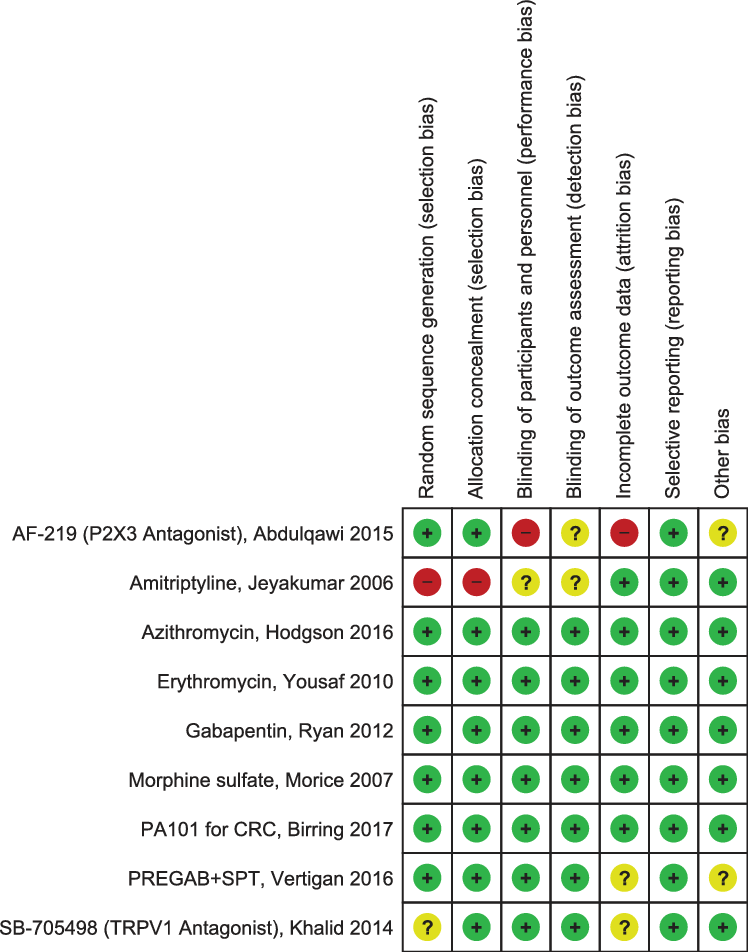 | 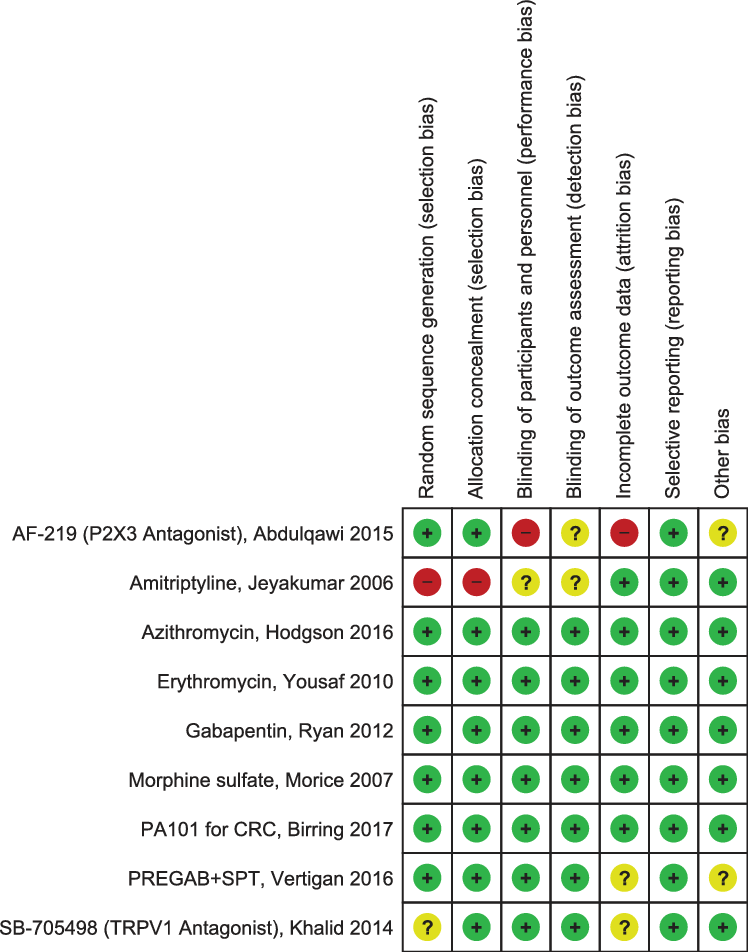 | 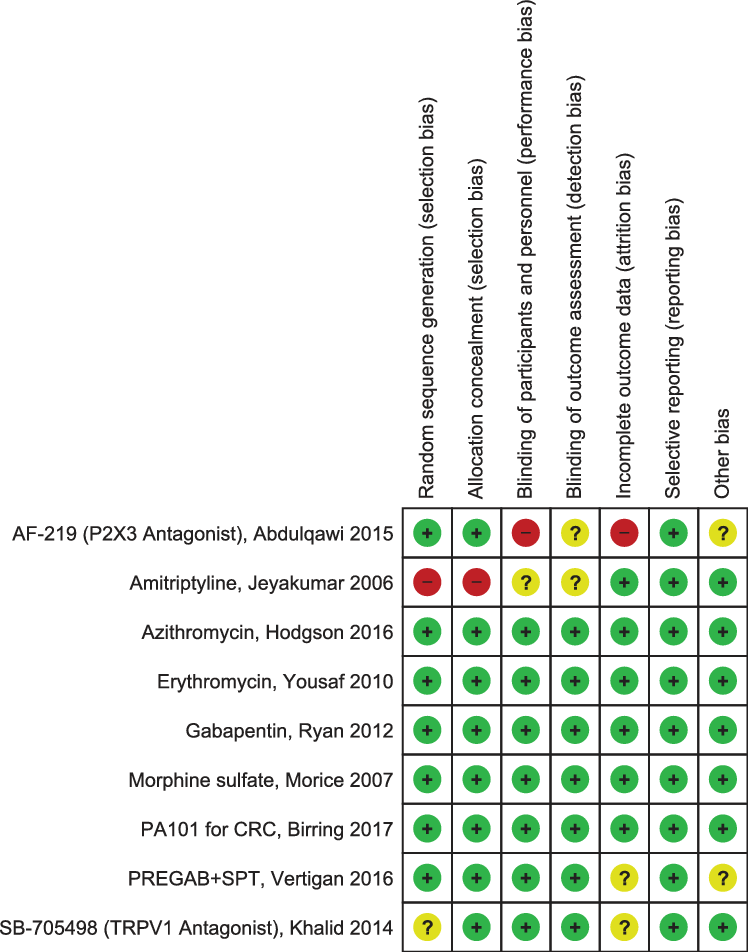 | 7 |
| Schofield et al., 2020 | RCT | 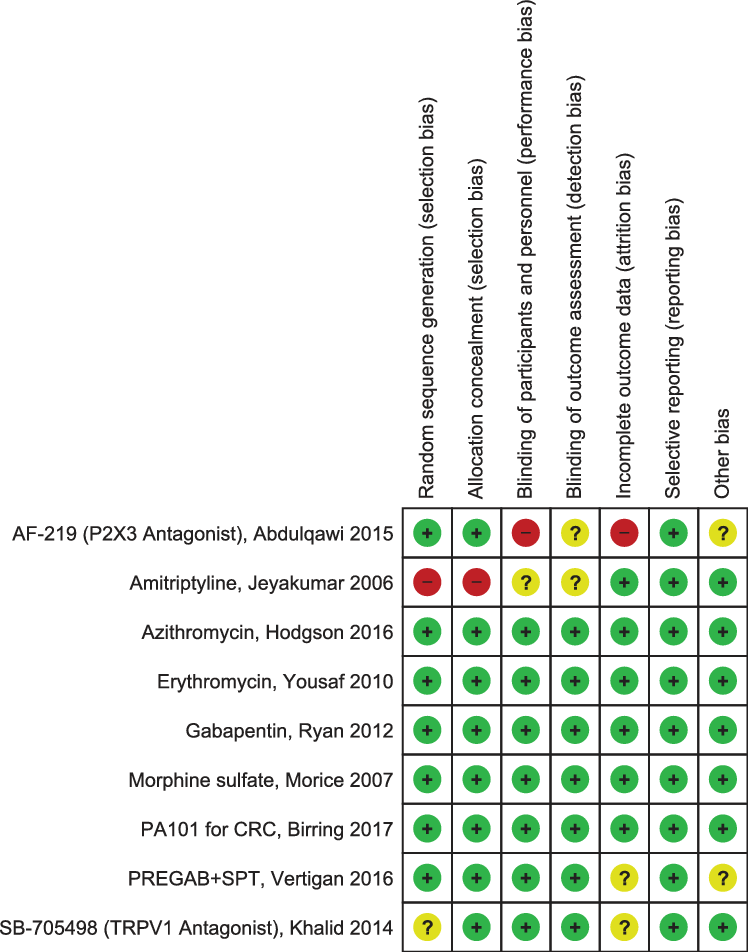 | 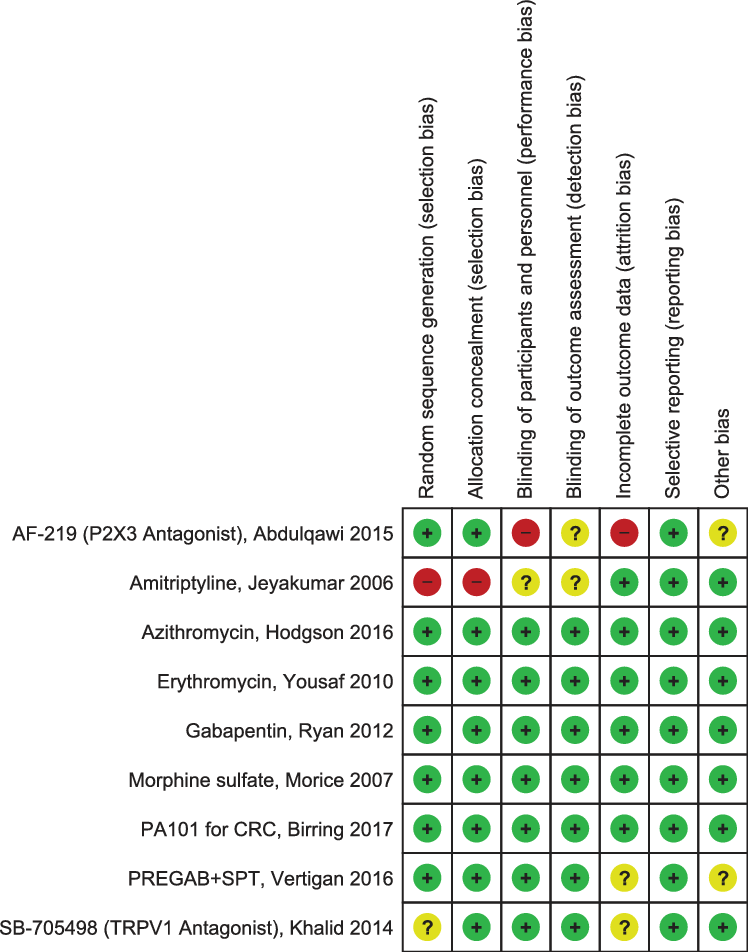 | 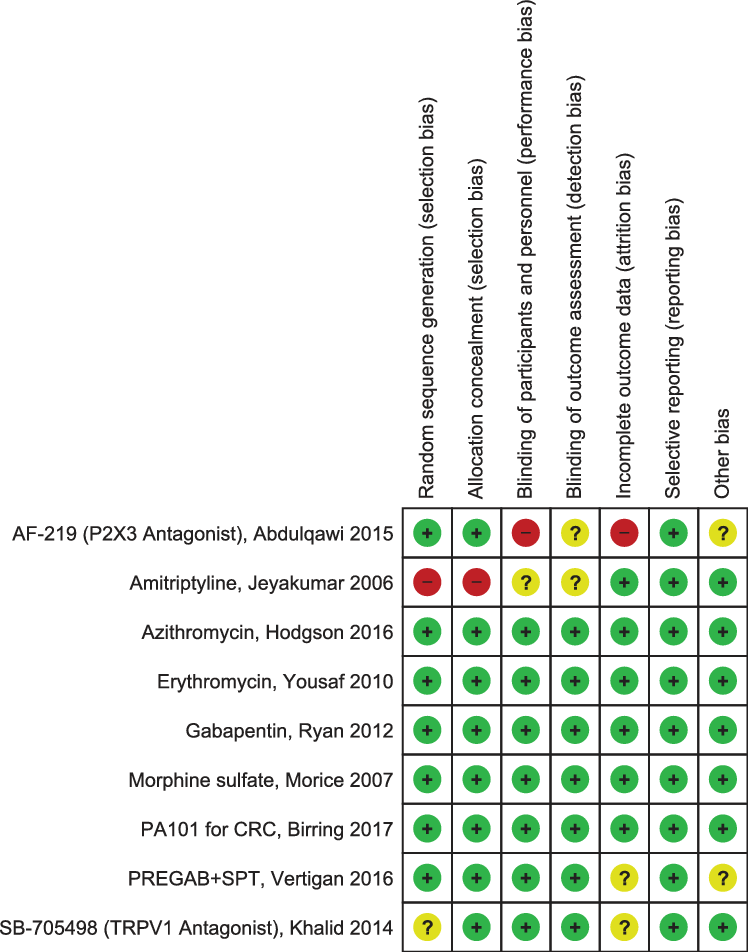 | 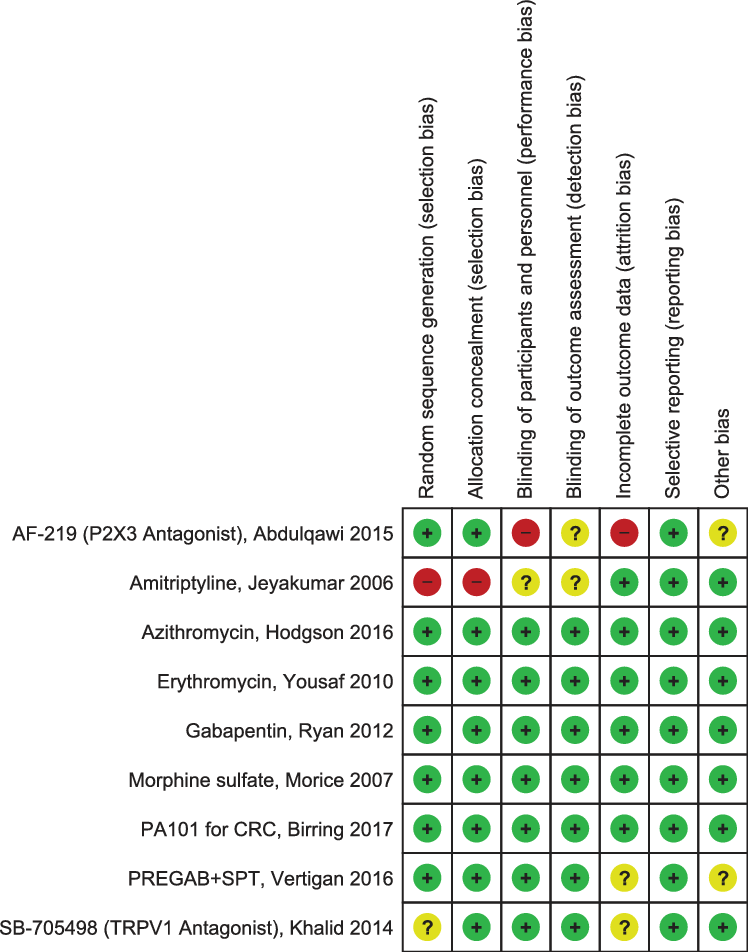 |  |  |  |  |  |  |  |  | 11 |
| Du et al., 2020 | RCT |  |  |  |  |  |  |  |  |  |  |  |  | 5 |
| Robinson et al., 1999 | RCT |  |  |  |  |  |  |  |  |  |  |  |  | 10 |
| Li et al., 2023 | Non-RCT |  |  |  |  |  |  |  |  |  |  |  |  | 6 |
| Tung et al., 2024 | Non-RCT |  |  |  |  |  |  |  |  |  |  |  |  | 9 |
| Cyr et al., 2020; 2021; 2022a; 2022b | Non-RCT |  |  |  |  |  |  |  |  |  |  |  |  | 11 |
| Brennen et al., 2023 | Non-RCT |  |  |  |  |  |  |  |  |  |  |  |  | 11 |
| Bernard et al., 2021 | Non-RCT |  |  |  |  |  |  |  |  |  |  |  |  | 11 |
| Sacomori et al., 2020 | Non-RCT |  |  |  |  |  |  |  |  |  |  |  |  | 10 |
| Bober et al., 2018; Hungr et al., 2020 | Non-RCT |  |  |  |  |  |  |  |  |  |  |  |  | 9 |
| Brotto et al., 2012 | Non-RCT |  |  |  |  |  |  |  |  |  |  |  |  | 6 |
| Brotto et al., 2008 | Non-RCT |  |  |  |  |  |  |  |  |  |  |  |  | 8 |

RCT: randomized controlled trial, TIDieR: Template for Intervention Description and Replication

(b) Detailed TIDieR Checklist

| **Author, year** | **Name of intervention** | **Rationale** | **Materials** | **Procedures** | **Provider** | **Mode of delivery** | **Location** | **Intervention dosage** | **Tailoring** | **Modifications** | **Planned intervention adherence & strategies for adherence** | **Actual intervention adherence** |
| --- | --- | --- | --- | --- | --- | --- | --- | --- | --- | --- | --- | --- |
| Sun et al., 2023 | Health education based on the integrated theory of health behavior change in pelvic floor rehabilitation | This study focused on the application of health education based on the integrated theory of health behavior change in the pelvic floor rehabilitation of patients with cervical cancer after radical surgery. This theory integrates the core ideas of health behavior change theory, social cognitive theory, self-regulation theory, and other theories and can help patients participate and change their health behavior. | Education manuals, health lectures, videos, WeChat application, commitment letter, vaginal weights (5 different sizes/weights) | *Pre-contemplation and contemplation:* Patients were taught about cervical cancer through education manuals, health lectures, videos and online teaching, and were informed of precautions for cervical cancer rehabilitation and PF exercise.  *Preparation stage:* Patients and their families generated rehabilitation plans. Patients signed commitment letters and set out their health management or post-op rehabilitation goals.  *Action stage:* Patients generated post-op rehabilitation plans and formulated feasible goals. Team members helped patients obtain timely health knowledge through the WeChat platform. PF exercises were divided into Kegel exercise and vaginal weightlifting.  Nursing staff supervised behavior and cognitive changes through telephone follow-ups (2 per month), home visits (1 per month), and communication in WeChat. | 2 gynecological (prepared a rehabilitation plan) + 1 psychotherapist (assessed psychological states of patients and provided intervention) + 1 therapist for PF rehabilitation (provided guidance on PF rehabilitation to nurses) + 4 gynecological specialist nurses (implemented the plan and made summaries)  All had a bachelor degree or above and had worked in their fields for at least 5 years. All were trained for 1 month in the integrated theory of health behavior change and in cervical cancer by means of lectures and on-line classes. | Individual and group (assuming health lectures, etc. are group-based)  F2F supervision (PF exercises and maintenance home visits), tele-health (maintenance phone follow-up), and web-based (WeChat) | Unclear  China - patients attended a University Hospital for cervical cancer treatment. Unclear whether in-person sessions were in hospital / clinic / at home. | Unclear for intervention duration and number of sessions delivered.   Kegel exercises: Patients could perform exercise for 15-20 min per day. Beginners squeezed their PF for 3 sec, and then released their muscles for 5 sec before repeating the exercise. After a few exercise sessions, patients extended their squeezing time to 5 sec and released their muscles for 5-10 sec.   Vaginal weightlifting: patient held the dumbbell for 3-5 sec and relaxed for 8-10 sec. The patients repeated this process 20 times as a set and performed 3 sets per day. | Individually tailored (exercises, rehabilitation plan, WeChat support etc.) and generic (health lectures and videos) | NR | Unclear  Treatment compliance: This was divided into complete compliance, partial compliance, and non-compliance. Complete compliance: patient will actively cooperate with the treatment, took drugs punctually, and accepted all related examinations. Partial compliance: patient cooperated with the treatment, took drugs and accepted related examinations. Non-compliance: patient did not cooperate with the treatment and quit the study. Treatment compliance = (complete compliance + partial compliance)/number. | Unclear  Treatment compliance = 95.4% Complete compliance=56.9% Partial compliance=26.2% Non-compliance=16.9% |
| Zong et al., 2022 | Kegel PFM training combined with clean intermittent self-catheterization | It has been observed that PFM exercise boasts various benefits such as improving PFM contraction and diastolic tension, enhancing urinary continence capability, and accelerating the recovery of bladder function. There are few reports on the combined effect of PFM exercise and clean intermittent self-catheterization in patients with cervical cancer after surgery. | Catheter. Nil exercise materials. | Patients were instructed on the procedure of Kegel PFM exercises 3 days pre-surgery, and diastolic and contractile exercises of the vagina, urethra and anal sphincter were performed on day 4 post-surgery while lying in bed.  Patients were supine with legs flexed apart. When inhaling, the perineum and anus were contracted forcibly, lasting about 10 sec, and when exhaling, relaxed about 10-sec. The above actions were repeated for 20 min, with an interval of 5-10 sec between each set, three times a day.  Self-catheterization: The time interval of urethral catheterization was set according to patients’ residual urine volume, usually 4-6 hours and 4-6 times a day.  After discharge, regular telephone follow-up was conducted to 14 days post-surgery. | Nurse provider | Unclear  Not explicitly stated but assuming individual F2F in hospital.  Then individual telephone for follow-up (14 days post-surgery). | Unclear   China - patients attending Hospital for radical resection of cervical cancer. Not explicitly stated but assuming session took place in hospital until discharge - though participants did not always remain in hospital until Day 14. | Kegel exercises were prescribed 3 times per day (20 min each) from day 4-14 post-surgery.  Self-catheterization: 4-6 times per day. | Generic | NR | NR | NR |
| Li et al., 2019 | Traditional bladder function training and low frequency electrical stimulation | Some scholars have begun to apply electrical stimulation for post-op rehabilitation of malignant tumors, effectively improving quality of life. At present, there are few studies on the rehabilitation treatment of urinary retention electrical stimulation after cervical cancer surgery. | Catheter, Phenix VBS neuromuscular stimulation therapy system | From day 11 post-surgery, patients were given electrical stimulation treatment and bladder function training for 3 days. The patient was placed in a supine position, one piece of the electrode was placed in the bladder area under the symphysis pubis, and the other piece was placed on the bone.  Two sets of different parameters were chosen according to different treatment principles: electrical stimulation frequency was 35Hz and pulse width was 200μs (neuromuscular repair program, group A) or frequency was 1Hz and pulse width was 270 μs (endorphins analgesia program, groupB).  Both groups underwent bladder function training: From day 11 post-surgery, between 8am and 10pm, the catheter was opened every 2 hours for 15 min each time, while the catheter was fully opened from 10pm to 8am the next day, until the catheter was pulled out. | NR | Unclear  Can assume individual delivery and F2F supervision, though this is not clearly reported. | Unclear  China - patients attending Hospital for cervical cancer. Not explicitly stated but assuming session took place in hospital until discharge. | Electrical stimulation: 15-30 min each, 2 times per day, for 3 days.   Bladder function training: From day 11 post-surgery, between 8am and 10pm, the catheter was opened every 2 hours for 15 min each time, while the catheter was fully opened from 10pm to 8am the next day, until the catheter was pulled out. The catheters were removed from all patients at 14 days after surgery. | Generic | NR | NR | NR |
| Li et al., 2016 | Home-based, nurse-led health program | Home-based nursing intervention (HBNI) is a nursing program that extends nursing intervention from the hospital to the home in China. Research suggests that HBNI could assist patients in managing adverse effects more effectively than standard care. Research also suggests specific exercises at home are effective interventions for older patients. Taken together, it seemed possible that yoga may have a beneficial impact on cancer survivors. All home nursing care research involves special nursing interventions according to the characteristics of the disease. However, to our knowledge, no studies have applied HBNI in post-op patients with early-stage cervical cancer. | Instructional yoga CD | The NLHP-HB program consisted of 5 concepts  *Establishment of a family care team: a* specialist nurse served as the leader and was responsible for nursing education, promoting the program, collecting scales, and carrying out the follow-up. Other team members provided consultation and advice based on their respective expertise. *Physiological rehab*: standard Kegel exercises, for PFM training were introduced. PFM training began by emptying the bladder, and contracting the PFM for 10 sec, followed by complete relaxation of the muscles for 10 sec. 10 reps, 3-5 times per day were suggested. Patients should breathe deeply and relax their bodies when doing these exercises. The training method was explained and taught before patients left the hospital. *Emotion-release management*: a simple yoga exercise was introduced for this study and comprised a warm-up exercise (shrugging, head waving and breathing simultaneously); suitable physical stretches and postures (Child’s Pose, Cobbler’s Pose, Staff Pose, Triangle Pose, Warrior Pose, Mountain Pose, and Downward Facing Dog); deep relaxation (yogic conscious deep-sleep in supine posture); single-nostril breathing alternately (left-to-right nostril breathing for eight seconds alternately); and meditation (concentration, mindfulness, and contemplation). 30 min, 2 times per day were recommended. Everyone was offered an instructional CD that contained the yoga training video before leaving the hospital. *Informal social support*: informal social support systems consisted of surrounding family and friends. We enhanced the education through the informal social support system. We explained the disease, alleviated worry and fear, and encouraged giving more support. To encourage each other, we built a communication between new patients and those who received a successful NLHP-HB program. *Home follow-up monitoring*: an online communication platform was implemented to solve questions and give advice. A telephone follow-up was carried out every 2 weeks, and a home visit every 2-3 months was available with the permission of the patients. Some education and questionnaires were finished when patients returned to visit doctors in the outpatient department. | Family care team: consisting of specialist nurses, gynecological doctors, physiotherapists, psychological consultants, dietitians and physical therapists.  NR for PFM training.  Yoga: Researchers received ‘amateur training’. | Initial training supervised F2F at the hospital (assume individual). Follow-up unclear.   Follow-up: online communication platform and telephone. F2F home visit every 2-3 months was also available. | Initial training: F2F at hospital (assume individual).  Follow-up: online, telephone, and patient home. | PFM training home program: 3-5 times per day, 10 reps of contraction for 10 sec and relaxation for 10 sec.   Duration of exercise home program not specifically reported. “Intervention took 6 months”.  Yoga – 30 min, 2 times per day. | Generic | NR | NR | NR |
| Rutledge et al., 2014 | PFM exercise training | Despite proven effectiveness in the general female population, the effectiveness of PFM training and behavioral therapy, both being simple interventions, has not been evaluated in gynecologic cancer survivors. The effectiveness of these interventions may differ in cancer survivors since radiation, chemotherapy and radical pelvic surgery can result in significant anatomical functional changes in the pelvis and lower urinary tract, including damage of nerve fibers and compromise of vascular supply with resultant fibrosis. Data are lacking evaluating treatment options for gynecologic cancer patients who are incontinent. | A handout and instruction describing behavioral management tips for UI. | The provider conducted a training session during the clinic visit designed to teach the participant to contract PFM correctly. The training session required ~15 min. The PFM training program was explained to the participant verbally and in written form. The training program consisted of the participant performing 10 PFM contractions with a goal of holding the contraction for 5 sec; women were asked to perform 3 sets daily for the 12-week study period. | A ‘provider’ who attended 2 therapy sessions with experienced PF physical therapists. | Individual., Initial training supervised F2F, then 1 follow-up reminder phone call. | Clinic (Department of Obstetrics and Gynecology, University of New Mexico) and home program. | 1 x initial training session (15 min), PFM training for 12 weeks - 10 PFM contractions, 3 sets daily. | Generic | NR | To promote adherence, participants received a reminder phone call approximately 4 weeks after the first study visit. The phone call reviewed the training instructions and addressed concerns or questions the participant had.  12 weeks after randomization, participants returned for the second study visit. At this visit, participants completed questions regarding treatment compliance such as how many exercises they performed per day, and how well they complied with the exercise program. | Women in the treatment group judged their compliance with the exercise therapy to be ‘excellent’ or ‘good’ in 75% of cases, ‘poor’ in 20%, while in the remaining 5% the exercises were not performed at all. |
| Yang et al., 2012 | Pelvic floor rehabilitation program | Recent research has suggested the importance of synergy between the abdominal muscles and PFM. Based on this understanding, we developed a PF rehabilitation program to strengthen PFM utilizing trunk stabilization. By using abdominal muscle action to initiate tonic PFM activity and by monitoring biofeedback, patients are made aware of the contractions and can incorporate PFM training into their daily activities. | Vaginal pressure biofeedback and rectus abdominus surface electromyography biofeedback, exercise diaries, leaflets. | Patients were invited to attend a PF rehabilitation program, consisting of one 45-min exercise session and one 30-min counselling session per week for 4 weeks.  The exercise program comprised biofeedback sessions and core exercise sessions. Diaphragmatic breathing techniques were taught as an important part of the core-strengthening program.  Each session included a 30-min comprehensive counselling session consisting of all components including PFM evaluation, lifestyle advice, and encouragement and re-education of home-based PF exercises in addition to the exercise program.  Home Program: Patients were taught in detail about the methods of constricting their PF muscles, with special emphasis on avoiding constricting the abdominal muscles simultaneously. | Experienced physiotherapist; physical therapist | Supervised weekly F2F sessions of PFM training and strength training. Assume individual., | Hospital (Seoul National University Bundang Hospital, Korea). | Biofeedback: 20 min and consisted of 40 cycles with 10 sec of activity followed by 20 sec of relaxation.  Core exercise: After a 5-min rest period, patients received 20 min of an intensive core exercise session consisting of strengthening exercises for the PFM and transverse abdominis muscles and stretching exercises for muscles attached to the pelvic girdle such as the gluteus, tensor fascia latae, piriformis, and adductors and the surrounding ligaments. Diaphragmatic breathing techniques were taught as an important part of the core-strengthening program.  Home program: 6 sets of exercises daily. Each set consisted of 10 maximum voluntary contractions held for up to 10 sec, with a 4-sec rest between contractions, and followed by 1-min pause and 10 or more fast contractions for 20-30 sec. The goal was to perform exercise even in the upright position. | The content of the home exercise program was adjusted according to subjects’ circumstances. | NR | NR | NR |
| Cerentini et al., 2019 | Vaginal dilators during and after brachytherapy | Aiming to minimize the effects that the radiotherapy treatment promotes on the vaginal dimensions, vaginal dilators have been widely used. However, their use is limited by psychological resistance. In addition, there is still a lack of evidence demonstrating the benefits of using this technique on vaginal dimensions, PF functionality, clinical signs and symptoms, and quality of life. The aim of this study was to evaluate the dimensions of the vaginal canal in patients undergoing gynecological brachytherapy and the effect of the use of vaginal dilators used in the follow-up of pelvic physiotherapy. | Sos vaginal dilators (MDTi Company) and guidelines for its use. | The vaginal dilators were given to patients following the stipulation of vaginal size on the first evaluation. The use of vaginal dilators was individual, and patients were oriented to use the device for 3 months, four times per week for 10-15 min each time. | Trained physiotherapist | Individual, unsupervised | Unclear  Assume in-clinic for assessment for dilator size, then presumably at home, though this is not clearly reported. | Use vaginal dilators for 3 months, 4 times per week, for 10-15 min. | Individually sized vaginal dilator compatible with the participants individual anatomical conditions. | NR | NR | NR   Adherence to the protocol declined with follow-up and there was a high rate of loss throughout the study but this behavior was similar between the groups. Results of multivariate analyses showed that adherence was not significantly influenced by the variables. However, in women with hypoactive PF, there was a tendency for less adherence to follow-up (p = 0.070). |
| Jiang et al., 2023 | Routine nursing care and continuing nursing | The continuous implementation of study on the symptom cluster of cervical cancer patients, the discussion on the influencing factors of symptom cluster, and the compliance with the new trends in cancer management show a profound influence of improving quality of life of cervical cancer patients and promoting female health. | NR | WeChat platform was set up for online education with telephone follow-up. Small psychological guidance seminars, and outpatient follow-ups were adopted as the auxiliary methods to implement continuing nursing intervention. After the seminar, one-to-one guidance was offered for couples accordingly. In addition, patients and their husbands were invited to back to the hospital to participate in the seminars on various relevant knowledge. | Gynecologic surgical physician-in-charge (cervical cancer specialized knowledge training) + Nurses with cervical cancer care experience (clarification of the relevant procedures, common problems, countermeasures of continuing nursing) + Psychiatrist (psychological counselling skills training) + Psychological consultant (psychological counselling skills training) + Rehabilitation therapists (guidance on functional training) + Postgraduates (understanding of the significance of continuing nursing and mastery of relevant skills and knowledge) | Online education: individual and web-based. Seminar: group and F2F. | Unclear  Seminars: at the hospital. Assume West China Second University Hospital, Sichuan University but not explicitly stated. | Unclear  Each seminar lasted ~20-min; intervention duration 3-6 months. | Unclear | NR | Interveners checked the exercise of patients every day and counselled patients who raised questions. After discharge, intervention implementers reminded the patients in the intervention group to carry out PF muscle rehabilitation training by WeChat and telephone follow-up and check the effects of exercise. In addition, they needed to answer the questions and confusions patients were confronted with in time through individual chat on WeChat to enhance the compliance of patients in the experimental group. | The exercise frequency of PFM among patients in the two groups was compared, and the results showed that the differences between the two groups demonstrated statistical significance (P<0.01) (direction is unclear but those with highest frequency are in TG). |
| Schofield et al., 2020 | Psycho-education nurse- and peer-led psycho-educational intervention | Peer-support can increase patient confidence and hope, and help patients make sense of their illness/treatment/recovery experience. Therefore, the aim of this intervention was to evaluate the impact of a combined nurse- and peer- led psycho-educational intervention. | Intervention manual, vaginal dilator, survivorship care plan. | Nurse-led consultations: delivered at 3-4 timepoints (pre-treatment, mid-treatment [not for brachytherapy-alone patients], treatment completion, and 2-4 weeks post-treatment). *Session 1*: Patients had a radiation facility tour followed by a consultation to address their top three concerns prior to radiotherapy.  *Session 2*: Side-effects of radiotherapy were discussed and use of the vaginal dilator and performance of PFM exercises demonstrated.  *Session 3*: Patient’s concerns about treatment completion, side-effects and psycho-sexual recovery were discussed and their Survivorship Care Plan completed.  *Session 4*: Patient’s concerns were explored, barriers to self-care strategies addressed and the importance of dilator use reinforced.  Peer telephone support: Trained peers were matched to the patient’s diagnosis, treatment modality and age. The peer was provided with the patient’s: first name, basic demographic details, main concerns, and nurse-recommended self-care strategies. Peer support was conducted 1-2 weeks after each nurse consultation. Peers provided psychosocial support to the patient, encouraged adherence to the recommended self-care strategies, and referred to treatment team for any complex issues. | Nurse & trained peers (gynecological cancer survivors at least 2 years post-treatment). | Individual (nurse-led sessions were conducted F2F or by telephone).   Peer support was individual via telephone. | 6 sites across 4 Australia states. Coordinated by an Australian cancer centre (Peter MacCallum Cancer Centre).   Nurse-led sessions: telehealth or at the treatment site (i.e., tour of radiation facility).   Peer support: telehealth. | 3-4 nurse-led sessions (30-60 min each). 3-4 peer support sessions. | Individually tailored based on the patient’s concerns, survivorship care plan, and barriers. | NR | Adherence: Exploratory outcomes included use of a vaginal dilator, moisturizers/creams and lubricants, as well as pelvic floor muscle exercises, assessed with a customized tool, the Adherence Questionnaire.  Fidelity: All nurse and peer sessions were audiotaped. The first 5 calls made by all peers and nurses were reviewed and feedback was provided. Then, 15% of sessions were randomly selected and assessed by a research assistant for fidelity to the protocol via nurses and peers completed checklists. Number and length of sessions were recorded. | Adherence: 71% (n=112) of intervention participants attended all available nurse-led sessions; an additional 19% (n=30) all but 1 session. 47% (n=75) of intervention participants attended all available peer sessions, and an additional 24% all-but 1 session.   The majority of women reported using a vaginal dilator (82-93%), vaginal moisturizers/creams (63-65%), and vaginal lubricants (53-67%) at all assessments.   A majority of women reported using pelvic floor muscles exercises (85-92%) at all assessments  Fidelity: 77 nurse (13% of total) and 77peer (17% of total) sessions were assessed for intervention fidelity. Fidelity was highly variable: on average, nurses delivered 77% of (range: 53–100%) and peers 83%of their intervention components (range: 58–100%), respectively. The average length of nurse and peer sessions was 35 min (range: 9–78 min) and 25 min (range: 4–71 min), respectively. |
| Du et al., 2020 | Empowerment education-based nursing interventions for sexual function | Empowerment education serves as a self-care theory that focuses on the development of health education, passing on social, medical, and pedagogical knowledge, as it emphasizes that nursing interventions based on empowerment education for patients with cervical cancer help to improve their self-care ability and perfect the work of the nursing staff. | NR | *Module* *1*: clarify problems - patients were encouraged to communicate with each other about the existing problems. Once patients determined their own problems, caregivers worked with them to analyze the root cause of these problems.  *Module 2*: expression - nursing staff were required to listen and encourage patients to talk. Focus on giving patients sufficient support and care, improving the circumstances as much as possible in order to raise the comfort level. Nurses answered questions and showed encouragement in a way that patients would have more confidence in coping with the disease.  *Module 3*: set goals - nursing staff guided patients to set goals by themselves. *Module 4*: planning - Patients participated in treatment planning with the caregivers guidance. Furthermore, they got involved in group discussion where the caregiver introduced those successful cases to other patients in a group and encouraged them to finish planning.  *Module 5*: outcome evaluation - Patients were evaluated by the nursing staff on the feasibility of the plans they made and then completed the self-evaluation under their guidance.   A health education session on cervical cancer rehabilitation, and sexual life was carried out before the operations, and at 1-6 months after the operations, for at least 30 min. | 1 doctor (responsible for the diagnosis and management of disease) + 1 psychological counsellor (for psychological assessment and counselling) + 1 charge nurse (for the implementation of nursing interventions and the evaluation of nursing efficacy) + 3 primary nurses (cooperating with the implementation of the nursing interventions and any communications with the patients) | Unclear  Likely group and F2F, though this is not clearly reported. | Unclear  China - patients attending West China Second University Hospital, Sichuan University for cervical cancer. Not explicitly stated but assuming session took place at the hospital. | NR | Individually tailored based on the patient’s concerns | NR | NR | NR |
| Robinson et al., 1999 | Psychoeducation group sessions | Regular vaginal dilation is widely recommended as a way to maintain vaginal health and good sexual functioning. However, the compliance rate with this recommendation is low. The purpose of this study was to test the effectiveness of a group psychoeducational program based on the information-motivation ± behavioral skills model of behavior change in increasing the rate of compliance. | Dilators: type and size not specified.   Educational booklet - “Sexuality and Cancer: For the Women who Has Cancer, and Her Partner”.   Handouts of content covered in group meetings. | 2 x 1.5-hour psychoeducation group sessions. Sessions were guided by the information-motivation-behavioral skills model. Information about sexuality in general and sexuality and cancer was presented using a variety of teaching aids and techniques: 3D model of the female pelvis was utilized; women were shown and able to feel different kinds of vaginal lubricants; explicit instruction for vaginal dilation was given; and the women were shown and able to handle a vibrator. The motivational component of the intervention was designed to enhance the women’s view of their sexuality and to promote the idea that sex can be pleasurable despite cancer treatment. The group format allowed for social comparisons, normalization of feelings, and social connections. The behavioral skills component focused on teaching women how to effectively use dilators and lubricants, and to perform Kegel exercises. Strategies to integrate these behaviors into daily life and into lovemaking were discussed. | Clinical psychologists | F2F, supervised group education sessions | Unclear  Assume at Tom Baker Cancer Centre though this is not explicitly reported. | 2 x 1.5-hour psychoeducation group sessions | Generic | NR | Compliance with recommendations for vaginal dilation was defined as using a vaginal dilator or having sexual intercourse or some combination of the two at a frequency of 3 times or more per week. Non-compliance was defined as a combined frequency of sexual intercourse or vaginal dilation of less than 3 times per week. The use of vaginal dilators was measured by self-report questions and frequency of sexual intercourse was measured by one question on the Sex History Form. | 44.4% of young women and 48.1% of older women were considered compliant. |
| Li et al., 2023 | Pelvic floor rehabilitation exercise + routine care | Scholars advocate that the PF rehabilitation exercise of biofeedback electrical stimulation combined with Kegel exercise should be used as a first-line treatment option for PF dysfunction diseases after cervical cancer surgery, but the safety of its clinical application and patient compliance remains controversial. | Urostym biofeedback electro-stimulator, Kegel video, patient WeChat group. | *PF biofeedback stimulation exercise* (day 3 post-surgery): patient was treated with Urosty biofedback electrical stimulator in a lateral position, giving bioelectrical stimulation at a frequency of 20Hz and a current 40-75 mA, adjusted according to the patient’s feeling of muscle throbbing without pain.  *Kegel Training*: patient lay flat, calmed breathing, contracted the gluteal muscles to lift the anus upward during inhalation, tightened the urethra, vagina and anus, held for 5—10 sec, relaxed during exhalation, repeated at 5-10 sec intervals. Patient stood with hands crossed on both shoulders, toes at 90°, heels medially as wide as the armpits, clamp firmly for 5-10 sec, relaxed on exhalation, and repeated at 5-10 sec intervals. The patient squatted down, pulled the bed rail with both hands, opened the feet shoulder-width apart, and slowly did the “squat-stand-squat” movement.  *Exercise Instruction*: Produce a video on Kegel exercise training methods, introduce the purpose and significance of Kegel exercise training to patients and their families, and demonstrate standardized movements on site before surgery.  *Establish a patient WeChat group:* send the video to the WeChat group to guide and supervise the patients to perform Kegel exercise training. | NR | Unclear  Assume individual. Home-Kegel were supervised via WeChat. | Unclear  Standardized movements were demonstrated on-site (assume hospital-based) pre-surgery. | Bioelectrical stimulation: 30 min per day for 7 days.  Kegels (pre-surgery): lying down, standing, squatting 3 movements repeated 20-30 times. 3-5 sets of 15-30 min per day pre-surgery.  Kegels (post-surgery): 1-2 sets for the first time, 2-3 sets for the 2nd and 3rd times, 4-5 sets per day from the 4th time onwards. 7-days for 1 session, 8 weeks of continuous training post- surgery. | Bioelectrical stimulation was tailored according to the patient’s feeling of muscle throbbing without pain. Kegels (post-surgery): After the patient’s vital signs were stabilized, appropriate exercise methods and frequency were selected according to the patient’s physical condition and recovery, to the extent that the patient was slightly sweating and did not feel pain and fatigue. | NR | NR | NR |
| Tung et al., 2024 | Transtheoretical model based sexual health education program | A one-session sexual health education intervention based on transtheoretical model effectively promoted sexual self-efficacy and sexual resumption in postpartum women in Taiwan. | Interactive self-help pamphlet (tutorial-in-print booklet) | Participants were provided with an interactive self-help pamphlet that had 4 main components: sexual communication and intimacy; physical sexual health adjustment (use of lubricant or vaginal moisturizer); elimination of sexual myths and gender blindness, and; reconstruction of the sexual self, which provides information on the body and sex after gynecological cancer including sex, sexual health promotion, psychological sexuality, intimate relationships, and partners. After reading the pamphlet, participants were provided with the TTM-SHE intervention at bedside, which comprised a 10-15-min F2F individualized interactive session with the nurse educator. | Nurse educator with >1 year of clinical experience in gynecological nursing and formal training in a course titled “Transtheoretical model-based sexual health education”. | F2F, individualized, interactive session.  Telephone calls from a nurse educator reminding participants to study. | Bedside.  Recruitment: gynecological cancer wards and clinics at a medical centre in norther Taiwan. | 1 x 10-15 min session | Generic and individualized (baseline assessments were used to assess the participants’ level of preparedness and determine individualized strategies for the intervention). | NR | Unclear  Strategies: women were encouraged to read the pamphlets for self-study and telephone calls from nurse educator. | NR |
| Cyr et al., 2020; 2021; 2022a; 2022b; | Multimodal pelvic floor physiotherapy | Several clinical guidelines suggest PF physical therapy among first-line treatments to address dyspareunia in cancer survivors. Nevertheless, no studies so far have evaluated the effectiveness of this conservative non-invasive and nonpharmacological intervention to reduce painful intercourse after gynecological cancer treatment. Multimodal PF physical therapy could be efficacious to treat dyspareunia as the intervention targets its biological and psychosexual pathophysiological mechanisms. Through education, manual therapy, PFM exercises using biofeedback and home exercises, physical therapy is intended to address the deleterious effects of cancer treatment on the PFM while providing support and counsel to women to reduce their pain. | Evadri intravaginal biofeedback device, graded vaginal dilators, vaginal lubricant and moisturizer, (written education materials) | The intervention combined multiple modalities including education, manual therapy, PFM exercises using biofeedback and home exercises, which included the use of a dilator.  Education topics included pathophysiology and management of dyspareunia, use of vaginal lubricant and moisturizer. Women were also guided towards resuming non-painful sexual activities. Sexual partners were invited to attend 1 session to discuss main educational topics and learn how they could assist their partner during treatment.  At each session, 20–25 min were dedicated to manual therapy techniques that were applied externally and intravaginally to the PFM. 20 min focused on PFM exercises with biofeedback using a small intravaginal probe to promote PFM relaxation and coordination, as well as strength and endurance.  Home exercises incorporated deep breathing and similar PFM exercises to those during the treatment sessions.  Participants were instructed to perform insertion techniques using a finger or graded vaginal dilators and vestibule tissue mobilization. | Experienced and certified physical therapist in women’s health.  All physical therapists received standardized training for the treatment protocol. | Individual, F2F, supervised sessions & home program. | In clinic 1 time per week, plus home exercise program.  3 x university hospitals, Canada. | 12 weekly, individual 60-min sessions.  Manual therapy technique: 20-25 min per session. PFM exercises with biofeedback: 20-min per session. A relaxation period preceded and followed the exercises that included maximal contractions (100%), podium contractions (100%/50% of maximal voluntary contractions/100%) or reversed podium contractions (50%/100%/50%), rapid contractions and 1-min sustained maximal contraction. Women performed these exercises in a lying position (weeks 1-8) followed by a sitting (weeks 9-10) and a standing position (weeks 11-12). The number of repetitions and the duration of the contraction were increased from session to session (e.g., up to 10 repetitions of 10-sec maximal contractions, 2 times). Home exercises: 5 times per week, like those performed using biofeedback under supervision. Vaginal dilators: 3 times per week (insertion exercises: introduction exercise, relaxation exercise, clock stretching exercise, oscillation exercise). | Modalities were adapted to each participant (e.g., amount of pressure applied) and progressed throughout the 12 sessions (e.g., 1-2 fingers, more pressure or stretching applied).  Modalities were intensified (e.g., more pressure applied to stretch the tissues, longer duration of the technique or exercise and greater dilator size) following each woman’s progress. | NR | Adherence rate to home exercises: ≥80% for both PFM and insertion exercises according to a diary completed by participants. Attendance rate at physical therapy sessions: ≥80% as the proportion of participants attending ≥10 sessions. | The mean adherence to home PFM and insertion exercises was respectively 93(8)% and 83(16)% for an average adherence rate of 88(10)%. The mean attendance rate at treatment sessions was 93(21)%, and 29/31(94%) women attended ≥10sessions. |
| Brennen et al., 2023 | Telehealth-delivered PFM training | To date, no studies have investigated telehealth-delivered PFM training to urinary incontinence after gynecological cancer treatment without a clinical assessment. One study investigated the feasibility of telehealth-delivered PFM training to treat stress urinary incontinence in women with breast cancer and found that it may be feasible and potentially beneficial., However, we do not know if similar results will be observed in women with gynecological cancer due to differences in demographics, and clinical and treatment characteristics between breast and gynecological cancer survivors. | Femfit (Junofem) intravaginal pressure biofeedback device, mobile phone application | In the first telehealth session, participants were guided through how to contract their PFM, use the femfit® and complete their home exercise program. Participants received 7 supervised, individual PF training T telehealth sessions, during which they used the intra-vaginal sensor. They followed a pre-defined daily home exercise program installed on the femfit® mobile device application which was based on a published PFM training program. Education-based therapies were also provided alongside PFM training depending on the participant’s symptoms. These included PFM pre-contraction for increases in intra-abdominal pressure, fluid management, urgency suppression techniques, urge trigger desensitization strategies, voiding and defecation dynamics, toilet posture and dietary fibre intake. | Registered physiotherapist with postgraduate qualifications and 16 years clinical experience. | Individual supervised telehealth sessions using Zoom on their mobile device (no F2F assessments or sessions). | Participants’ home | 12 weeks, fortnightly sessions, each session (total - 7) was 30-60 min.   Daily PFM training home program: 3 sets of 6-10 maximal contractions, 6-10 fast contractions, 3 endurance contractions and 3 contractions with cough (knack) during the intervention phase. | Generic start level. Every 4 weeks, the number of repetitions and duration of each contraction increased, and positions were progressed from across-gravity (lying) to against gravity (sit, then stand). The program was tailored to each individual by delaying the progression timings if the participant was not yet able to achieve the scheduled progression. | NR | Strategies: Reminder, biofeedback, discussion and problem-solving barriers, action planning.  Engagement measured by the attendance rate: calculated at the end of the intervention period using the number of telehealth sessions attended out of 7.  Adherence to the home exercise program during the intervention period: calculated at the end of the intervention period using the average number of days per week that the participant completed the home exercise program, as recorded in the femfit® exercise diary. Completion of exercise sessions was automatically recorded in the femfit ® application. Exercise sets could also be added manually by the participant if they completed the exercise without using the femfit® application. Adherence to ongoing PFM training during the follow-up period was assessed by self-report questions on how many times per week the participant had completed PFM training.  Fidelity of treatment receipt for the home exercise program: assessed in each telehealth session by participants’ ability to verbally describe the home program components and a screenshot of their femfit® exercise diary, documented in session notes by the researcher. | Of the 32 participants who received the intervention, 30 (94%) attended at least 6 telehealth sessions. The mean percentage of daily PFM training sessions completed was 79% (SD ± 21%). 24 participants (75%) completed 5-7 PFM training sessions per week, and only 3 participants (9.4%) completed fewer than 3 PFM training sessions per week. At the 3-month follow-up time point, 25 (78%) participants reported they were doing regular PFM training, with 16 reporting that they performed PFM training at least 3 times a week. |
| Bernard et al., 2021 | In-home rehabilitation program | There is a high prevalence of urinary incontinence among endometrial cancer survivors. They are also known to present with PFM alterations. Evidence on the effects of conservative interventions for the management of urinary incontinence is scarce. This study aims at verifying the effects of an in-home rehabilitation program, including the use of a mobile technology, to reduce urinary incontinence severity in endometrial cancer survivors. | Elvie trainer (intravaginal sensor) + iPad mini + mobile app + education clips | The program included 3 main components - a PMF training program, bladder training regime, and counselling on lifestyle habits. The PFM training was delivered in conjunction with the Elvie Trainer and its mobile app. Participants received their daily exercise program through the app. Weekly telephone follow-ups were carried out by a physiotherapist to offer personalized advice on exercise variety and positions for training. For the last two components, motivational counselling was given during the weekly follow-ups to teach urge suppression techniques, bladder emptying techniques, mindfulness exercises and lifestyle choices that may influence bladder symptoms. Short educational videoclips were also available on the iPad for consultation when participants had a question. | Physiotherapist | Individual, telephone + mobile app support, in-home | In-home program.   Assessment completed in the clinical laboratory, Canada. | 12 weeks, daily exercise program through mobile app + weekly telephone calls | Appears generic, but with ‘Personalised advice on exercise variety and positions for training’. | NR | To track adherence, the Elvie app records each session performed with the Trainer, documenting progression and training sessions, and these data were extracted at the end of the study. In addition, the participants were invited to write down the exercises performed in an exercise log, which was also collected after the study. Technical issues encountered were also documented. Adherence to the program was promoted by the therapist using various adherence promotion strategies, which were documented at every telephone session. | The total number of training sessions recorded by Elvie app ranged between 24-124 sessions per participant (mean number of sessions per week: 5.6), with 5 participants concurrently reporting these sessions on their exercise log.   Throughout all telephone follow-ups, the 3 most frequent adherence promotion strategies used by the therapist were (1) providing information on benefits and consequences, (2) providing general encouragement and (3) providing feedback on performance. |
| Sacomori et al., 2020 | Non-supervised home-based PFM exercises | Techniques such as vaginal desensitization and PF re-education through exercises, vaginal cones and electrostimulation have been found to play a role in preventing the onset of complications. However, there is a lack of strong evidence to support the recommendation of these therapies as prehabilitation. | Audio recording and educational flyer | The intervention included 1 x 30-min physical therapy session to teach PFM exercises. Patients were instructed to practice at least 2 times per day from home. An educational flyer and an audio recording with the instructions for the exercises were given to the patients. Information on PF anatomy and function and the importance of PFM exercises in preventing dysfunctions was also provided. | Physical therapist specialist in oncology and PF rehab | Individual, F2F (physical therapy session) then individual at home. | Rehabilitation service of the National Cancer Institute Chile | A single 30-min physical therapy session plus exercises 2 times per day at home with a 1 month follow-up.  Home exercise recommendations: low contractions (8 x 6-sec maximal voluntary contractions with a rest of 10 sec), 1-sec contractions (8 maximal voluntary contractions followed by relaxation), and the “knack” (a voluntary precontraction of the PF before activities that increased intra-abdominal pressure). | Unclear | NR | Adherence to non-supervised home-based PFM exercises was assessed through an exercise diary in which women registered their completion of the recommended exercises.  Steps were taken to promote home-based PFM exercise adherence. An educational flyer and an audio recording with the instructions for the exercises were given to the patients. Information on PF anatomy and function and the importance of PFMEs in preventing dysfunctions was also provided. | Among the women who returned for re-evaluation, adherence to home-based PFM exercises was high. All reported doing the exercises; however, 6 women (21.4%) did not return their diaries. With the exclusion of the women who did not return their diaries, the average number of days per week for performing the exercises was 4.9 (SD=1.5). |
| Bober et al., 2018; Hungr et al., 2020 | Sexual therapy and rehabilitation after treatment for ovarian cancer (psychoeducational intervention) | Brief, group-based treatment that integrates targeted sexual health education with PF awareness and relaxation training as well as elements of mindfulness-based cognitive therapy to improve sexual function and decrease distress in women with treatment-related sexual dysfunction. | Slides and take-home educational materials (e.g., instructions for a mindfulness-based body scan and muscle-relaxation exercises and information about personal products such as vaginal moisturizers and dilators as well as resources for sexual health Web sites and books) | A single half-day (3.5 hours) psychoeducational group sessions take-home educational materials, and a single booster telephone call. The groups session was structured around 4 primary modules: sexual health information, education about treatment-related sexual problems, communication strategies; relaxation training and body awareness (PF and strategies for PF relaxation); mindfulness-based cognitive therapy (interplay of thoughts, feelings, and behavior), and; individual-based activity planning. | Group session: Clinical psychologist who is an expert in sexual rehabilitation after cancer treatment.  Booster call: training intervention assistant who attended the intervention workshop. | Group, F2F and written instructions were given | NR  Recruitment: Dana-Farber Cancer Institute. | A single 3.5-hour session with 15-min booster telephone call after 1 month. | Generic and individually tailored for the individual-based activity planning (identification of problems and personal goals and plan with 2 actionable steps). | NR | Strategies: Approximately 1 month after the group session, each woman received a single brief telephone booster intended to support the completion of her personal action plan. | NR |
| Brotto et al., 2012 | Psychoeducational intervention | Evidence suggests that mindfulness-based strategies may be especially suitable for addressing sexual difficulties in women. | NR | Women received 3 x 90-min individual sessions, including education on causes of sexual difficulties, cognitive challenging of maladaptive sexual beliefs, prevalence of sexual dysfunction after cancer, body image and mindfulness exercises, arousal-enhancing techniques, and PF health. | Registered psychologist/sex therapist | Individual | NR | 3 x 90-min sessions (one per month) | NR (likely generic) | NR | NR | NR |
| Brotto et al., 2008 | Brief mindfulness-based cognitive behavioral intervention | Psychoeducation, which combines education and information with elements of psychological therapy, has been found to significantly improve frequency of coital activity, and enhances compliance with sexual rehabilitation, reduces fear about intercourse, and improves sexual knowledge. | Therapist manual and participant handouts (booklet of information and exercises) | 3 x 60-min audio recorded segments including factors of sexuality, cognitive challenging of maladaptive sexual beliefs, prevalence rates of sexual difficulty following cancer and its treatment, connection between sexual relationship and sexuality, and body image and sexuality, techniques to augment sexual arousal, loosening exercises designed to strengthen the larger muscles of the body; using self-sensate focus to tune in to sexual arousal; potential role of erotica, fantasy, and vibrators in augmenting natural sexual arousal response. As well as 5-7 hours of personal time per month. | Unclear  Investigator | Individual, audio-recorded | NR | 3 x 60-min audio-recorded segments, 1 segment per month, 3 -month period, + 5-7 hours of personal time per month | NR (likely generic) | NR | Participants were encouraged to spend 5-7 hours the next month working through the material.   A self-rating (0–100%) for homework completion at each session. | Homework completion at each session - mean for sessions 1, 2, and 3 were 90%, 82%, and 82% |

F2F: face-to-face, NR: not reported, PF: pelvic floor, PFM: pelvic floor muscle, post-op: post-operative
