# Supplementary material for: Effectiveness of Pelvic Floor Muscle and Education-Based Therapies on Bladder, Bowel, Vaginal, Sexual, Psychological Function, Quality of Life, and Pelvic Floor Muscle Function in Females Treated for Gynecological Cancer: A Systematic Review
Source: Curr Oncol Rep. 2024 Aug 23;26(11):1293–320. doi: 10.1007/s11912-024-01586-7 (PMC11579103; doi:10.1007/s11912-024-01586-7)
Supplement: Supplementary file 3 — Supplementary file3 (DOCX 222 KB) [file 11912_2024_1586_MOESM3_ESM.docx]

**Supplementary Information 3. Effectiveness of Conservative Therapies in Gynecological Cancer Populations.**

(a) Bladder Function

| **Bladder function** | **Intervention arms** | | **Outcomes** | | **Results** | | **Considerations for interpretation** | | |
| --- | --- | --- | --- | --- | --- | --- | --- | --- | --- |
| **Study; sample size; type of cancer n (%)** | **Treatment group (TG)** | **Comparator group (CG)** | **What was measured?** | **Outcome measure/tool used** | **Values, significance; or narrative description** | **Direction of findings** | **Sample size calculation, primary outcome, end timepoint specified (if >1 follow-up timepoints); baseline outcome measure variance; floor/ceiling effect bias; adjustment for between-group differences on key characteristics and baseline outcomes** | **Adequate statistical analysis (ITT, per protocol, both, or unclear; multiple comparisons without adjustment)** | **TIDieR score (range 0-12); quality of intervention (dose response issues); interpretation of intervention effect (in relation to timing of delivery and of outcome assessment)** |
| **6 RCTs, between-group comparisons** | | | | | | | | | |
| Sun 2023 [18];  n=130;  Cervical (100%) | PFM therapy (active) + education | Routine nursing care | Urogenital distress;  Urinary incontinence impact | UDI-6 (from PFDI-20);  UIQ-7 (from PFIQ-7) | Post-intervention mean±SD:  UDI-6: TG 40±3 vs CG 47±5, p<0.001*;  UIQ-7: TG 40±2 vs CG 53±6, p<0.001* | Difference in favor of TG | No sample size calculation provided;  Primary outcomes specified: PF distress (PFDI-20) and PF impact (PFIQ-7) | ITT;  Multiple comparisons without adjustment | 7;  Unclear duration of intervention; Short-term follow-up; Time after cancer treatment NR |
| Zong 2022 [19];  n=166;  Cervical (100%) | PFM therapy (active) + clean intermittent self-catheterization | Clean intermittent self-catheterization (+ education on urinary retention and urinary incontinence) | PVR | PVR volume | Post-intervention mean±SD:  TG 46.79±10.20 vs CG 67.37±18.47, p<0.001* | Difference in favor of TG | Sample size formula provided (no detail on which outcome and follow-up timepoint were selected as primary) | ITT;  Multiple comparisons without adjustment | 7;  Short-term follow-up; Time since cancer diagnosis NR;  Time since cancer treatment: 3 days pre-surgery, 3 and 10 days post-surgery |
| Li 2019 [20];  n=91;  Cervical (100%) | PFM therapy (electrostimulation) + intermittent catheterization + bladder function training | Intermittent catheterization + bladder function training | PVR | PVR volume | Post-intervention mean±SD:  TG 56.85±29.44 vs CG 95.79±24.07, p=0.000* | Difference in favor of TG | No sample size calculation provided; No primary outcome specified; No baseline values (uncertainty of effect of intervention) | ITT;  Multiple comparisons without adjustment | 6;  Short-term follow-up; Time since cancer treatment: 11 days |
| Rutledge 2014 [22];  n=40;  Uterine (60%)  Ovarian (23%)  Cervical (13%) | PFM therapy (active) + education | Usual care (no intervention) | Urinary incontinence severity; type; distress; impact | ISI; QUID; UDI-6 (from PFDI-20);  IIQ-SF | ISI score presented pictorially as n of participants with either mild or moderate/severe urinary incontinence. From picture and text TG: baseline 7/20 mild, 13/20 moderate/severe, post-intervention 12/20 mild, 8/20 moderate/severe. From picture and text: CG: baseline 4/20 mild, 16/20 moderate/severe, post-intervention 6/20 mild, 14/20 moderate/severe, p=NR.  QUID: baseline data only.  UDI-6: at 3 months TG 70% vs CG 50% reported lack of bother from their urinary incontinence, p=0.62.  IIQ-SF: “not different between the two groups as compared pre and post intervention.” | ISI results appear not to be statistically significant between groups, and no difference between groups for UDI-6 and IIQ-SF.  No differences between groups | Sample size based on improvement in Patient Global Impression of Improvement (primary outcome); Unclear risk of baseline outcome measure variance and floor/ceiling effect bias (no data provided) | ITT | 11;  Potentially adequate dosage but only 1 supervised session and adherence not reported; Short-term follow-up; Time since cancer treatment: median 2.5 (range 1-5) in years |
| Yang 2012 [23];  n=28;  Cervical (93%)  Endometrial (7%) | PFM therapy (active) + education + core-strengthening program + hip muscle stretching exercises | Leaflet with home-based PFM exercise, lifestyle advice and a telephone number for further explanations | Bladder symptoms | Bladder function questions from APFQ | Mean(SD) of change from baseline:  APFQ bladder function scores: TG -0.83(1.08) vs CG -0.83(1.25), p=0.710 | No differences between groups | Small sample size; No sample size calculation provided;  Primary outcomes listed as PF function (PFM strength and motor evoked potentials of sacral nerve) and quality of life; Potential risk of baseline outcome measure variance and floor/ceiling effect bias | Appears to be ITT but selective exclusion of participants experiencing aggravation of PF symptoms and/or had some difficulty in continuing the exercises;  Multiple comparisons without adjustment | 9;  Short duration; Short-term follow-up; Time since cancer treatment: median 1.2 (range 1-5) in years |
| Cerentini 2019 [24];  n=88;  Cervical (100%) | PFM therapy (passive) | Standard guidance from nursing team care (including re advice to use dilators) | - | - | - | Not interpretable | Sample size based on percentage of change in vaginal dimensions (primary outcome) in women with vaginal stenosis​ (sample size achieved for recruitment but not for follow-up measures, high attrition) | Both;  Multiple comparisons with Bonferroni post hoc tests | 8;  Short-term follow-up; Time since cancer diagnosis/treatment NR |
| **1 non-RCT with 2 groups, between-group comparisons** | | | | | | | | | |
| Li 2023 [29];  n=120;  Cervical (100%) | PFM therapy (active + electrostimulation) + routine care | Routine care | PVR; Bladder compliance; Bladder detrusor systolic pressure; Urethral closure pressure | Urodynamics (PVR volume, etc.); Bladder function recovery rate and urinary retention incidence: Residual urine volume <50 ml with good recovery of bladder function was defined as grade I; residual urine volume 50-100 ml with slightly poor recovery of bladder function was defined as grade II; residual urine volume >100 ml with poor recovery of bladder function was defined as grade III; and extremely difficult urination with no recovery of bladder function was defined as grade IV. | Results presented in a figure (baseline and 2 weeks post-intervention) – estimates taken from graphics:  PVR <50 ml: TG 70-75% of participants vs CG 50-55% of participants, p<0.05*, and no difference for Grade II, III, VI;  Urinary retention: TG 0-10% of participants vs CG 15-20% of participants, p<0.05*;  Bladder compliance (ml/cmH_2_O): TG 80-90 vs CG 55-60, p<0.05*;  Bladder detrusor systolic pressure (cmH_2_O): TG 35-40 vs CG 20-30, p<0.05*;  Urethral closure pressure (cmH_2_O): TG 90-92 vs CG 88-90, p=not significant. | Difference in favor of TG, except for urethral closure pressure (no differences between groups) | No sample size calculation provided; No primary outcome specified; Unclear risk of baseline outcome measure variance and floor/ceiling effect bias (no data provided) | ITT;  Multiple comparisons without adjustment | 6;  Potentially adequate dosage; Short- and medium-term follow-up; Time since cancer diagnosis/treatment NR |
| **4 non-RCTs with single group, within-group comparisons** | | | | | | | | | |
| Cyr 2020, 2021, 2022a, 2022b [10, 31-33];  n=31;  Endometrial (64.5%)  Cervical (35.5%) | PFM therapy (active + passive) + education | NA | Urinary symptoms | ICIQ-SF UI | Median change (Q1 to Q3) from baseline to 2 weeks post-intervention:  ICIQ-UI SF: 0 (-3.0 to 0), p=0.001*;  Mean(95%CI) change from baseline to 12 months follow-up:  ICIQ-UI SF: -2.0 (-3.3 to -0.6), p=0.002* | Improvement in outcome | Sample size calculation provided, but not for this outcome – based on feasibility outcomes and sample size achieved | ITT;  Multiple comparisons, no adjustment for pre-post data but Bonferroni correction for follow-up data | 11;  Short- and long-term follow-up; Time since cancer treatment: median 38 (Q1 9; Q3 70) in months |
| Brennen 2023 [34];  n=36;  Endometrial/uterine (69%) Cervical (19%) Ovarian (11%) | PFM therapy (active) + education | NA | Urinary incontinence symptom prevalence; severity; impact | %; ICIQ-UI SF | Mean(95%CI) change:  Urinary incontinence symptom prevalence: -3 (-13 to 6), p=NR;  ICIQ-UI SF: -1.65 (-3.00 to -0.15), p=NR | Improvement in all outcomes, but confidence intervals cross the line of no effect for urinary incontinence symptom prevalence | Sample size calculation provided, but not for this outcome – based on feasibility outcomes and sample size achieved; Unclear risk of baseline outcome measure variance and floor/ceiling effect bias | ITT;  Multiple comparisons without adjustment | 11;  Potentially adequate dosage; Short- and medium-term follow-up; Time since cancer treatment: median 17.5 (IQR 27) in months |
| Bernard 2021 [35];  n=8;  Endometrial (100%) | PFM therapy (active) + education | NA | Urinary signs; symptoms | ICIQ-UI SF; Pad test; 3-day diary | Mean:  Pad test: baseline 9.4 g and post-intervention 0.4 g, p<0.0001*;  Bladder diary (n leaks): baseline 3.8 and post-intervention 0.6, p<0.0001*;  Bladder diary (n urges): baseline 7.4 and post-intervention 1.1, p<0.0001*  Bladder diary (n voids) baseline 23.5 and post-intervention 19.7, p=0.04*  ICIQ-UI SF baseline 9.3 and post-intervention 6.3, p=0.0003* | Improvement in all outcomes | No sample size calculation provided;  Primary outcome specified: urinary incontinence (pad test); Unclear risk of baseline outcome measure variance and floor/ceiling effect bias | ITT;  Multiple comparisons without adjustment | 11;  Short-term follow-up; Time since cancer treatment: mean 43 (range 19-55) in months |
| Sacomori 2020 [36];  n=49;  Cervical (100%) | PFM therapy (active) | NA | Urinary incontinence prevalence; Impact of urinary incontinence | %; ICIQ-SF UI | Urinary incontinence prevalence: baseline 15/28 (54%) and post-intervention 13/28 (46%), p=NR;  Median(IQR):  ICIQ-SF UI: baseline 4(9.5) and post-intervention 3(9), p=0.794 | No improvement in outcomes | No sample size calculation provided;  Primary outcome specified: PFM strength (vaginal bi-digital evaluation using MOS); Unclear risk of baseline outcome measure variance and floor/ceiling effect bias | Per protocol (n=28) due to high attrition; Multiple comparisons without adjustment | 10;  Unclear duration of intervention; Unclear follow-up timepoint; Time since cancer diagnosis NR;  Time since cancer treatment up to 1 month post-radiotherapy |
| APFQ: Australian Pelvic Floor Questionnaire, BFLUTS: Bristol Female Lower Urinary Tract Symptoms Questionnaire, CG: comparator group, CI: confidence interval, IIQ-SF: Incontinence Impact Questionnaire – Short Form, ICIQ-UI SF: International Consultation on Incontinence Questionnaire-Urinary Incontinence Short Form, ISI: Incontinence Severity Index, ITT: intention-to-treat, MOS: modified Oxford scale, PFDI-20: Pelvic Floor Distress Inventory-20, NA: not applicable, NR: not reported, Pelvic Floor Distress Inventory-20, PFIQ-7: Pelvic Floor Impact Questionnaire-7, PF: pelvic floor, PFM: pelvic floor muscle, PVR: post-void residual, QUID: Questionnaire for Urinary Incontinence Diagnosis, RCT: randomized controlled trial, SD: standard deviation, TG: treatment group, UIQ-7: Urinary Impact Questionnaire-7, UDI-6: Urinary Distress Inventory-6  *Difference reached statistical significance (p<0.05) or reached statistical significance according to methods used by the authors. | | | | | | | | | |

(b) Bowel Function

| **Bowel function** | **Intervention arms** | | **Outcomes** | | **Results** | | **Considerations for interpretation** | | |
| --- | --- | --- | --- | --- | --- | --- | --- | --- | --- |
| **Study; sample size; type of cancer n (%)** | **Treatment group (TG)** | **Comparator group (CG)** | **What was measured?** | **Outcome measure/tool used** | **Values, significance; or narrative description** | **Direction of findings** | **Sample size calculation, primary outcome, end timepoint specified (if >1 follow-up timepoints); baseline outcome measure variance; floor/ceiling effect bias; adjustment for between-group differences on key characteristics and baseline outcomes** | **Adequate statistical analysis (ITT, per protocol, both, or unclear; multiple comparisons without adjustment)** | **TIDieR score (range 0-12); quality of intervention (dose response issues); interpretation of intervention effect (in relation to timing of delivery and of outcome assessment)** |
| **3 RCTs, between-group comparisons** | | | | | | | | | |
| Sun 2023 [18];  n=130;  Cervical (100%) | PFM therapy (active) + education | Routine nursing care | Colorectal-anal distress;  Colorectal-anal impact | CRADI-8 (from PFDI-20);  CRAIQ-7 (from PFIQ-7) | Post-intervention mean±SD:  CRADI-8: TG 31±4 vs CG 39±3, p<0.001*;  CRAIQ-7: TG 41±3 vs CG 48±4, p<0.001* | Difference in favor of TG | No sample size calculation provided;  Primary outcomes specified: PF distress (PFDI-20) and PF impact (PFIQ-7) | ITT;  Multiple comparisons without adjustment | 7;  Unclear duration of intervention; Short-term follow-up; Time after cancer treatment NR |
| Yang 2012 [23];  n=28;  Cervical (93%)  Endometrial (7%) | PFM therapy (active) + education + core-strengthening program + hip muscle stretching exercises | Leaflet with home-based PFM exercise, lifestyle advice and a telephone number for further explanations | Bowel symptoms | Bowel function questions from APFQ | Mean(SD) of change from baseline:  APFQ bowel function scores: TG -0.67(0.65) vs CG -0.51(1.47), p=0.412 | No difference between groups | Small sample size; No sample size calculation provided;  Primary outcomes listed as PF function (PFM strength and motor evoked potentials of sacral nerve) and quality of life; Potential risk of baseline outcome measure variance and floor/ceiling effect bias | Appears to be ITT but selective exclusion of participants experiencing aggravation of PF symptoms and/or had some difficulty in continuing the exercises;  Multiple comparisons without adjustment | 9;  Short duration; Short-term follow-up; Time since cancer treatment: median 1.2 (range 1-5) in years |
| Cerentini 2019 [24];  n=88;  Cervical (100%) | PFM therapy (passive) | Standard guidance from nursing team care (including re advice to use dilators) | - | - | - | Not interpretable | Sample size based on percentage of change in vaginal dimensions (primary outcome) in women with vaginal stenosis​ (sample size achieved for recruitment but not for follow-up measures, high attrition) | Both;  Multiple comparisons with Bonferroni post hoc tests | 8;  Short-term follow-up; Time since cancer diagnosis/treatment NR |
| **0 non-RCT with 2 groups, between-group comparisons** | | | | | | | | | |
| **3 non-RCTs with single group, within-group comparisons** | | | | | | | | | |
| Cyr 2020, 2021, 2022a, 2022b [10, 31-33];  n=31;  Endometrial (64.5%)  Cervical (35.5%) | PFM therapy (active + passive) + education | NA | Bowel pattern; control; impact on quality of life | ICIQ-B | Median change (Q1 to Q3) from baseline to 2 weeks post-intervention:  ICIQ-B pattern: -1.0 (-1.53 to 0), p=0.005*;  ICIQ-B control: -1.0 (-3.0 to 0), p=0.010*;  ICIQ-B quality of life: -1.0 (-4.0 to 0.5), p=0.018* | Improvement in all outcomes | Sample size calculation provided, but not for this outcome – based on feasibility outcomes and sample size achieved | ITT;  Multiple comparisons, no adjustment for pre-post data but Bonferroni correction for follow-up data | 11;  Short- and long-term follow-up; Time since cancer treatment: median 38 (Q1 9; Q3 70) in months |
| Brennen 2023 [34];  n=36;  Endometrial/uterine (69%) Cervical (19%) Ovarian (11%) | PFM therapy (active) + education | NA | Anorectal symptom prevalence; Anorectal pattern; control; impact on quality of life | %; ICIQ-B | Mean(95%CI) change:  Anorectal symptom prevalence (fecal incontinence): -19 (-35 to -4), p=NR;  Mean(95%CI) change:  ICIQ-B pattern: -0.58 (-1.08 to -0.08), p=NR;  ICIQ-B control: -0.62 (-1.89 to 0.62), p=NR;  ICIQ-B impact on quality of life: -1.46 (-2.85 to -0.08), p=NR | Improvement in all outcomes, but confidence intervals cross the line of no effect for ICIQ-B pattern | Sample size calculation provided, but not for this outcome – based on feasibility outcomes and sample size achieved; Unclear risk of baseline outcome measure variance and floor/ceiling effect bias | ITT;  Multiple comparisons without adjustment | 11;  Potentially adequate dosage; Short- and medium-term follow-up; Time since cancer treatment: median 17.5 (IQR 27) in months |
| Sacomori 2020 [36];  n=49;  Cervical (100%) | PFM therapy (active) | NA | Fecal incontinence prevalence; gas incontinence prevalence | % | Fecal incontinence prevalence:  baseline 11/28 (39%) and post-intervention 7/28 (25%), p=NR;  Gas incontinence prevalence: baseline 21/28 (75%) and post-intervention 8/28 (29%), p=NR | Mixed;  Improvement in gas incontinence prevalence:  No improvement in fecal incontinence prevalence | No sample size calculation provided;  Primary outcome specified: PFM strength (vaginal bi-digital evaluation using MOS); Unclear risk of baseline outcome measure variance and floor/ceiling effect bias | Per protocol (n=28) due to high attrition; Multiple comparisons without adjustment | 10;  Unclear duration of intervention; Unclear follow-up timepoint; Time since cancer diagnosis NR;  Time since cancer treatment up to 1 month post-radiotherapy |
| APFQ: Australian Pelvic Floor Questionnaire, CI: confidence interval, CG: comparator group, CRAIQ-7: Colorectal-Anal Impact Queselfstionnaire-7, CRADI-8: Colorectal-Anal Distress Inventory-8, ICIQ-B: International Consultation on Incontinence Questionnaire-Bowel, ITT: intention-to-treat, MOS: modified Oxford scale, NA: not applicable, NR: not reported, PFDI-20: Pelvic Floor Distress Inventory-20, PFIQ-7: Pelvic Floor Impact Questionnaire-7, PF: pelvic floor, PFM: pelvic floor muscle, RCT: randomized controlled trial, SD: standard deviation, TG: treatment group  *Difference reached statistical significance (p<0.05) or reached statistical significance according to methods used by the authors. | | | | | | | | | |

(c) Vaginal Function

| **Vaginal function** | **Intervention arms** | | **Outcomes** | | **Results** | | **Considerations for interpretation** | | |
| --- | --- | --- | --- | --- | --- | --- | --- | --- | --- |
| **Study; sample size; type of cancer n (%)** | **Treatment group (TG)** | **Comparator group (CG)** | **What was measured?** | **Outcome measure/tool used** | **Values, significance; or narrative description** | **Direction of findings** | **Sample size calculation, primary outcome, end timepoint specified (if >1 follow-up timepoints); baseline outcome measure variance; floor/ceiling effect bias; adjustment for between-group differences on key characteristics and baseline outcomes** | **Adequate statistical analysis (ITT, per protocol, both, or unclear; multiple comparisons without adjustment)** | **TIDieR score (range 0-12); quality of intervention (dose response issues); interpretation of intervention effect (in relation to timing of delivery and of outcome assessment)** |
| **3 RCTs, between-group comparisons** | | | | | | | | | |
| Sun 2023 [18];  n=130;  Cervical (100%) | PFM therapy (active) + education | Routine nursing care | POP symptoms; impact | POPDI-6 (from PFDI-20);  POPIQ-7 (from PFIQ-7) | Post-intervention mean±SD:  POPDI-8: TG 40±3 vs CG 50±7, p<0.001*;  POPIQ-7: TG 40±3 vs CG 46±6, p<0.001* | Difference in favor of TG | No sample size calculation provided;  Primary outcomes specified: PF distress (PFDI-20) and PF impact (PFIQ-7) | ITT;  Multiple comparisons without adjustment | 7;  Unclear duration of intervention; Short-term follow-up; Time after cancer treatment NR |
| Cerentini 2019 [24];  n=88;  Cervical (100%) | PFM therapy (passive) | Standard guidance from nursing team care (including re advice to use dilators) | Vaginal dimensions: length; width; area | Hysterometer; Vaginal dilators; Vaginal speculum | Post-intervention mean±SD:  Length: TG 6.9±0.3 vs 7.2±0.3 cm, p=0.111;  Width: TG 8.5±0.8 vs CG 7.3±0.7 cm, p=0.484;  Area: TG 41±6.9 vs CG 41±6.8 cm^2^, p=0.743 | No differences between groups | Sample size based on percentage of change in vaginal dimensions (primary outcome) in women with vaginal stenosis​ (sample size achieved for recruitment but not for follow-up measures, high attrition) | Both;  Multiple comparisons with Bonferroni post hoc tests | 8;  Potentially adequate dosage but adherence to protocol declined and high attrition in both groups; Short-term follow-up; Time since cancer diagnosis/treatment NR |
| Schofield 2020 [26];  n=318;  Endometrial/uterine (52%)  Cervical (41%)  Vulval (8%)  Vaginal (4%)  Ovarian (5%)  Fallopian tube (2%) | Education (psycho-education nurse- and peer-led psycho-educational intervention) | Usual care | Vaginal stenosis | LENT SOMA Scale | Results for end timepoints 1 and 2 NR; Compared with ratings at baseline, higher (worse) stenosis ratings were not significantly more likely at follow-up timepoint 4 and follow-up timepoint 5 in the CG and intervention arm differences were negligeable, all p>0.05. | Unclear (likely no differences between groups) | Sample size based on psychological distress (HADS at end timepoints 1 and 2) (primary outcome); Significant difference in vaginal stenosis prevalence at baseline between groups (TG 90% vs CG 78%) | ITT;  Multiple comparisons without adjustment | 11;  Unclear (low) dosage of therapy and post-study audit found variation regarding vaginal dilator management; 2 end timepoints with no data provided for this outcome; Time since cancer diagnosis/treatment NR |
| **0 non-RCT with 2 groups, between-group comparisons** | | | | | | | | | |
| **3 non-RCTs with single group, within-group comparisons** | | | | | | | | | |
| Cyr 2020, 2021, 2022a, 2022b [10, 31-33];  n=31;  Endometrial (64.5%)  Cervical (35.5%) | PFM therapy (active + passive) + education | NA | Vaginal atrophy signs; Vaginal length; Vaginal symptoms; Impact on quality of life | VAI; POP-Q; ICIQ-VS | Median (Q1 to Q3) at baseline and 2 weeks post-intervention:  VAI: fewer women presented significant signs of vaginal atrophy after treatment according to the total score, p=0.002*. Significant changes were observed in the subscales evaluating skin elasticity and turgor, vaginal mucosa thickness and rugosity as well as vaginal depth, p≤0.029*;  POP-Q: baseline 7.5 cm (6.5 to 8.5) and 2 weeks post-intervention 8.5 cm (7.0 to 9.0), p<0.001*;  Median change (Q1 to Q3) from baseline to 2 weeks post-intervention:  ICIQ-VS vaginal symptoms:  -5.0 (-9.0 to -2.0), p<0.001*;  ICIQ-VS impact on quality of life: -1.5 (-5.3 to 0) p<0.001*;  Mean(95%CI) change from baseline to 12 months follow-up:  ICIQ-VS vaginal symptoms: -6.3 (-8.6 to -4.0), p<0.001* | Improvement in all outcomes | Sample size calculation provided, but not for this outcome – based on feasibility outcomes and sample size achieved | ITT;  Multiple comparisons, no adjustment for pre-post data but Bonferroni correction for follow-up data | 11;  Short- and long-term follow-up; Time since cancer treatment: median 38 (Q1 9; Q3 70) in months |
| Brotto 2012 [39];  n=31;  Endometrial (65%)  Cervical (26%) Both (10%) | Education (psychoeducational intervention) | NA | Physiological sexual arousal | VPA (% change from neutral to erotic stimuli) | Mean(SD):  % VPA change from neutral to erotic stimuli: baseline 77% increase and post-intervention 86% increase, p>0.05 | No differences between groups | No sample size calculation provided; Primary outcome specified: sexual function (FSFI); Unclear risk of baseline outcome measure variance and floor/ceiling effect bias | Unclear; Multiple comparisons without adjustment, except self-report items where p=0.0045 (0.05/11 analyses) | 6;  Potentially low dosage and mismatch between intervention (education) and outcome (physiological); Short-term follow-up; Time since cancer treatment NR |
| Brotto 2008 [40];  n=22;  Cervical (59%) Endometrial (41%) | Education (brief mindfulness-based cognitive behavioral intervention) | NA | Physiological sexual arousal | VPA (% change from neutral to erotic stimuli) | Mean(SD):  % VPA change from neutral to erotic stimuli (according to figure): baseline 40(8)% increase and post-intervention 56(8)% increase, p>0.05 | No differences between groups | No sample size calculation provided; Primary outcome specified: sexual arousal (DASA from session 1 to session 4); Unclear risk of baseline outcome measure variance and floor/ceiling effect bias | Unclear; Multiple comparisons without adjustment, except Bonferroni correction to Film Scale where p=0.008 (0.05/6 analyses) | 8;  Potentially low dosage and mismatch between intervention (education) and outcome (physiological); Short-term follow-up; Time since cancer treatment: mean 54 (range 6-115) in months |
| CG: comparator group, CI: confidence interval, DASA: Detailed Assessment of Sexual Arousal, FSFI: Female Sexual Function Index, ICIQ-VS: International Consultation on Incontinence Questionnaire - Vaginal Symptoms, ITT: intention-to-treat, LENT SOMA: Late Effects of Normal Tissues/Subjective-Objective Management Analytic, NA: not applicable, NR: not reported, PFDI-20: Pelvic Floor Distress Inventory-20, PFIQ-7: Pelvic Floor Impact Questionnaire-7, PFM: pelvic floor muscle, POP: pelvic organ prolapse, POPDI-6: Pelvic Organ Prolapse Distress Inventory-6, POP-Q: Pelvic Organ Prolapse – Quantification, POPIQ-7: Pelvic Organ Prolapse Impact Questionnaire-7, RCT: randomized controlled trial, SD: standard deviation, TG: treatment group, VAI: Vaginal Atrophy Index, VAS: Vaginal Assessment Scale, VPA: vaginal pulse amplitude, VuAS: Vulvar Assessment Scale  *Difference reached statistical significance (p<0.05) or reached statistical significance according to methods used by the authors. | | | | | | | | | |

(d) Overall Pelvic Floor Function

| **Overall pelvic floor function** | **Intervention arms** | | **Outcomes** | | **Results** | | **Considerations for interpretation** | | |
| --- | --- | --- | --- | --- | --- | --- | --- | --- | --- |
| **Study; sample size; type of cancer n (%)** | **Treatment group (TG)** | **Comparator group (CG)** | **What was measured?** | **Outcome measure/tool used** | **Values, significance; or narrative description** | **Direction of findings** | **Sample size calculation, primary outcome, end timepoint specified (if >1 follow-up timepoints); baseline outcome measure variance; floor/ceiling effect bias; adjustment for between-group differences on key characteristics and baseline outcomes** | **Adequate statistical analysis (ITT, per protocol, both, or unclear; multiple comparisons without adjustment)** | **TIDieR score (range 0-12); quality of intervention (dose response issues); interpretation of intervention effect (in relation to timing of delivery and of outcome assessment)** |
| **1 RCT, between-group comparisons** | | | | | | | | | |
| Sun 2023 [18];  n=130;  Cervical (100%) | PFM therapy (active) + education | Routine nursing care | Pelvic floor distress; impact on quality of life | PFDI-20; PFIQ-7 | Post-intervention mean±SD:  PFDI-20: TG 111±7 vs CG 136±11, p<0.001*;  PFIQ-7: TG 121±6 vs CG 147±9, p<0.001* | Difference in favor of TG | No sample size calculation provided;  Primary outcomes specified: PF distress (PFDI-20) and PF impact (PFIQ-7) | ITT;  Multiple comparisons without adjustment | 7;  Unclear duration of intervention; Short-term follow-up; Time after cancer treatment NR |
| **1 non-RCT with 2 groups, between-group comparisons** | | | | | | | | | |
| Li 2023 [29];  n=120;  Cervical (100%) | PFM therapy (active + electrostimulation) + routine care | Routine care | Pelvic floor distress | PFDI-20 | Results presented in a figure, (baseline and 2 weeks post-intervention) – estimates taken from graphics:  PFDI-20: TG 10-15 vs CG 15-20, p<0.05* | Difference in favor of TG | No sample size calculation provided; No primary outcome specified; Unclear risk of baseline outcome measure variance and floor/ceiling effect bias (no data provided) | ITT;  Multiple comparisons without adjustment | 6;  Short- and medium-term follow-up; Time since cancer diagnosis/treatment NR |
| **0 non-RCT with single group, within-group comparisons** | | | | | | | | | |
| CG: comparator group, ITT: intention-to-treat, NR: not reported, PFDI-20: Pelvic Floor Distress Inventory-20, PFIQ-7: Pelvic Floor Impact Questionnaire-7, RCT: randomized controlled trial, SD: standard deviation, TG: treatment group  *Difference reached statistical significance (p<0.05) or reached statistical significance according to methods used by the authors. | | | | | | | | | |

(e) Sexual Function

| **Sexual function** | **Intervention arms** | | **Outcomes** | | **Results** | | **Considerations for interpretation** | | |
| --- | --- | --- | --- | --- | --- | --- | --- | --- | --- |
| **Study; sample size; type of cancer n (%)** | **Treatment group (TG)** | **Comparator group (CG)** | **What was measured?** | **Outcome measure/tool used** | **Values, significance; or narrative description** | **Direction of findings** | **Sample size calculation, primary outcome, end timepoint specified (if >1 follow-up timepoints); baseline outcome measure variance; floor/ceiling effect bias; adjustment for between-group differences on key characteristics and baseline outcomes** | **Adequate statistical analysis (ITT, per protocol, both, or unclear; multiple comparisons without adjustment)** | **TIDieR score (range 0-12); quality of intervention (dose response issues); interpretation of intervention effect (in relation to timing of delivery and of outcome assessment)** |
| **5 RCTs, between-group comparisons** | | | | | | | | | |
| Li 2016 [21];  n=226;  Cervical (100%) | PFM therapy (active) + nursing education + Yoga | Conventional nursing care | Sexual function | FSFI | Mean(95%CI) difference from baseline to post-intervention: TG 2.57 (1.92 to 3.23) vs CG -2.32 (-3.01 to -1.63), p=0.000* | Difference in favor of TG | No sample size calculation provided; No primary outcome specified | ITT;  Multiple comparisons without adjustment | 9;  Short-term follow-up; Unclear time since cancer treatment (before discharge) |
| Yang 2012 [23];  n=28;  Cervical (93%)  Endometrial (7%) | PFM therapy (active) + education + core-strengthening program + hip muscle stretching exercises | Leaflet with home-based PFM exercise, lifestyle advice and a telephone number for further explanations | Sexual function | Sexual function questions from APFQ | Mean(SD) of change from baseline:  APFQ sexual function scores: TG -5.62(2.27) vs CG -2.42(3.47), p=0.047* | Difference in favor of TG | No sample size calculation provided;  Primary outcomes listed as PF function (PFM strength and motor evoked potentials of sacral nerve) and quality of life; Potential risk of baseline outcome measure variance and floor/ceiling effect bias | Appears to be ITT but selective exclusion of participants experiencing aggravation of PF symptoms and/or had some difficulty in continuing the exercises;  Multiple comparisons without adjustment | 9;  Short-term follow-up; Time since cancer treatment: median 1.2 (range 1-5) in years |
| Schofield 2020 [26];  n=318;  Endometrial/uterine (52%)  Cervical (41%)  Vulval (8%)  Vaginal (4%)  Ovarian (5%)  Fallopian tube (2%) | Education (psycho-education nurse- and peer-led psycho-educational intervention) | Usual care | Sexual function | SVQ (3 items: sexual interest, sexual worry, global sexual satisfaction) | Results for end timepoints 1 and 2 NR;  Global sexual satisfaction: compared with ratings at baseline, no significant difference across time between groups at follow-up timepoint 4 and follow-up timepoint 5, p>0.05. | No differences between groups for global sexual satisfaction (sexual interest and sexual worry NR) | Sample size based on psychological distress (HADS at end timepoints 1 and 2) (primary outcome); Potential risk of baseline outcome measure variance | ITT;  Multiple comparisons without adjustment | 11;  Unclear (low) dosage of therapy and post-study audit found variation regarding vaginal dilator management; 2 end timepoints with no data for these outcomes; Time since cancer diagnosis/treatment NR |
| Du 2020 [27];  n=69;  Cervical (100%) | Education (empowerment education-based nursing interventions for sexual function) | Conventional nursing interventions for sexual function | Sexual function | FSFI | Mean(SD) at follow-up timepoint 6 (likely during or after intervention):  Sexual satisfaction: TG 2.02(0.34) vs CG 1.73(0.25), p<0.05*;  Sexual arousal and orgasm: TG 2.19(0.39) vs CG 1.75(0.28), p<0.05*;  Vaginal lubrication and dyspareunia: TG 2.07(0.42) vs CG 1.67(0.24), p<0.05*;  Sexual desire: TG 2.12(0.37) vs CG 1.69(0.29), p<0.05* | Difference in favor of TG | No sample size calculation provided; No primary outcome identified; Sexual function decreased over time in both groups, but TG scores were significantly superior, indicating better sexual function, than CG | ITT;  Multiple comparisons without adjustment | 5;  Unclear dosage of intervention; Unclear follow-up timepoint; Time since cancer diagnosis/treatment NR |
| Robinson 1999 [28];  n=32;  Cervical (75%)  Endometrial (25%) | Education (psychoeducation group sessions;  with information on lubricants, dilators, Kegel exercises) | Same booklet as in TG | Sexual function | Sexual History Form | Sexual History Form global score: data NR but represented in figures, p>0.05. | No differences between groups | No sample size calculation provided; Primary outcome specified: sexual function (Sexual History Form); Potential risk of baseline outcome measure variance | Both;  Multiple comparisons, with p=0.01 for secondary outcomes | 10;  Potentially low dosage and no data provided on adherence to recommendations; Unclear follow-up; Time since cancer diagnosis/ treatment NR |
| **1 non-RCT with 2 groups, between-group comparisons** | | | | | | | | | |
| Tung 2024 [30];  n=63;  Cervical (100%) | Education (transtheoretical model-based sexual health education program) | Traditional sexual health education | Sexual attitudes; Sexual self-efficacy | 25-item gynecological cancer - sexual health attitudes (SA); 27-item gynecological cancer - sexual self-efficacy (SSE) | Mean;  Sexual attitudes:  1 week post-intervention: TG 94.55 vs CF 83.90, p<0.001*;  6 weeks post-intervention: TG  94.23 vs CG 85.00, p<0.01*;  Sexual self-efficacy  1 week post-intervention: TG 99.36 vs 92.38, p>0.05;  6 weeks post-intervention: TG 104.32 vs CG 87.27, p<0.05* | Mixed;  Difference in favor of TG for sexual attitudes (both short- and medium-term follow-up) and sexual self-efficacy at medium-term follow-up only | Sample size based on effect size of 0.4, power of 80% and level of significance of 0.05; Sample size not achieved; No primary outcome specified (likely sexual function outcomes); Unclear risk of floor/ceiling effect bias (no data provided) | ITT | 9;  Potentially low dosage; Short- and medium-term follow-up; Time since cancer diagnosis NR |
| **4 non-RCTs with single group, within-group comparisons** | | | | | | | | | |
| Cyr 2020, 2021, 2022a, 2022b [10, 31-33];  n=31;  Endometrial (64.5%)  Cervical (35.5%) | PFM therapy (active + passive) + education | NA | Dyspareunia pain intensity; Dyspareunia pain quality; Sexual function; Sexual matters; Sexual distress; Painful intercourse self-efficacy (pain, sexual function, other symptoms) | NRS; MPQ; FSFI; Frequency of sexual activities with vaginal penetration; ICIQ-VS (sexual matters); FSDS-R; PISES | Mean±SD or Median (Q1 to Q3) change from baseline to 2 weeks post-intervention:  NRS: -5.6±2.2, p˂0.001*;  MPQ: -12.9±14.7, p˂0.001*;  FSFI total score: 6.9±6.4, p˂0.001*;  FSFI desire: 0.9±1.3, p˂0.001*;  FSFI arousal: 0.5±1.1, p=0.040*;  FSFI lubrication: 0.7±1.4, p=0.017*;  FSFI orgasm: 0.8±1.8, p=0.044*;  FSFI satisfaction: 1.6±1.5, p˂0.001*;  FSFI pain: 2.3±1.2, p˂0.001*;  Frequency of sexual activities with vaginal penetration (per month): 1.6±1.9, p˂0.001*;  ICIQ-VS sexual matters: -24.0 (-35.3; -12.5), p˂0.001*;  FSDS-R: -13.1±11.9, p<0.001*;  PISES (pain, sexual function, other symptoms): 21.4±22.9, 24.8±18.1, 24.9±19.9, p<0.001*;  Mean(95%CI) change from baseline to 12 months follow-up:  NRS: -4.6 (-5.7 to -3.6), p˂0.001*;  MPQ: -14.4 (-20.5 to -8.3), p˂0.001*;  FSFI total score: 4.6 (1.0 to 8.1), p=0.009*;  ICIQ-VS sexual matters: -22.8 (-32.3 to -13.4), p˂0.001*;  FSDS-R: -10.0 (-15.7 to -4.4), p<0.001*;  PISES: 17.1 (10.1 to 24.1), p<0.001* | Improvement in all outcomes | Sample size calculation provided, but not for this outcome – based on feasibility outcomes and sample size achieved | ITT;  Multiple comparisons, no adjustment for pre-post data but Bonferroni correction for follow-up data | 11;  Short- and long-term follow-up; Time since cancer treatment: median 38 (Q1 9; Q3 70) in months |
| Bober 2018 [37];  Hungr 2020 [38];  n=53; Ovarian (100%) | Education (psychoeducational intervention -  sexual therapy and rehabilitation) | NA | Sexual function; Sexual self-efficacy | FSFI; SSES | Mean(SD):  FSFI total score: baseline 13.43(7.25), 2 months post-intervention 17.03(8.59), and 6 months post-intervention 16.50 (9.13), p<0.05*;  FSFI desire: baseline 2.30(1.04), 2 months post-intervention 2.59(0.96), and 6 months post-intervention 2.63(1.14), p=0.003* for 6 months only;  FSFI arousal: baseline 2.30(1.54), 2 months post-intervention 3.09(1.78), and 6 months post-intervention 2.84(1.87), p<0.01*;  FSFI lubrication: baseline 2.01(1.54), 2 months post-intervention 2.71(1.71), and 6 months post-intervention 2.73(1.91), p<0.05*;  FSFI orgasm: baseline 2.10(1.69), 2 months post-intervention 3.04(2.03), and 6 months post-intervention 2.86(2.07), p<0.05*;  FSFI pain: baseline 2.13(2.04), 2 months post-intervention 2.19(2.19), and 6 months post-intervention 2.21(2.22), p=0.135;  FSFI satisfaction: baseline 2.76(1.48), 2 months post-intervention 3.41(1.66), and 6 months post-intervention 3.23(1.70), p<0.01*;  Sexual self-efficacy: baseline 61.63(19.19), and 2 months post-intervention 76.31(17.25), p=0.001* | Mixed;  Improvement in all outcomes, except for desire (improvement at 6 months only) and sexual self-efficacy at 6 months (NR);  No improvement in pain | No sample size calculation provided; Primary outcome specified: sexual function (FSFI), psychological distress (BSI-18) and participant satisfaction | ITT;  Multiple comparisons without adjustment | 9;  Potentially adequate dosage; Medium-term follow-up; Time since cancer diagnosis mean 6 (SD 5) in years;  Time since cancer treatment NR |
| Brotto 2012 [39];  n=31;  Endometrial (65%)  Cervical (26%) Both (10%) | Education (psychoeducational intervention) | NA | Sexual function; Sexual distress; Perception of genital arousal; Subjective sexual arousal | FSFI; FSDS; SFQ treatment impact; 34-item self-report questionnaire that measures autonomic arousal, perception of genital arousal, overall subjective arousal, anxiety, negative affect, positive affect and mental sexual arousal change (1-7 Likert scale - from neutral to erotic stimuli) | Mean(SD):  FSFI desire: baseline 1.82(0.92), post-intervention 2.94(1.41), and 6 months post-intervention 2.75(1.25), p<0.001* (only post-intervention);  FSFI arousal: baseline 3.00(1.10), post-intervention 4.47(1.35), and 6 months post-intervention 4.00(1.36), p<0.001* (only post-intervention);  FSFI lubrication: baseline 2.70(1.64), post-intervention 4.42(1.16), and 6 months post-intervention 3.6(1.59), p<0.001* (only post-intervention);  FSFI orgasm: baseline 3.38(1.65), post-intervention 4.40(1.45), and 6 months post-intervention 4.46(1.51), p<0.001* (only post-intervention);  FSFI satisfaction: baseline 2.91(1.18), post-intervention 4.07(1.48), and 6 months post-intervention 3.51(1.43), p<0.001* (only post-intervention);  FSFI pain: baseline 3.78(1.96), post-intervention 4.89(1.61), and 6 months post-interventionn4.18(1.91), p=NS;  FSFI total score: baseline 18.36(6.57), post-intervention 26.13(5.01), and 6 months post-intervention 24.18(5.66), p<0.001* (only post-intervention);  FSDS: baseline 23.19(10.42), post-intervention 14.71(10.74), and 6 months post-intervention 17.13(11.68), p=NS;  SFQ treatment impact: baseline 3.15(0.72), post-intervention 2.75(0.96), and 6 months post-intervention 2.67(0.86), p=NS;  Perception of genital arousal (figure): baseline 1.5(0.25) and post-intervention 2(0.25), p=0.014 (NS);  Subjective sexual arousal (figure): baseline 1.75(0.25) and post-intervention 1.5(0.25), p=NS | Mixed;  Improvement in sexual function, desire, arousal, lubrication, orgasm, and satisfaction post-intervention only;  No improvement in pain, sexual distress, perception of genital arousal, and subjective sexual arousal | No sample size calculation provided; Primary outcome specified: sexual function (FSFI); Unclear risk of baseline outcome measure variance and floor/ceiling effect bias for some outcomes | Unclear; Multiple comparisons without adjustment, except self-report items where p=0.0045 (0.05/11 analyses) | 6;  Potentially adequate and low dosage for some outcomes and potential mismatch between intervention (education) and outcome (physiological-related); Short- and medium-term follow-up; Time since cancer treatment NR |
| Brotto 2008 [40];  n=22;  Cervical (59%) Endometrial (41%) | Education (brief mindfulness-based cognitive behavioral intervention) | NA | Sexual function; Sexual distress; Perceived genital/physical arousal; Subjective sexual arousal; Perceived autonomic arousal | FSFI; FSDS; DASA; 34-item self-report questionnaire that measures autonomic arousal, perception of genital arousal, overall subjective arousal, anxiety, negative affect, positive affect and mental sexual arousal change (1-7 Likert scale - from neutral to erotic stimuli) | FSFI desire: baseline 2.43(0.22) and post-intervention 3.37(0.20), p<0.01*;  FSFI arousal: baseline 3.35(0.49) and post-intervention 4.48(0.38), p<0.01*;  FSFI lubrication: baseline 3.43(0.59) and post-intervention 3.79(0.50), p=NS;  FSFI orgasm: baseline 3.06(2.34) and post-intervention 4.56(1.87), p<0.01*;  FSFI satisfaction: baseline 3.95(0.43) and post-intervention 4.75(0.31), p<0.01*;  FSFI pain: baseline 3.40(0.57) and post-intervention 3.81(0.56), p=NS;  FSFI total score: baseline 20.19(10.53) and post-intervention 25.39(7.22), p<0.05*;  FSDS: baseline 22.72(11.94), post-intervention 15.06(10.43), p<0.001*;  DASA mental sexual excitement (figure): baseline 4.25 and post-intervention 5, p=0.002*;  DASA genital tingling/throbbing: baseline 4.25 and post-intervention 5, p=0.029*;  DASA pleasant sexual genital sensations: no data provided, p=0.019*;  Perceived genital/physical arousal (figure): baseline 4-5 and post-intervention 6-7, p<0.05 (NS);  Subjective sexual arousal (figure): baseline 2 and post-intervention 3, p=NS;  Perceived autonomic arousal (figure): baseline 4 and post-intervention 7, p=NS | Mixed;  Improvement in sexual function, desire, arousal, orgasm, satisfaction, and sexual distress;  No improvement for lubrication, pain, perceived genital/physical arousal, subjective sexual arousal, and perceived autonomic arousal | No sample size calculation provided; Primary outcome specified: sexual arousal (DASA from session 1 to session 4); Unclear risk of baseline outcome measure variance and floor/ceiling effect bias for some outcomes | Unclear; Multiple comparisons without adjustment, except Bonferroni correction to Film Scale where p=0.008 (0.05/6 analyses) | 8;  Potentially adequate and low dosage for some outcomes and potential mismatch between intervention (education) and outcome (physiological-related); Short-term follow-up; Time since cancer treatment: mean 54 (range 6-115) in months |
| APFQ: Australian Pelvic Floor Questionnaire, CG: comparator group, CI: confidence interval, DASA: Detailed Assessment of Sexual Arousal, FSDS(-R): Female Sexual Distress Scale(-Revised), FSFI: Female Sexual Function Index, ICIQ-VS: International Consultation on Incontinence Questionnaire - Vaginal Symptoms, ITT: intention-to-treat, MPQ: McGill Pain Questionnaire, NA: not applicable, NR: not reported, NS: non-significant, NRS: Numerical Rating Scale, PFM: pelvic floor muscle, PISES: Painful Intercourse Self-Efficacy Scale, Q1: first quartile, Q3: third quartile, RCT: randomized controlled trial, SD: standard deviation, SFQ: Sexual Function Questionnaire, SVQ: Sexual function-Vaginal changes Questionnaire, TG: treatment group  *Difference reached statistical significance (p<0.05) or reached statistical significance according to methods used by the authors. | | | | | | | | | |

(f) Psychological Function

| **Psychological function** | **Intervention arms** | | **Outcomes** | | **Results** | | **Considerations for interpretation** | | |
| --- | --- | --- | --- | --- | --- | --- | --- | --- | --- |
| **Study; sample size; type of cancer n (%)** | **Treatment group (TG)** | **Comparator group (CG)** | **What was measured?** | **Outcome measure/tool used** | **Values, significance; or narrative description** | **Direction of findings** | **Sample size calculation, primary outcome, end timepoint specified (if >1 follow-up timepoints); outcome heterogeneity; floor/ceiling effect bias; adjustment for between-group differences on key characteristics and baseline outcomes** | **Adequate statistical analysis (ITT, per protocol, both, or unclear; multiple comparisons without adjustment)** | **TIDieR score (range 0-12); quality of intervention (dose response issues); interpretation of intervention effect (in relation to timing of delivery and of outcome assessment)** |
| **4 RCTs, between-group comparisons** | | | | | | | | | |
| Sun 2023 [18];  n=130;  Cervical (100%) | PFM therapy (active) + education | Routine nursing care | Mental states | HAMA; HAMD | Post-intervention mean±SD:  HAMA: TG 16.18±3.05 vs CG 18.49±4.14, p<0.001*;  HAMD: TG 7.80±1.27 vs CG 9.74±2.34, p=0.030* | Difference in favor of TG | No sample size calculation provided;  Primary outcomes specified: PF distress (PFDI-20) and PF impact (PFIQ-7) | ITT;  Multiple comparisons without adjustment | 7;  Unclear duration of intervention; Short-term follow-up; Time after cancer treatment NR |
| Schofield 2020 [26];  n=318;  Endometrial/uterine (52%)  Cervical (41%)  Vulval (8%)  Vaginal (4%)  Ovarian (5%)  Fallopian tube (2%) | Education (psycho-education nurse- and peer-led psycho-educational intervention) | Usual care | Psychological distress; Symptom distress | HADS; MSAS-SF-R | No significant group by time interaction effects, p>0.05. | No differences between groups | Sample size based on psychological distress (HADS at end timepoints 1 and 2) (primary outcome); Sample size met – confidence in this finding; Potential risk of baseline outcome measure variance and floor/ceiling effect bias (i.e., most participants had scores fallen into the category of mild symptoms) | ITT;  Multiple comparisons without adjustment | 11;  Unclear (low) dosage of therapy; Short- and medium-term follow-up; Time since cancer diagnosis/treatment NR |
| Du 2020 [27];  n=69;  Cervical (100%) | Education (empowerment education-based nursing interventions for sexual function) | Conventional nursing interventions for sexual function | Depression | Self-rating depression scale | Mean(SD) at follow-up timepoint 6 (likely during or after intervention):  Depression: TG 25.31(2.38) vs CG 29.05(2.49), p<0.05* | Difference in favor of TG | No sample size calculation provided; No primary outcome identified | ITT;  Multiple comparisons without adjustment | 5;  Unclear dosage of intervention; Unclear follow-up timepoint; Time since cancer diagnosis/treatment NR |
| Robinson 1999 [28];  n=32;  Cervical (75%)  Endometrial (25%) | Education (psychoeducation group sessions;  with information on lubricants, dilators, Kegel exercises) | Same booklet as in TG | Fears | Fears About Cancer and Sexuality Questionnaire | “After controlling for the other covariates, the Fear scores were 0.333 points lower in [TG] than in the [CG] (95%CI [0.051– 0.615]).” | Unclear (likely difference in favor of TG) | No sample size calculation provided; Primary outcome specified: sexual function (Sexual History Form); Potential risk of baseline outcome measure variance | Both;  Multiple comparisons, with p=0.01 for secondary outcomes (fears) | 10;  Potentially low dosage and no data provided on adherence to recommendations; Unclear follow-up; Time since cancer diagnosis/ treatment NR |
| **0 non-RCT with 2 groups, between-group comparisons** | | | | | | | | | |
| **4 non-RCTs with single group, within-group comparisons** | | | | | | | | | |
| Cyr 2020, 2021, 2022a, 2022b [10, 31-33];  n=31;  Endometrial (64.5%)  Cervical (35.5%) | PFM therapy (active + passive) + education | NA | Body image concerns; Pain anxiety (cognitive anxiety, escape/avoidance, fearful appraisal, physiological anxiety); Pain catastrophizing (rumination, magnification, helplessness); Depressive symptoms | BIS; PASS; PCS; BDI-II | Mean±SD change from baseline to 2 weeks post-intervention:  BIS: -3.6±4.3, p<0.001*;  PASS total score: -16.7±17.1, p<0.001*;  PASS cognitive anxiety: -6.4±4.3, p<0.001*;  PASS escape/avoidance: -2.1±4.9, p=0.027*;  PASS fearful appraisal: -3.8±4.5, p<0.001*;  PASS physiological anxiety: -4.5±5.0, p<0.001*;  PCS total score: -13.4±13.8, p<0.001*;  PCS rumination: -4.7±5.4, p<0.001*;  PCS magnification: -1.6±2.7, p=0.002*;  PCS helplessness: -7.1±8.0, p<0.001*;  BDI-II: -4.6±7.2, p=0.002*  Mean(95%CI) change from baseline to 12 months follow-up:  BIS: -3.4 (-5.4 to -1.3), p<0.001*;  PASS total score: -13.9 (-21.6 to -6.2), p<0.001*;  PCS total score: -12.6 (-18.1 to -7.1), p<0.001*;  BDI-II: -3.5 (-6.6 to -0.3), p=0.028* | Improvement in all outcomes | Sample size calculation provided, but not for this outcome – based on feasibility outcomes and sample size achieved | ITT;  Multiple comparisons, no adjustment for pre-post data but Bonferroni correction for follow-up data | 11;  Short- and long-term follow-up; Time since cancer treatment: median 38 (Q1 9; Q3 70) in months |
| Bober 2018 [37];  Hungr 2020 [38];  n=53; Ovarian (100%) | Education (psychoeducational intervention -  sexual therapy and rehabilitation) | NA | Somatization; Depression; Anxiety; Global severity | BSI-18 | Mean(SD):  BSI somatization: baseline 53.18(8.50), 2 months post-intervention 51.29(8.06), and 6 months post-intervention 49.44(7.43), p=0.030* for 6 months only;  BSI depression: baseline 52.36(10.09), 2 months post-intervention 48.87(9.04), and 6 months post-intervention 47.63(8.34), p=0.004*;  BSI anxiety: baseline 51.84(9.81), 2 months post-intervention 49.69(8.69), and 6 months post-intervention 48.61(7.62), p=0.063;  BSI global severity: baseline 53.09(9.07), 2 months post-intervention 49.91(8.73), and 6 months post-intervention 48.24(8.17), p=0.001* | Mixed;  Improvement in all outcomes, except for somatization (improvement at 6 months only) and sexual self-efficacy at 6 months NR);  No improvement in anxiety | No sample size calculation provided; Primary outcome specified: sexual function (FSFI), psychological distress (BSI-18) and participant satisfaction | ITT;  Multiple comparisons without adjustment | 9;  Potentially adequate dosage; Medium-term follow-up; Time since cancer diagnosis mean 6 (SD 5) in years;  Time since cancer treatment NR |
| Brotto 2012 [39];  n=31;  Endometrial (65%)  Cervical (26%) Both (10%) | Education (psychoeducational intervention) | NA | Depression | BDI | Mean(SD):  BDI: baseline 10.52(7.75), post-intervention 8.32(6.92), and 6 months post-intervention 8.32(6.50), p=NS | No improvement in outcome | No sample size calculation provided; Primary outcome specified: sexual function (FSFI); Potential risk of baseline outcome measure variance | Unclear; Multiple comparisons without adjustment, except self-report items where p=0.0045 (0.05/11 analyses) | 6;  Potentially low dosage; Short- and medium-term follow-up; Time since cancer treatment NR |
| Brotto 2008 [40];  n=22;  Cervical (59%) Endometrial (41%) | Education (brief mindfulness-based cognitive behavioral intervention) | NA | Depression | BDI | Mean(SD):  BDI: baseline 10.9(1.92) and post-intervention 6.72(1.22), p<0.01* | Improvement in outcome | No sample size calculation provided; Primary outcome specified: sexual arousal (DASA from session 1 to session 4) | Unclear; Multiple comparisons without adjustment, except Bonferroni correction to Film Scale where p=0.008 (0.05/6 analyses) | 8;  Short-term follow-up; Time since cancer treatment: mean 54 (range 6-115) in months |
| BDI: Beck Depression Inventory, BSI-18: Brief Symptom Inventory-18, CG: comparator group, CI: confidence interval, DASA: Detailed Assessment of Sexual Arousal, FSFI: Female Sexual Function Index, HADS: Hospital Anxiety and Depression Score, HAMA: Hamilton Anxiety Scale, HAMD: Hamilton Depression Scale, IQR: interquartile range, ITT: intention-to-treat, MSAS-SF-R: Memorial Symptom Assessment Scale-Short Form Revised, NA: not applicable, NR: not reported, NS: non-significant, PFDI-20: Pelvic Floor Distress Inventory-20, PFIQ-7: Pelvic Floor Impact Questionnaire-7, PF: pelvic floor, PFM: pelvic floor muscle, RCT: randomized controlled trial, SD: standard deviation, TG: treatment group  *Difference reached statistical significance (p<0.05) or reached statistical significance according to methods used by the authors. | | | | | | | | | |

(g) Quality of Life

| **Quality of life** | **Intervention arms** | | **Outcomes** | | **Results** | | **Considerations for interpretation** | | |
| --- | --- | --- | --- | --- | --- | --- | --- | --- | --- |
| **Study; sample size; type of cancer n (%)** | **Treatment group (TG)** | **Comparator group (CG)** | **What was measured?** | **Outcome measure/tool used** | **Values, significance; or narrative description** | **Direction of findings** | **Sample size calculation, primary outcome, end timepoint specified (if >1 follow-up timepoints); outcome heterogeneity; floor/ceiling effect bias; adjustment for between-group differences on key characteristics and baseline outcomes** | **Adequate statistical analysis (ITT, per protocol, both, or unclear; multiple comparisons without adjustment)** | **TIDieR score (range 0-12); quality of intervention (dose response issues); interpretation of intervention effect (in relation to timing of delivery and of outcome assessment)** |
| **7 RCTs, between-group comparisons** | | | | | | | | | |
| Li 2016 [21];  n=226;  Cervical (100%) | PFM therapy (active) + nursing education + Yoga | Conventional nursing care | Health-related quality of life | FACT-CX | Mean(95%CI) difference from baseline to post-intervention:  Overall score: TG 11.30 (9.49 to 13.11) vs CG 1.94 (0.03 to 3.85), p=0.000*;  Physical: TG 0.21 (-1.17 to 1.59) vs CG 0.12 (-1.57 to 1.33), p=0.583;  Emotional: TG 2.95 (1.92 to 3.99) vs CG 0.56 (-0.54 to 1.65), p=0.000*;  Social: TG 1.18 (0.21 to 2.15) vs CG 0.28 (-0.74 to 1.31), p=0.000*;  Functional: TG 0.69 (-1.78 to 1.64) vs CG 0.72 (-1.09 to 2.52), p=0.009*;  Cervical: TG 6.70 (5.30 to 8.09) vs CG 0.84 (-0.63 to 2.31), p=0.000* | Mixed;  Difference in favor of TG in all domains, except for physical function | No sample size calculation provided; No primary outcome specified | ITT;  Multiple comparisons without adjustment | 9;  Potentially adequate dosage; Short-term follow-up; Unclear time since cancer treatment (before discharge) |
| Yang 2012 [23];  n=28;  Cervical (93%)  Endometrial (7%) | PFM therapy (active) + education + core-strengthening program + hip muscle stretching exercises | Leaflet with home-based PFM exercise, lifestyle advice and a telephone number for further explanations | Health-related quality of life | EORTC QLQ-C30; EORTC QLQ-CX24 | EORTC-QLQ domain results presented with descriptive statistics in table and reference to within-group significance only in table and text. | Not interpretable | No sample size calculation provided;  Primary outcomes listed as PF function (PFM strength and motor evoked potentials of sacral nerve) and quality of life; Potential risk of baseline outcome measure variance and floor/ceiling effect bias | Appears to be ITT but selective exclusion of participants experiencing aggravation of PF symptoms and/or had some difficulty in continuing the exercises;  Multiple comparisons without adjustment | 9;  Short duration; Short-term follow-up; Time since cancer treatment: median 1.2 (range 1-5) in years |
| Zong 2022 [19];  n=166;  Cervical (100%) | PFM therapy (active) + clean intermittent self-catheterization | Clean intermittent self-catheterization (+ education on urinary retention and urinary incontinence) | Burden; Comfort | SPB; GCQ | Post-intervention mean±SD:  SPB burden: TG 37.59±2.78 vs CG 40.54±3.02, p<0.05*;  GCQ Comfort:  TG 74.58±4.26 vs CG 68.69±4.17, p<0.05* | Difference in favor of TG | Sample size formula provided (no detail on which outcome and follow-up timepoint were selected as primary) | ITT;  Multiple comparisons without adjustment | 7;  Short-term follow-up; Time since cancer diagnosis NR;  Time since cancer treatment: 3 days pre-surgery, 3 and 10 days post-surgery |
| Cerentini 2019 [24];  n=88;  Cervical (100%) | PFM therapy (passive) | Standard guidance from nursing team care (including re advice to use dilators) | Health-related quality of life | EORTC QLQ-C30 | EORTC QLQ-C30 (global, functional, symptoms domains) at post-intervention: no differences between TG vs CG (80±4.1 vs 79±5.1, p=0.936; 74±4.6 vs 74±6.6, p=0.9673; 24±3.6 vs 24±4.5, p=0.666) | No differences between groups | Sample size based on percentage of change in vaginal dimensions (primary outcome) in women with vaginal stenosis​ (sample size achieved for recruitment but not for follow-up measures, high attrition) | Both;  Multiple comparisons with Bonferroni post hoc tests | 8;  Potentially adequate dosage but mismatch between intervention and outcome (unlikeness to change outcome); Short-term follow-up; Time since cancer diagnosis/treatment NR |
| Jiang 2023 [25];  n=100;  Cervical (100%) | Education (routine nursing care + continuing nursing with  WeChat online education videos, seminars, including information about PFM exercise) | Routine nursing care | Health-related quality of life | FACT-CX (FACT-G + CX); QLICP-CE | Results presented in figures and no data provided. However, presence of the following statement: “During the 4th month after patients were discharged, the differences in EMD and FUD of patients in the two groups were not obvious P>0.05). In contrast, the differences in FSD, CCS, common symptoms, SSD, and SPD were obvious. The condition in [TG] was better than [CG] with significant differences and statistical significance”, where FACT-CX includes body health, social/family health-FSD, emotional health-EMD, functional health-FUD, Cx subscale-CCS) and QLICO-CE (QLICO-CE-GM) includes common symptoms, side effect dysfunction-SSD, specificity module dysfunction-SPD, among potentially others. | Mixed (and unclear);  Difference in favor of TG for some domains of quality of life (social/family health, Cx subscale, side effect dysfunction, specificity module dysfunction) | No sample size calculation provided; No primary outcome specified; Unclear risk of baseline outcome measure variance and floor/ceiling effect bias (no data provided); Disparities in terms used in methods and results to describe outcomes which could contribute to misinterpretation results | ITT;  Multiple comparisons without adjustment | 7;  Potentially adequate dosage; Short-term follow-up; Time since cancer diagnosis/treatment unclear |
| Schofield 2020 [26];  n=318;  Endometrial/uterine (52%)  Cervical (41%)  Vulval (8%)  Vaginal (4%)  Ovarian (5%)  Fallopian tube (2%) | Education (psycho-education nurse- and peer-led psycho-educational intervention) | Usual care | Health-related quality of life | FACT-G | No significant group by time (baseline and end timepoint 2) interaction effects, p>0.05. | No differences between groups | Sample size calculation provided and based on psychological distress (HADS at end timepoints 1 and 2) (primary outcome) | ITT;  Multiple comparisons without adjustment | 11;  Unclear (low) dosage of therapy; Short-term follow-up; Time since cancer diagnosis/treatment NR |
| Du 2020 [27];  n=69;  Cervical (100%) | Education (empowerment education-based nursing interventions for sexual function) | Conventional nursing interventions for sexual function | Health-related quality of life | EORTC QLQ-C30 | Mean(SD) at follow-up timepoint 6 (likely during or after intervention): TG 100.15(15.86) vs CG 85.67(12.27), p<0.000* | Difference in favor of TG | No sample size calculation provided; No primary outcome identified; significant difference between groups at baseline on quality of life – not accounted for in analysis | ITT;  Multiple comparisons without adjustment | 5;  Unclear dosage of intervention; Unclear follow-up timepoint; Time since cancer diagnosis/treatment NR |
| **0 non-RCT with 2 groups, between-group comparisons** | | | | | | | | | |
| **1 non-RCT with single group, within-group comparisons** | | | | | | | | | |
| Brotto 2008 [40];  n=22;  Cervical (59%) Endometrial (41%) | Education (brief mindfulness-based cognitive behavioral intervention) | NA | Quality of life | SF-36 | SF-36 physical composite: no data provided, p=NS;  SF-36 mental health: no data provided, p<0.001 | Mixed;  Improvement in mental health;  No improvement in physical health | No sample size calculation provided; Primary outcome specified: sexual arousal (DASA from session 1 to session 4); Unclear risk of baseline outcome measure variance and floor/ceiling effect bias (no data provided) | Unclear; Multiple comparisons without adjustment, except Bonferroni correction to Film Scale where p=0.008 (0.05/6 analyses) | 8;  Potentially adequate dosage; Short-term follow-up; Time since cancer treatment: mean 54 (range 6-115) in months |
| CG: comparator group, CI: confidence interval, DASA: Detailed Assessment of Sexual Arousal, EORTC: European Organization for Research and Treatment of Cancer Quality of Life Questionnaire (C30 vs CX24: cervical cancer module), FACT-CX: Functional Assessment of Cancer Therapy-Cervical Cancer, FACT-G: Functional Assessment for Cancer Therapy-General, FSFI: Female Sexual Function Index, GCQ: Kolcaba General Comfort Questionnaire, HADS: Hospital Anxiety and Depression Score, ITT: intention-to-treat, NA: not applicable, NR: not reported, NS: non-significant, PF: pelvic floor, PFM: pelvic floor muscle, QLICP-CE: Quality of Life in Cancer Patients - Cervical, RCT: randomized controlled trial, SD: standard deviation, SPB: Self-Perceived Burden Scale, TG: treatment group  *Difference reached statistical significance (p<0.05) or reached statistical significance according to methods used by the authors. | | | | | | | | | |

(h) PFM Function

| **PFM function** | **Intervention arms** | | **Outcomes** | | **Results** | | **Considerations for interpretation** | | |
| --- | --- | --- | --- | --- | --- | --- | --- | --- | --- |
| **Study; sample size; type of cancer n (%)** | **Treatment group (TG)** | **Comparator group (CG)** | **What was measured?** | **Outcome measure/tool used** | **Values, significance; or narrative description** | **Direction of findings** | **Sample size calculation, primary outcome, end timepoint specified (if >1 follow-up timepoints); outcome heterogeneity; floor/ceiling effect bias; adjustment for between-group differences on key characteristics and baseline outcomes** | **Adequate statistical analysis (ITT, per protocol, both, or unclear; multiple comparisons without adjustment)** | **TIDieR score (range 0-12); quality of intervention (dose response issues); interpretation of intervention effect (in relation to timing of delivery and of outcome assessment)** |
| **4 RCTs, between-group comparisons** | | | | | | | | | |
| Li 2019 [20];  n=91;  Cervical (100%) | PFM therapy (electrostimulation) + intermittent catheterization + bladder function training | Intermittent catheterization + bladder function training | Strength: deep and superficial PFM (dPFM, sPFM) | GRUGG, via digital palpation | Mean±SD at post-treatment:  dPFM sustained strength: TG 2.98±0.56 vs CG 1.88±0.63, p=0.000*;  dPFM strength on repeated rapid contractions: TG 3.08±0.58 vs CG 1.98±0.60, p=0.000*;  sPFM sustained strength: TG 2.92±0.58 vs CG 2.05±0.58, p=0.000*;  sPFM strength on repeated rapid contractions: TG 2.75±0.60 vs CG 2±0.58, p=0.000* | Difference in favor of TG | No sample size calculation provided; No primary outcome specified; No baseline values (uncertainty of effect of intervention); Groups for dPFM sustained strength were different at baseline (2.06±0.67 vs 1.93±0.63, p=0.039) – not accounted for in analysis; Groups for sPFM strength on repeated rapid contractions were different at baseline (2.0±0.74 vs 2.05±0.53, p=0.036) – not accounted in analysis | ITT;  Multiple comparisons without adjustment | 6;  Short-term follow-up; Time since cancer treatment: 11 days |
| Rutledge 2014 [22];  n=40;  Uterine (60%)  Ovarian (23%)  Cervical (13%) | PFM therapy (active) + education | Usual care (no intervention) | PFM strength | Brink, via digital palpation | - | Not interpretable | Sample size based on improvement in Patient Global Impression of Improvement (primary outcome); Unclear risk of baseline outcome measure variance and floor/ceiling effect bias (no data provided) | ITT | 11;  Short-term follow-up; Time since cancer treatment: median 2.5 (range 1-5) in years |
| Yang 2012 [23];  n=28;  Cervical (93%)  Endometrial (7%) | PFM therapy (active) + education + core-strengthening program + hip muscle stretching exercises | Leaflet with home-based PFM exercise, lifestyle advice and a telephone number for further explanations | PFM strength; Sacral nerve function | Vaginal squeeze pressure (cmH_2_O), via manometry; Sacral and transcranial magnetic stimulation + intra-anal sponge electrode (MEP) | Mean(SD) difference from baseline to post-intervention:  cmH_2_O: TG 21.78(7.64) vs CG 7.56(8.65), p=0.004*;  Sacral stimulation  latency: TG -0.41(0.88) vs CG -0.03(0.27), p=0.283;  Sacral stimulation  amplitude: TG 0.30(0.27) vs CG 0.05(0.20), p=0.142;  Sacral stimulation  threshold: TG -32.00(9.75) vs CG -11.71(14.24), p=0.010*;  Cranial stimulation at rest  latency: TG -0.015(0.02) vs CG 3.93(1.72), p=0.731;  Cranial stimulation at rest  latency amplitude: TG -0.08(0.20) vs CG 0.00(0.29), p=0.295;  Cranial stimulation at rest  latency threshold: TG -19.40(14.29) vs CG -9.40(19.67), p=0.548;  Cranial stimulation with facilitation latency: TG 0.15(1.58) vs CG −1.06(4.57), p=0.731;  Cranial stimulation with amplitude: TG −0.20(0.42) vs CG −0.22(0.83), p=0.445;  Cranial stimulation with threshold: TG −12.50(21.79) vs CG −9.67(11.69), p=0.914 | Mixed;  Difference in favor of TG for PFM strength and sacral MEP threshold (reduced threshold: increased sacral excitability) only | Small sample size; No sample size calculation provided;  Primary outcomes listed as PF function (PFM strength and motor evoked potentials of sacral nerve) and quality of life; Potential risk of baseline outcome measure variance and floor/ceiling effect bias | Appears to be ITT but selective exclusion of participants experiencing aggravation of PF symptoms and/or had some difficulty in continuing the exercises;  Multiple comparisons without adjustment | 9;  Short duration; Short-term follow-up; Time since cancer treatment: median 1.2 (range 1-5) in years |
| Cerentini 2019 [24];  n=88;  Cervical (100%) | PFM therapy (passive) | Standard guidance from nursing team care (including re advice to use dilators) | PFM function | Contractility/relaxation capacity, measured via digital palpation, using % for hypoactive/normal/hyperactive scale | Proportion at post-intervention:  Hypoactive TG 59% vs CG 78%,  Normal TG 24% vs CG 22%, Hyperactive TG 18% vs CG 0%,  p=0.210 | No differences between groups | Sample size based on percentage of change in vaginal dimensions (primary outcome) in women with vaginal stenosis​ (sample size achieved for recruitment but not for follow-up measures, high attrition); Unclear criteria of categories considering assessment tool used | Both;  Multiple comparisons with Bonferroni post hoc tests | 8;  Potentially adequate dosage but adherence to protocol declined and high attrition in both groups; Short-term follow-up; Time since cancer diagnosis/treatment NR |
| **0 non-RCT with 2 groups, between-group comparisons** | | | | | | | | | |
| **4 non-RCTs with single group, within-group comparisons** | | | | | | | | | |
| Cyr 2020, 2021, 2022a, 2022b [10, 31-33];  n=31;  Endometrial (64.5%)  Cervical (35.5%) | PFM therapy (active + passive) + education | NA | PFM function | Transperineal ultrasound imaging; Intra-vaginal dynamometric speculum | Mean(SD) change from baseline to 2 weeks post-intervention:  Rest:  Bladder neck position – x-axis (cm): 0.1(0.5), p=0.50;  Bladder neck position – y-axis (cm): 0(0.2), p=0.70;  Anorectal angle (◦): 7.5(6.3), p<0.001*;  Levator plate angle (◦): -3.9(4.7), p<0.001*;  Levator hiatal area (cm^2^): 2.0(2.5), p<0.001*;  Levator hiatal anterior-posterior diameter (cm): 0.3(0.4), p<0.001*;  Levator hiatal left-right diameter (cm): 0.3(0.3), p<0.001*;  Maximal contraction: Bladder neck position – x-axis (cm): -0.2(0.5) cm, p=0.032*; Bladder neck position – y-axis (cm): 0.1(0.3), p=0.029*; Anorectal angle (◦): -2.6(7.8), p=0.10;  Levator plate angle (◦): 0.6(4.7), p=0.51;  Levator hiatal area (cm^2^): -0.6(1.3), p=0.032*;  Levator hiatal anterior-posterior diameter (cm): -0.1(0.3), p=0.022*;  Levator hiatal left-right diameter (cm): 0(0.3), p=0.46  Excursion:  Ventral displacement – x-axis (cm): −0.3(0.3), p<0.001*;  Cranial displacement – y-axis (cm): 0.1(0.2), p=0.011*; Ventrocranial displacement (cm): 0.3(0.3), p<0.001*; Anorectal angle excursion (◦): 10.2(10.0), p<0.001*;  Levator plate angle excursion (◦): 5.5(7.4), p<0.001*;  Levator hiatal area narrowing (%): 14.4(9.8), p<0.001*;  Levator hiatal anterior–posterior diameter reduction (%): 7.2(6.5), p<0.001*;  Levator hiatal left-right diameter reduction (%): 9.0(7.9), p<0.001*;  Intra-vaginal dynamometric speculum:  Initial passive resistance at minimal vaginal aperture (11-mm aperture) - Passive forces (N): −0.4(0.7), p=0.006*;  Passive resistance at maximal vaginal aperture - Passive forces (N): 3.8(5.4), p<0.001*;  Maximal aperture (mm): 9.0(8.3), p<0.001*;  Passive forces at 15-mm aperture (N): −0.6(1.2), p=0.017*;  Passive elastic stiffness at 15-mm aperture (N/mm): -0.1(0.2), p=0.006*;  Vaginal aperture at a common force of 2 N (mm): 2.5(4.1), p=0.005*; Hysteresis (N × mm): 62.7(72.8) p<0.001*;  Maximal strength at 15-mm aperture (N): 0.7(2.6), p=0.13; Number of rapid contractions: 3(4), p<0.001*; Ascending slope: 3.1(6.2), p=0.012*; Descending slope: -2.4(5.4), p=0.027*;  Endurance on 50 seconds (%*s): 683(760), p<0.001* | Mixed;  Transperineal ultrasound imaging at rest: increase in anorectal angle, decrease in levator plate angle, and increase in levator hiatal dimensions, suggestive of reduced PFM tone at rest;  Transperineal ultrasound imaging at maximal contraction: higher bladder neck position and decrease in levator hiatal area and antero-posterior diameter, indicative of improved PFM contraction;  Transperineal ultrasound imaging (excursion from rest to maximal contraction): improvement in all outcomes, implying either or a combination of reduced PFM tone and improved PFM contraction;  Intra-vaginal dynamometric speculum:  improvement in all outcomes, except PFM strength | Sample size calculation provided, but not for this outcome – based on feasibility outcomes and sample size achieved | ITT;  Multiple comparisons, no adjustment for pre-post data but Bonferroni correction for follow-up data | 11;  Potentially adequate dosage; Short-term follow-up; Time since cancer treatment: median 38 (Q1 9; Q3 70) in months |
| Brennen 2023 [34];  n=36;  Endometrial/uterine (69%) Cervical (19%) Ovarian (11%) | PFM therapy (active) + education | NA | PFM maximal voluntary contraction; PFM endurance | Squeeze pressure (mmHg), via femfit intravaginal sensor | Mean(95%CI) change from baseline to post-intervention:  PFM maximal voluntary contraction (mmHg): 0.16 (-5.0 to 4.5), p=NR;  Endurance: 0.42 (-1.0 to 1.7), p=NR | Improvement in all outcomes, but confidence intervals cross the line of no effect | Sample size calculation provided, but not for this outcome – based on feasibility outcomes and sample size achieved; Unclear risk of baseline outcome measure variance and floor/ceiling effect bias | ITT;  Multiple comparisons without adjustment | 11;  Potentially adequate dosage; Short- and medium-term follow-up; Time since cancer treatment: median 17.5 (IQR 27) in months |
| Bernard 2021 [35];  n=8;  Endometrial (100%) | PFM therapy (active) + education | NA | PFM function | Transperineal ultrasound imaging; Intra-vaginal dynamometer | Mean:  Transperineal ultrasound imaging;  Levator plate length at rest (mm): baseline 55.9 and post-intervention 53.6, p=0.11;  Levator plate length during maximal contraction (mm): (mm): baseline 44.7 and post-intervention 43.9, p=0.73;  Levator plate length during maximal Valsalva (mm): baseline 61.7 and post-intervention 59.2, p=0.22;  “UL” (mm): baseline 26.0 and post-intervention 28.1, p=0.18;  Intra-vaginal dynamometer:  Passive resistance (baseline) (N): baseline 1.08 and post-intervention 1.23, p=0.45;  Passive resistance (maximal relative peak) (N): baseline 4.87 and post-intervention 4.71, p=0.90;  Passive resistance (decline between maximal and final relative peaks) (%): baseline -23.99 and post-intervention -22.42, p=0.42;  Stiffness (N/mm): baseline 0.49 and post-intervention 0.47, p=0.90;  Maximal (contraction) relative force (N): baseline 5.75 and post-intervention 6.91, p=0.09;  Rate of force development (N/s): baseline 11.28 and post-intervention 14.52, p=0.05*;  Quick contraction test (no. of peaks): baseline 5.6 and post-intervention 5.3, p=0.53;  Quick contraction test (maximal relative force) (N): First contraction: baseline 4.44 and post-intervention 6.65, p<0.0001*;  Rate of force development (N/s): First contraction: baseline 12.66 and post-intervention 19.27, p=0.0006*;  Endurance relative peak (N): baseline 5.14 and post-intervention 7.94, p<0.0001*;  Time for the duration >50% MVC (s): baseline 5.70 and post-intervention 10.53, p=0.41;  Area under the curve >50% MVC (N*s): baseline 14.15 and post-intervention 35.49, p=0.05* | Mixed;  Improvement in quick contraction test measures relative to first contraction and endurance measures (relative peak and area under the curve >50% MVC);  No improvement in all transperineal ultrasound imaging measures, passive resistance measures, PFM strength, quick contraction test for no. of peaks, and endurance measure (time for the duration of >50% MVC) | Small sample size; No sample size calculation provided;  Primary outcome specified: urinary incontinence (pad test); Unclear risk of baseline outcome measure variance and floor/ceiling effect bias | ITT;  Multiple comparisons without adjustment | 11;  Potentially adequate dosage but limited information on adherence and report of variation in total number of training sessions recorded (24 to 124 sessions per participant); Short-term follow-up; Time since cancer treatment: mean 43 (range 19-55) in months |
| Sacomori 2020 [36];  n=49;  Cervical (100%) | PFM therapy (active) | NA | PFM strength; PFM activation | MOS, via bi-digital palpation; Intravaginal EMG | Median(IQR):  PFM strength (MOS) mean: baseline  2(1) and post-intervention 2(1), p=0.052;  PFM strength (MOS) right: baseline 2(1) and post-intervention 2(1), p=0.083;  PFM strength (MOS) left: baseline 2(2) and post-intervention 2(1), p=0.095;  EMG (microvolts) minimum: baseline 1(1) and post-intervention 1(1), p=1.0;  EMG (microvolts) maximum: baseline 29(31) and post-intervention 37.5(38), p=0.196;  EMG (microvolts) mean: baseline 6(3) and post-intervention 6(5), p=0.386 | No improvement in outcomes | No sample size calculation provided;  Primary outcome specified: PFM strength (vaginal bi-digital evaluation using MOS); Unclear risk of baseline outcome measure variance and floor/ceiling effect bias | Per protocol (n=28) due to high attrition; Multiple comparisons without adjustment | 10;  Unclear duration of intervention; Unclear follow-up timepoint; Time since cancer diagnosis NR;  Time since cancer treatment up to 1 month post-radiotherapy |
| CG: comparator group, CI: confidence interval, EMG: electromyography, IQR: interquartile range, ITT: intention-to-treat, MEP: motor evoked potential, MOS: modified Oxford scale, NA: not applicable, NR: not reported, PF: pelvic floor, PFM: pelvic floor muscle (dPFM: deep PFM, sPFM; superficial PFM), RCT: randomized controlled trial, SD: standard deviation, TG: treatment group  *Difference reached statistical significance (p<0.05) or reached statistical significance according to methods used by the authors. | | | | | | | | | |
